# A Potential Lead for Insect Growth Regulator: Design, Synthesis, and Biological Activity Evaluation of Novel Hexacyclic Pyrazolamide Derivatives

**DOI:** 10.3390/molecules28093741

**Published:** 2023-04-26

**Authors:** Bingbo Guo, Biaobiao Jiang, Chunying Wang, Xiaoyu Jin, Liuyang Wang, Zhaokai Yang, Shihui Luo, Qing Yang, Li Zhang, Xinling Yang

**Affiliations:** 1 Innovation Center of Pesticide Research, Department of Applied Chemistry, College of Science, China Agricultural University, Beijing 100193, China; 2 Engineering Research Center of Plant Growth Regulator, Ministry of Education, College of Agronomy and Biotechnology, China Agricultural University, Beijing 100193, China; 3 State Key Laboratory for Biology of Plant Diseases and Insect Pests, Institute of Plant Protection, Chinese Academy of Agricultural Sciences, Beijing 100193, China

**Keywords:** insect growth regulators, pyrazolamide, ecdysone receptor, chitinase, molecular docking, dual-target IGRs

## Abstract

Ecdysone receptor (EcR) and chitinase play a critical role in the molting stage of insect pests. Each of them is considered a promising target for the development of novel insect growth regulators (IGRs). In the present paper, a total of 24 (23 novel) hexacyclic pyrazolamide derivatives were designed and synthesized by reducing the heptacycle and inserting small flexible linkers on the basis of the previously discovered dual-target compound **D-27** acting simultaneously on EcR and *Ostrinia furnacalis* chitinase (*Of*ChtI). Their insecticidal activities against *Plutella xylostella*, *Spodoptera frugiperda*, and *Ostrinia furnacalis* larvae were evaluated. The results revealed that the insecticidal activity was not significantly enhanced when the heptacycle on the pyrazole ring was reduced to a hexacycle. However, the insertion of an additional methylene spacer between the substituted phenyl ring and the amide bond can improve the insecticidal activity. Among the derivatives, the most potent compound, **6j**, exhibited promising insecticidal activities against *P. xylostella* and *S. frugiperda*. Further protein binding assays and molecular docking indicated that **6j** could target both EcR and *Of*ChtI, and is a potential lead compound for IGRs. The present work provides valuable clues for the development of new dual-target IGRs.

## 1. Introduction

Insect growth regulators (IGRs) are regarded as a class of ideal pesticides widely used for the control of pest insects, such as mosquitoes, cockroaches, and various Lepidoptera [1,2,3,4]. Compared with conventional insecticides, IGRs offer numerous advantages, such as relative nontoxicity to mammals, decreased incidence of resistance, higher selectivity, and safer eco-toxicological profiles [5,6,7]. They can target specific proteins associated with molting or metamorphosis to induce a range of abnormal growth responses in insects, from deforming or damaging post-ecdysis to failing to develop into larvae or pupae [5,8]. As molting is essential for insect growth and development, ecdysone receptor (EcR) and chitinase, involved in disturbing the process of insect molting and exfoliation, have been indicated as potential targets for the design of highly efficient and low-toxicity green insecticides [9,10,11]. Therefore, ecdysone analogues and chitinase inhibitors are considered as potential IGRs.

**Figure 1:**
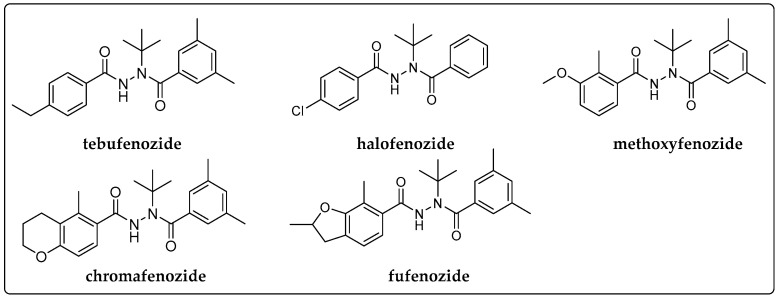
Some commercial dibenzoylhydrazine (DBH) IGRs.

Previously, some non-steroidal dibenzoylhydrazine (DBH) IGRs (Figure 1) have been commercialized [12,13]. Nevertheless, the practical application of these DBHs is limited by their single chemical structure skeleton and resistance [7,14,15]. A number of chitinase inhibitors have been reported to date, such as pentoxifylline, theophylline, caffeine, dimethylguanylurea, etc. [16]. However, no commercial chitinase inhibitors have been reported [16]. Therefore, using effective strategies to design IGRs with simpler structures and higher activity is of great value. A strategy involving the insertion of small flexible linkers is an important scaffold hopping manipulation, which has been successfully employed in the discovery of new pesticides [17].

In our previous work, we found a dual-target compound, **D-27**, could act on both EcR and *Ostrinia furnacalis* chitinase (*Of*ChtI), and the molecular hydrophobicity and flexibility play an important role on the binding affinity of ligand with the EcR or *Of*ChtI [6,18]. However, how the length of the bridge chain effects the activity is unclear. Meanwhile, it is a useful effort to discover a better IGR candidate with simpler structure and lower cost.

**Scheme 1:**
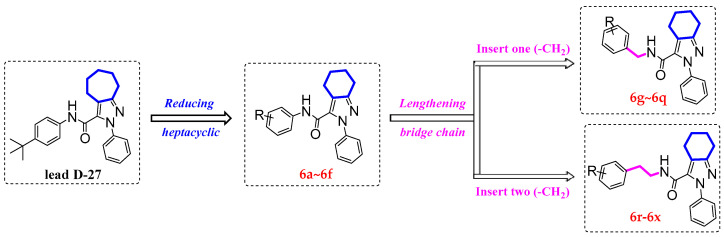
Design strategy of the target compounds.

Therefore, in the present work, a series of compounds (Scheme 1) were designed and synthesized based on the dual-target compound **D-27** by the strategy of replacing the heptacyclic ring with a smaller hexacyclic ring in order to simplify the structure and reduce the cost, as well as by involving the insertion of one or two methylene spacers to lengthen the bridge chain and improve molecular flexibility. The biological activities against *P. xylostella*, *S. frugiperda,* and *O. nubilalis*, and the protein binding assays of the compounds, were evaluated and a promising dual target compound, **6j,** with better activity than **D-27,** was hereby found.

## 2. Results and Discussion

### 2.1. Chemical Synthesis

**Scheme 2:**
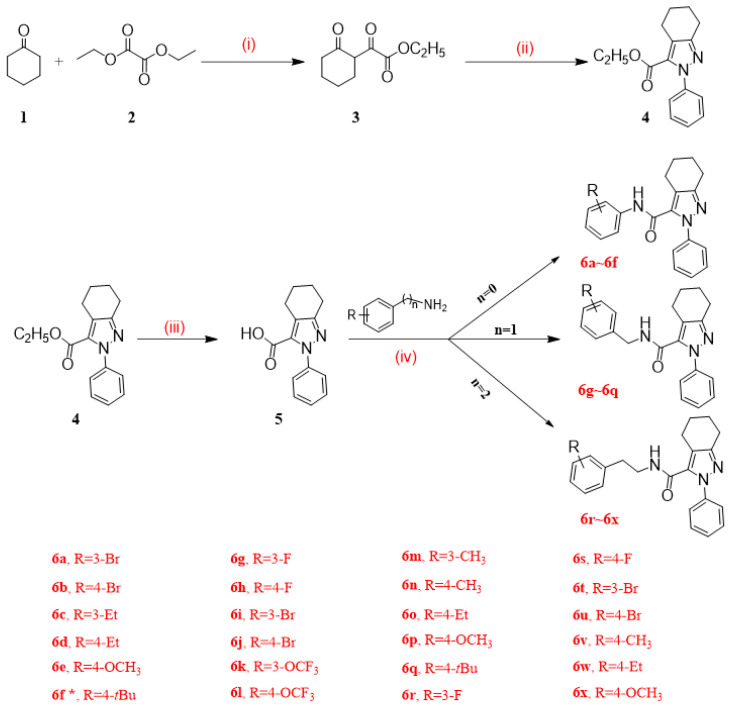
Synthetic route of target compounds **6a~6x.** Reagents and conditions: (i) EtOH, EtONa, 0 °C to room temperature (rt) (6 h); (ii) EtOH, ArN-HNH_2_ HCl, Et_3_N, rt (12 h); (iii) NaOH, 80 °C rt (2 h), HCl; (iv) DCM, HOBt, EDCI, rt (overnight). * compound has been reported in patent (CN105367498).

The synthetic route adopted for the synthesis of the target compounds is outlined in Scheme 2. The intermediate **3** was synthesized from cyclohexanone and diethyl oxalate via claisen condensation reaction. The price of cyclohexanone was significantly lower than that of cycloheptanone (Table S1), but the yield of intermediate **3** (ethyl 2-oxo-2-(2-oxocyclohexyl) acetate) (75%) was higher than that of ethyl 2-oxo-2-(2-oxocycloheptyl) acetate (68%) reported by our research group (Table S2) [18]. Thus, replacing the heptacyclic ring with a hexacyclic ring led to lower cost. Catalytic cyclization of the intermediate **3** and phenylhydrazine hydrochloride in the presence of triethylamine (TEA) produced key intermediate **4,** as described in reference [18]. The yield of intermediate **4** (ethyl 2-phenyl-4,5,6,7-tetrahydro-2H-indazole-5-carboxylate) (58%) was higher than that of ethyl 2-phenyl-2,4,5,6,7,8-hexahydrocyclohepta[c]pyrazole-5-carboxylate (50%). The pyrazole carboxylic acid **5** was prepared by the saponification reaction of intermediate **4** according to the methods in our previous work [6,18]. Finally, intermediate **5** was reacted with substituted aniline, benzylamine, or phenethylamine in the presence 1-hydroxybenzotriazole (HOBt) and 1-(3-Dimethylaminopropyl)-3-ethylcarbodiimide (EDCI) to yield the target compounds **6a~6x** [19,20]. The yields of compounds **6a~6f** were higher than those of the corresponding heptacyclic pyrazolamide compounds (Table S3), which indicates that modifying the heptacycle to a hexacycle one shows more economic viability. The structures of all target compounds were confirmed by ^1^H NMR, ^13^C NMR, and HRMS, and physical properties and structure characterization were presented in the Supplementary Materials**.**

### 2.2. Insecticidal Biological Activity

The insecticidal activities of the target compounds **6a~6x** against lepidoptera agricultural pests *P. xylostella*, *S. frugiperda,* and *O. nubilalis* were preliminarily evaluated, and the corresponding mortalities for pests were presented in Table 1. The commercial insecticide tebufenozide was selected as a positive control. The length of the bridge chain between the amide bond and the substituted phenyl ring is denoted by “**n**”. Where **n = 0**, compound with no methylene spacer; **n = 1**, compound with a methylene spacer; **n = 2**, compound with two methylene spacers.

**Figure 2:**
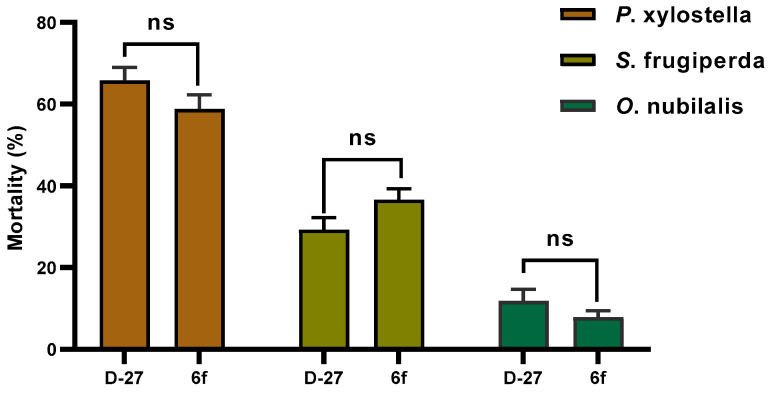
Insecticidal activity of **D-27** and **6f** against *P. xylostella*, *S. frugiperda,* and *O. furnacalis* at a concentration of 500 mg/L. Significance calculated using *t*-test, ns *p* > 0.05.

**Figure 3:**
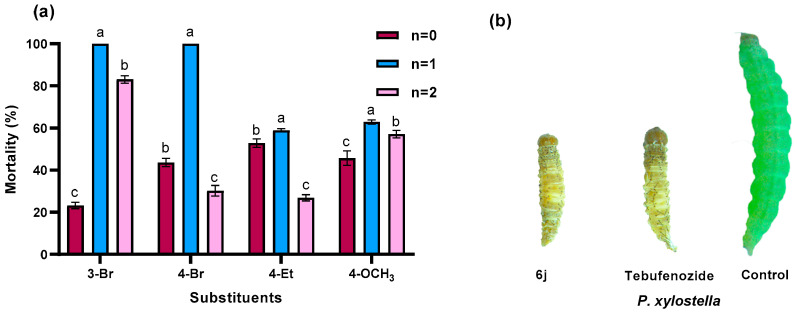
(**a**) The effect of bridge chain length on the insecticidal activity against *P. xylostella;* n represents the number of methylene spacers in the bridge chain between the amide bond and the substituted benzene ring. Data represent the mean ± standard deviation. A one-way analysis of variance (ANOVA) was performed followed by Duncan’s test (Different letters indicate values that are significantly different (*p* < 0.05)); (**b**) the abnormal molting and malformation of *P. xylostella* larvae after 96 h (From left to right: **6j**, tebufenozide, and negative control).

For *P. xylostella*, almost all tested compounds exhibited varying levels of insecticidal activity, and especially **6i**, **6j,** and **6n** displayed 100% mortality at a concentration of 500 mg/L (Table 1), which was much higher than that of the lead compound, **D-27** (65.8%), and comparable to that of the control tebufenozide (100%). However, the insecticidal activities were significantly reduced when the concentration was decreased to 200 mg/L. Compound **6j** showed the best insecticidal activity among these compounds, with a mortality rate of 40.3%. Based on the structure–activity study, no significant differences in insecticidal activity were observed when the heptacyclic compound on the pyrazole ring was reduced to a hexacyclic one; for instance, **6f** (4-*t*Bu) ≈ **D-27** (4-*t*Bu) (Figure 2). However, the insecticidal activities varied significantly according to the number of methylene spacers in the bridge chain between the amide bond and the substituted phenyl ring, as well as the position and type of substituent on the benzene ring. As depicted in Figure 3a, the number of methylene spacers in the bridge chain had an obvious effect on the insecticidal activity. When R = 3-Br, 4-Br, 4-Et, or 4-OCH_3_, compounds with an additional methylene spacer **(n = 1)** exhibited better insecticidal activities than those without methylene **(n = 0)** or with two methylene spacers **(n = 2)**. For instance, **6i** (R = 3-Br, **n = 1**) > **6t** (R = 3-Br, **n = 2**); **6a** (R = 3-Br, **n = 0**), **6j** (R = 4-Br, **n = 1**) > **6b** (R = 4-Br, **n = 0**); and **6u** (R = 4-Br, **n = 2**) (Figure 3a). When **n = 1**, compounds with fluorine, bromine, methyl, or trifluoromethoxy substituents on the para-position of the benzene ring displayed much better insecticidal activities than those on the meta-position; for instance, **6l** (R = 4-OCF_3_, **n = 1**) > **6k** (R = 3-OCF_3_, **n = 1**) and **6n** (R = 4-CH_3_, **n = 1**) > **6m** (R = 3-CH_3_, **n = 1**).

Additionally, the appearance of the *P. xylostella* larvae treated with **6j** demonstrated some obvious symptoms of abnormal molting and malformation, such as fuscous, hindgut, coarse cuticula, and abnormal molting, similar to those induced with tebufenozide (Figure 3b). However, the size and color of the heads of *P. xylostell* treated with **6j** were different from those treated with tebufenozide (Figure 3b), which indicated that compound **6j** may act on the molt-related protein receptors, resulting in the abnormal molting of larvae.

**Figure 4:**
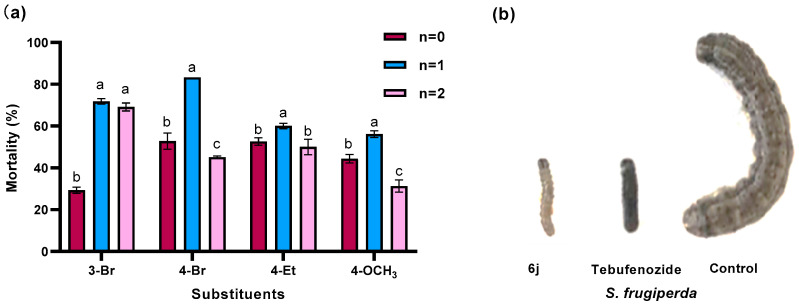
(**a**) The effect of bridge chain length on the insecticidal activity against *S. frugiperda*; n represents the number of methylene spacers in the bridge chain between the amide bond and the substituted benzene ring. Data represent the mean ± standard deviation. A one-way analysis of variance (ANOVA) was performed followed by Duncan’s test (Different letters indicate values that are significantly different (*p* < 0.05)); (**b**) the abnormal molting and malformation of *S. frugiperda* larvae after 144 h (From left to right: **6j**, tebufenozide, and negative control).

For *S. frugiperda*, all target compounds demonstrated insecticidal activity. As can be observed in Table 1, four target compounds (**6h**, **6i**, **6j**, and **6t**) exhibited promising insecticidal activities (68.7−83.3% mortality) at a concentration of 500 mg/L, much higher than the lead compound **D-27** (29.3%). The analysis of preliminary structure–activity relationships (SARs) of the target compounds showed that the insecticidal activity was not significantly enhanced when the heptacycle on the pyrazole ring was modified to a hexacycle (Figure 2). It was worth noting that the number of methylene spacers in the bridge chain, as well as the position and type of substituents on the benzene ring, had an obvious effect on the insecticidal activity (Figure 4a). When **n = 1**, the insecticidal efficacies were higher than those of the corresponding compounds with **n = 0** or **2**. For instance, **6p** (R = 4-OCH_3_, **n = 1**) > **6e** (R = 4-OCH_3_, **n = 0**) and **6x** (R = 4-OCH_3_, **n = 2**) (Figure 4a). Meanwhile, derivatives with an electron-withdrawing substituent at para-position (such as R = 4-F and 4-Br) seemed to have higher potency than those with an electron-donating substituent (such as R = 4-CH_3_ and 4-Et,4-OCH_3_). For instance, **6h** (R = 4-F, **n = 1**) > **6n** (R = 4-CH_3_, **n = 1**) and **6j** (R = 4-Br, **n = 1**) > **6o** (R = 4-Et, **n = 1**).

Furthermore, the growth and development of the *S. frugiperda* larvae treated with **6j** were dramatically inhibited, and their appearance also demonstrated some obvious symptoms of abnormal molting and malformation similar to those induced with tebufenozide (Figure 4b). However, the color of *S. frugiperda* treated with **6j** was different from that of those treated with tebufenozide (Figure 4b), which indicated that **6j** may have a different mode of action from that of tebufenozide.

**Figure 5:**
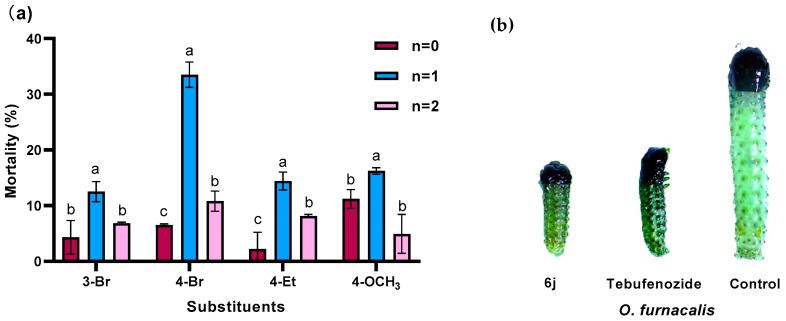
(**a**) The effect of bridge chain length on the insecticidal activity against *O. furnacalis*; n represents the number of methylene spacers in the bridge chain between the amide bond and the substituted benzene ring. Data represent the mean ± standard deviation. A one-way analysis of variance (ANOVA) was performed followed by Duncan’s test (Different letters indicate values that are significantly different (*p* < 0.05)); (**b**) the abnormal molting and malformation of *O. furnacalis* larvae after 96 h (From left to right: **6j**, tebufenozide, and negative control).

For *O. furnacalis*, most of the target compounds exhibited low insecticidal activity at a concentration of 200 mg/L. To our satisfaction, the potential compound **6j** still displayed 33.5% mortality against *O. furnacalis*. Based on the structure–activity study, we found that the compounds exhibited better insecticidal activities when **n = 1** (Figure 5a). Moreover, as depicted in Figure 5b, the growth of the *O. furnacalis* larvae treated with **6j** was greatly inhibited and its appearance also showed some obvious symptoms of abnormal molting and malformation, such as extrusion of the hindgut and cuticle fold, which was similar to that induced by tebufenozide (Figure 5b). However, the hair color of specimen treated with **6j** seems to be darker than that of those treated with tebufenozide.

### 2.3. The Binding Affinity to EcR and OfChtI

**Figure 6:**
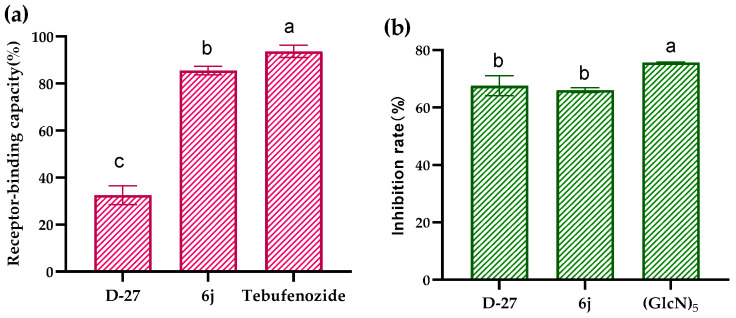
(**a**) Binding activities of **D-27**, **6j**, and tebufenozide against *PxEcR−PxUSP* at 40 mg/L; (**b**) inhibitory rates of **D-27**, **6j**, and (GlcN)**_5_** against *Of*ChtI at 40 mg/L. Data represent the mean ± standard deviation. A one-way analysis of variance (ANOVA) was performed followed by Duncan’s test (Different letters indicate values that are significantly different (*p* < 0.05)).

The growth and development of *P. xylostella, S. frugiperda,* and *O. furnacalis* treated with **6j** showed the symptoms of abnormal molting and malformation, similar to those treated with tebufenozide. Previously, **D-27** was found to have moderate binding activity to EcR/USP and excellent inhibitory activity to *Of*ChtI [6,18]. Therefore, in order to clarify the target protein of **6j**, the EcR from *P. xylostella* and chitinase from *O. furnacalis* were selected for target verification. The binding affinity of **D-27** and **6j** to EcR/USP was measured with a competitive binding assay using [^3^H] PonA as the radioactive ligand. The results revealed that **6j** had good binding activity (85.5%) at the concentration of 40 mg/L, which was better than that of **D-27** (32.5%), but slightly lower than that of tebufenozide (93.7%) (Figure 6a). The inhibiting activity of **6j** to *Of*ChtI was assayed by a microplate reader using 4-methylumbelliferyl−*N*, *N′*-diacetyl-*β-d*-chitobioside [4-MU-(GlcNAc)_2_] as a fluorescent substrate. The obtained results in Figure 6b indicated that the inhibition rate of **6j** (66.0%) was comparable to that of lead **D-27** (67.6%) at 40 mg/L, but slightly lower than that of the control (GlcN)_5_ (75.6%). On the basis of these findings, **6j** was considered as a potential dual-target insect growth regulator lead compound, due to its ability to act on both EcR/USP and *Of*ChtI receptors.

### 2.4. Molecular Docking

In order to gain insights into the possible molecular mechanisms of hexacyclic pyrazolamide derivatives binding with EcR and *Of*ChtI, **6f** and **D-27** were selected as representative compounds with which to study the effect of the modification of heptacycle into hexacycle on insecticidal activity. Thereafter, **6j** and **D-27** were selected as representative compounds to reveal the reason why **6j** was more effective than the lead **D-27** in terms of insecticidal activity. Meanwhile, **6b**, **6j**, and **6u** were selected as representative ligands with which to explore the relationship between the length of the bridge chain and the binding affinities.

**Figure 7:**
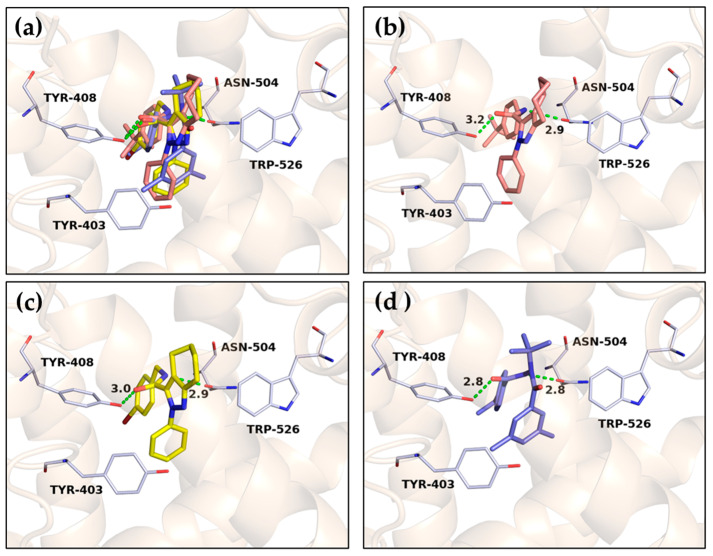
The predicted binding modes of representative ligands with EcR revealed by molecular docking. (**a**) Superimposed conformations of the molecular docking ligands; (**b**) the docking conformation of **D-27** (red); (**c**) the docking conformation of **6j** (yellow); (**d**) the docking conformation of tebufenozide (blue).

Our previous study suggested hydrogen bonding interactions and a hydrophobic effect play an important role in the design of ecdysone analogs; in particular, the hydrogen bond formed by small molecule with Tyr408 is essential for the inhibitory activity. When **6f** (hexacyclic) and **D-27** (heptacyclic) were docked into the EcR pocket, both of them showed a similar binding mode, by forming hydrogen bonds with Tyr408 and Asn504 (Figure S1a). Interestingly, **6j** and **D-27** also displayed similar binding modes to that of tebufenozide in the active site of EcR (Figure 7a), in which all three were able to form hydrogen bonds with Tyr408 and Asn504. However, the distance of the hydrogen bond formed by the carbonyl oxygen of the amide bond with Tyr408 in compound **6j** was 3.0 Å (Figure 7c), shorter than that of **D-27** (3.2 Å) (Figure 7b), but longer than that of tebufenozide (2.8 Å) (Figure 7d), which may explain the phenomenon that the binding activity of **6j** was stronger than that of **D-27**, but weaker than that of tebufenozide. The differences in the length of the bridge chain between the amide bond and the benzene ring of the same substituent could also affect the binding interactions. For example, **6b** (**n = 0**, R = 4-Br) and **6u** (**n = 2,** R = 4-Br) exhibited similar binding modes to **6j** (**n = 1**, R = 4-Br), but both could only form hydrogen bonding interactions with Asn504, and not with Tyr408 (Figure S2a).

**Figure 8:**
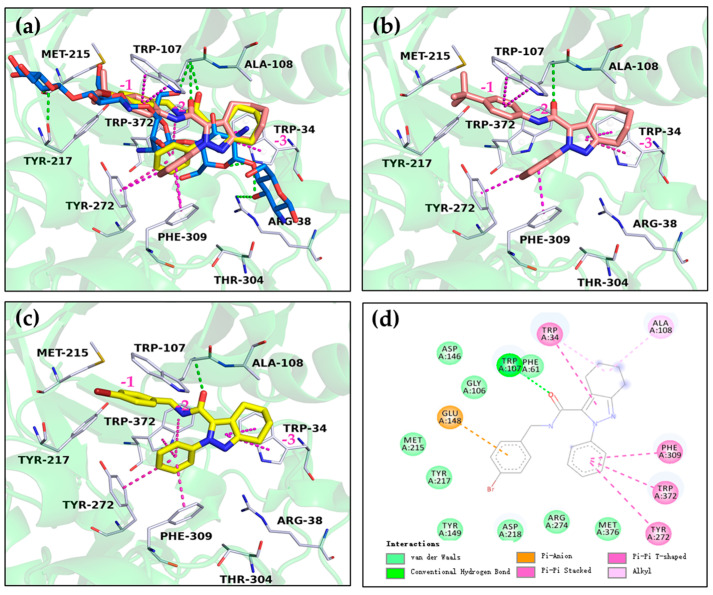
The predicted binding modes of representative ligands with *Of*ChtI. (**a**) Superimposed conformations of the molecular docking ligands; (**b**) the docking conformation of **D-27** (red); (**c**) the docking conformation of **6j** (yellow); (**d**) 2D view of **6j** in the catalytic site of *Of*ChtI.

Additionally, the docking of ligands with *Of*ChtI was carried out. The results exhibited that **6f** and **D-27** had similar binding modes in the *Of*ChtI pocket, and both can interact with Trp107 by hydrogen bond and with Trp34, Phe309, Tyr272, and Trp107 through additional π−π interactions (Figure S1b). As presented in Figure 8a, superimposed conformations of selected compounds with *Of*ChtI indicated that the binding mode of **6j** was similar to those of the substrate (GlcN)_5_ and the lead **D-27**. Specifically, **D-27** could form π−π interactions with Trp34, Phe309, and Tyr272, as well as an additional hydrogen bond with Trp107 (Figure 8b), which can also be found in the docking mode of **6j** with *Of*ChtI (Figure 8c). However, unlike **D-27, 6j** did not form π−π interactions with Trp107, but formed additional π−π interactions with the key amino acid Trp372 (Figure 8d). When R = 4-Br, the binding modes of compounds **6b** (**n = 0**, R = 4-Br) and **6u** (**n = 2**, R = 4-Br) were consistent with that of **6j** (**n = 1**, R = 4-Br), and all of them could form hydrogen bonds with Trp107 and π−π interaction with Trp372. However, **6j** could form stronger interactions with the hydrophobic residues (Phe309 and Tyr272) than **6b** and **6u** (Figure S2b). This may be why compound **6j** had higher insecticidal activity than **6b** and **6u**.

## 3. Materials and Methods

### 3.1. Instruments and Materials

All reagents (analytical grade) were obtained from commercial sources, and used without further purification. Nuclear magnetic resonance (NMR) spectra were recorded on Bruker AM-500 or AM-300 spectrometers with chloroform-d (CDCl_3_) or dimethyl sulfoxide-*d*_6_ (DMSO-*d*_6_) as the solvent and tetramethylsilane as the internal standard. High-resolution mass spectrometry (HRMS) data were obtained on a Bruker APEX IV Fourier transform HRMS (Varian, Palo Alto, CA). The melting points of all of the compounds were determined using an X-5 binocular (Fukai Instrument Co., Beijing, China) and were uncorrected. All of the target compounds were purified by column chromatography using 200–300 mesh silica gel (Puke Corporation, Qingdao, China). **D-27** (*N-*(4- (tert-butyl) phenyl)-2-phenyl-2,4,5,6,7,8-hexahydrocyclohepta[c]pyrazole-5-carboxamide) was synthesized in the laboratory.

### 3.2. General Procedure for the Synthesis of the Intermediates **3~5** and **6a~6x**

The synthetic route is depicted in Scheme 2. The spectra and high-resolution mass spectrometry (HRMS) of target compounds **6a~6x** are depicted in the Supplementary Materials.

#### 3.2.1. Synthetic Procedure of Intermediate **3**

Intermediate **3** was synthesized according to a previously described method, with minor modifications (Scheme 2) [18]. To a 0 °C solution of sodium ethoxide (0.037 mol) in 100 mL of dry ethanol, cyclohexanone (0.074 mol) and diethyl oxalate (0.074 mol) were added dropwise. The mixture was stirred for 6 h at room temperature, then acidified (pH 4-5, with 20% H_2_SO_4_) and filtered to remove the formed precipitates. The filtrate was extracted with dichloromethane, dried, and concentrated under vacuum, then purified by column chromatography (petroleum ether/ethyl acetate 30:1) to yield the *β*-keto esters **3** as a red viscous liquid (11.02 g; 75% yield). ^1^H NMR (300 MHz, DMSO-*d*_6_) *δ* 4.26–4.16 (m, 2H), 3.51–3.30 (m, 1H), 2.35 (t, *J* = 6.2 Hz, 2H), 2.17 (t, *J* = 5.7 Hz, 2H), 1.71–1.51 (m, 4H), and 1.30–1.21 (m, 3H).

#### 3.2.2. Synthetic Procedure of Intermediate **4**

The key intermediate **4** was prepared by the cyclization of the *β*-keto ester **3** with phenylhydrazine hydrochloride [6]. To a mixture of phenylhydrazine hydrochloride (0.026 mol) in ethanol (50 mL), triethylamine (TEA) was added to adjust the pH to 7. A solution of the *β-*keto esters **3** (0.026 mol) in ethanol (15 mL) was added dropwise. The mixture was stirred for 12 h at room temperature. TLC (petroleum ether/ethyl acetate 10:1) monitored the completion of the reaction. The reaction mixture was concentrated under vacuum, and the residue was purified by column chromatography (petroleum ether/ethyl acetate 10:1) to yield the intermediate **4** as a red solid (4.08 g; 58 yield). ^1^H NMR (300 MHz, CDCl_3_) *δ* 7.49–7.36 (m, 5H), 4.24 (q, *J* = 7.1 Hz, 2H), 2.85 (t, *J* = 5.9 Hz, 2H), 2.78 (t, *J* = 5.9 Hz, 2H), 1.95–1.76 (m, 4H), and 1.23 (t, *J* = 7.1 Hz, 3H).

#### 3.2.3. Synthetic Procedure of Intermediate **5**

Pyrazole carboxylic acid **5** was obtained by the saponification reaction of intermediate **4** according to the literature reporting method [12,21]. To a solution of 6 mol/L NaOH(aq) (20 mL), intermediate **4** (3.41 g) was added and the mixture was heated at 80 °C. The reaction was stirred for 2 h and monitored TLC (petroleum ether/ethyl acetate 6:1). After the completion of hydrolysis, ice water (60 mL) was added and the mixture was acidified (pH 2–3) with concentrated HCl. The formed solid was collected by filtration and dried by hot lamp to yield **5** as a white solid (2.77 g; 90% yield). ^1^H NMR (300 MHz, DMSO-*d*_6_) *δ* 13.11 (s, 1H), 7.51–7.34 (m, 5H), 2.82–2.60 (m, 4H), and 1.89–1.64 (m, 4H).

#### 3.2.4. General Procedures for the Preparation of Compounds **6a~6x** [6]

The target compound **6a** was synthesized as follows. To a solution of 1-hydroxybenzotriazole (HOBt) (2.48 mmol) and 1-(3-Dimethylaminopropyl)-3-ethylcarbodiimide (EDCI) (2.48 mmol) in dichloromethane (40 mL), the intermediate **5** (2.06 mmol) was added at room temperature. A solution of 3-bromoaniline (2.48 mmol) in dichloromethane (10 mL) was added dropwise. The mixture was stirred overnight at room temperature and monitored by TLC (petroleum ether/ethyl acetate 4:1). After the completion of the reaction, the mixture was washed with dilute hydrochloric acid, saturated sodium bicarbonate solution, and saturated brine, dried with anhydrous sodium sulfate, filtered, and concentrated. The residue was purified by silica gel chromatography (petroleum ether/ethyl acetate 4:1) to yield the target compound **6a.**

The synthesis of **6b~6x** are similar to that of **6a**. The structures of **6a~6x** were corroborated by ^1^HNMR, ^13^C NMR, and HRMS. All of the physical data and the HPLC chromatogram of **6j** are presented in the Supplementary Materials.

### 3.3. Biological Assay

*P. xylostella*, *O. furnacalis,* and *S. frugiperda* were reared continuously with fresh cabbage, corn leaves, and artificial feed, respectively, in our laboratory without insecticides. All target compounds were dissolved in 1 mL of dimethyl sulfoxide and diluted with an aqueous solution of 0.05% (*w/v*) Triton X-100 to prepare a 500 mg/L working solution. Other test concentrations were serially diluted with an aqueous solution of 0.05% (*w/v*) Triton X-100. The commercial insecticide tebufenozide was selected as the positive control. Water containing 0.05% (*w/v*) Triton X-100 with and without dimethyl sulfoxide were used as the negative and blank controls, respectively. The corrected mortality rate calculated as follows:

Corrected mortality rate (%) = *(T − C)* × 100/(100% − *C*)

where *T* is the mortality rate in the group of tested compounds and *C* is the mortality rate in the blank control group.

#### 3.3.1. Bioactivity Assay against *P. xylostella* and *O. furnacalis*

The insecticidal activities of the target compounds and the control insecticide tebufenozide against *P. xylostella* and *O. furnacalis* were conducted using the reported leaf-dipping method [22,23,24,25]. Cabbage or corn leaves were cut and dipped into the test solution for 10 s. After air drying, the treated leaf disks were placed in the Petri dish (10 cm in diameter). Each dry-treated leaf disk was infested with 15 second-instar larvae of *P. xylostella* or *O. furnacalis*. Subsequently, the experimental groups were cultivated in an incubator at 27 ± 1 °C, 70 ± 20% RH (relative humidity) and a 14:10 h light/dark photoperiod. The mortality rates were recorded at 96 h. The tests were replicated three times for each treatment (15 larvae per replicate).

#### 3.3.2. Bioactivity Assay against *S. frugiperda*

The biological activity against *S. frugiperda* was evaluated as previously described [19,26]. Artificial feed was added to the 24-well plate and 100 μL of the target compound solution was evenly dripped onto the surface of the feed after it had cooled and formed. After drying at room temperature, one second-instar larva was inserted into each well. The concentrations were repeated three times for each group (12 larvae per replicate). Each experiment was performed three times. The 24-well culture plates were stored in an incubator at 26 °C and 85% relative humidity at 16:8 h light/dark photoperiod. The mortality rates were confirmed after 144 h.

### 3.4. Molecular Docking

Molecular docking was performed by using the Surflex-Dock algorithm in Sybyl 7.3 software [27,28]. The crystal structures of the EcR/USP heterodimer (EcR/USP-LBD) from *Heliothis virescens* (PDB ID: 3IXP) and group I chitinase from *O. furnacalis* (PDB ID: 3WL1) obtained from the PDB protein data bank were used for the docking study. The docking results were visualized by Pymol (version 1.9.0) (http://www.pymol.org/ accessed on 10 February 2023) and Discovery Studio 2016 Client (for 2D interaction).

### 3.5. Ligand-Binding Assay on EcR/USP

Plasmids with concentrations of more than 500 ng/μL were extracted from the kit (Sigma-Aldrich, Shanghai, China). The [^3^H] PonA (tritiated PonA, 95 Ci·mmol^−1^, PerkinElmer Inc., Shelton CA, USA) radioactive binding test was performed on the in vitro translated *P. xylostella* EcR/USP (*Px*EcR and *Px*USP), as in a previously reported method [29].

### 3.6. Ligand-Inhibiting Assay on OfChtI

Chitinase *Of*ChtI was expressed and purified according to our published literature [18]. The inhibition assay was carried out following the standard strategy. 4-Methylumbelliferyl *β-D-N,N*′-diacetylchitobioside hydrate [4MU-(GlcNAc)_2_] (Sigma, Shanghai, China) was used as the substrate to calculate the inhibiting activity on *Of*ChtI based on fluorescence intensity, as in a previously reported method [11].

## 4. Conclusions

In conclusion, a total of 24 (23 novel) hexacyclic pyrazolamide derivatives were designed and synthesized through the strategy of reducing the heptacyclic ring and inserting small flexible linkers. The synthetic route was easy to perform, and the intermediate compound resulted in high atom economy. A bioassay indicated that some of the target compounds showed moderate to good insecticidal activity against *P. xylostella* and *S. frugiperda* larvae. Of these, compounds **6i**, **6j,** and **6n** exhibited promising insecticidal activity against *P. xylostella,* while **6i**, **6j,** and **6t** showed effective insecticidal activity against *S. frugiperda*. In particular, compound **6j** displayed the best activity, which was superior to that of lead **D-27**. SAR analysis revealed that inserting an additional methylene spacer between the substituted phenyl ring and the amide bond can improve the insecticidal activity. Furthermore, molecular docking and protein verification demonstrated that **6j** could act not only on EcR, but also on *Of*ChtI. The present results indicated that an appropriate increase in molecular flexibility could enhance the binding affinity of the compound with the EcR or *Of*ChtI, which would be very valuable for further structure optimization. This work provides a rational design strategy for the discovery of novel dual-target IGRs for green pest control.

## Figures and Tables

**Table 1:** Insecticidal activities (%) against lepidoptera pests of target compounds **6a~6x**.

Compounds	n	R	*P. xylostella* ^a^	*S. frugiperda* ^b^		*O. furnacalis* ^a^
			500 mg/L	200 mg/L	500 mg/L	200 mg/L
**6a**	0	3-Br	23.2 ± 1.5	nt	29.3 ± 1.5	4.2 ± 3.0
**6b**	0	4-Br	43.6 ± 1.9	11.6 ± 1.8	52.8 ± 3.9	6.5 ± 0.2
**6c**	0	3-Et	37.1 ± 1.0	8.4 ± 0.6	42.9 ± 1.8	24.3 ± 1.7
**6d**	0	4-Et	52.8 ± 2.0	35.0 ± 3.2	52.6 ± 1.8	2.2 ± 3.1
**6e**	0	4-OCH_3_	45.7 ± 3.4	17.4 ± 1.9	44.4 ± 2.0	11.2 ± 1.7
**6f**	0	4-*t*Bu	58.8 ± 3.5	30.3 ± 2.7	36.6 ± 2.7	7.8 ± 1.6
**6g**	1	3-F	14.3 ± 0.8	nt	50.0 ± 3.7	3.0 ± 4.3
**6h**	1	4-F	52.8 ± 2.0	28.4 ± 1.6	68.7 ± 2.9	4.6 ± 3.3
**6i**	1	3-Br	100 ± 0	35.0 ± 3.2	71.8 ± 1.3	12.5 ± 1.8
**6j**	1	4-Br	100 ± 0	40.3 ± 2.8	83.3 ± 0	33.5 ± 2.3
**6k**	1	3-OCF_3_	58.9 ± 0.8	4.0 ± 2.9	55.8 ± 1.8	10.1 ± 0.8
**6l**	1	4-OCF_3_	70.3 ± 3.5	10.8 ± 1.8	38.8 ± 1.7	0
**6m**	1	3-CH_3_	19.4 ± 2.0	nt	42.4 ± 2.3	16.2 ± 0.6
**6n**	1	4-CH_3_	100 ± 0	23.2 ± 1.5	44.2 ± 1.8	18.3 ± 2.5
**6o**	1	4-Et	59.4 ± 0.8	12.9 ± 1.3	60.0 ± 1.3	14.4 ± 1.6
**6p**	1	4-OCH_3_	62.9 ± 1.0	16.4 ± 1.6	56.1 ± 1.6	16.2 ± 0.6
**6q**	1	4-*t*Bu	35.8 ± 2.1	14.0 ± 1.0	43.9 ± 1.6	9.1 ± 0.7
**6r**	2	3-F	73.0 ± 3.1	10.8 ± 1.8	28.2 ± 1.3	0
**6s**	2	4-F	42.4 ± 2.3	8.2 ± 0.7	22.4 ± 2.0	0
**6t**	2	3-Br	83.1 ± 1.8	15.1 ± 1.4	69.1 ± 1.9	6.8 ± 0.2
**6u**	2	4-Br	30.2 ± 2.5	nt	45.1 ± 0.5	10.8 ± 1.8
**6v**	2	4-CH_3_	80.1 ± 1.3	33.3 ± 2.0	17.2 ± 0.7	20.9 ± 2.0
**6w**	2	4-Et	26.9 ± 1.5	nt	50.0 ± 3.7	8.1 ± 0.3
**6x**	2	4-OCH_3_	57.1 ± 1.8	36.1 ± 2.1	31.3 ± 2.9	4.9 ± 3.5
**D-27**	0	4-*t*Bu	65.8 ± 3.2	31.6 ± 1.2	29.3 ± 2.9	11.8 ± 2.9
**Tebufenozide**	/	/	100 ± 2.0	92.3 ± 0.5	100 ± 0	100 ± 0

^a^ Mortality was evaluated 96 h after treatment; ^b^ Mortality was evaluated 144 h after treatment; “nt”: not tested. The corrected mortality rates are represented as mean ± standard deviation.

## Data Availability

All data presented in this study are available in the article and the Supplementary Materials.

## Supplementary Materials

The following supporting information can be downloaded at: https://www.mdpi.com/article/10.3390/molecules28093741/s1, Table S1: The price and manufacturers of the starting reagents; Table S2: Comparison of the yields of hexacyclic and heptacyclic intermediates; Table S3: Comparison of the yields of hexacyclic and heptacyclic compounds; Table S4: Elution gradient and analysis conditions of HPLC; Figure S1: Predicted binding modes of **6f** and **D-27** with EcR and *Of*ChtI; Figure S2: Predicted binding modes of **6b**, **6j,** and **6u** with EcR and *Of*ChtI; Figure S3: the HPLC chromatogram of **6j;** physical and chemical properties, NMR, and HRMS spectra and data of **6a~6x**.

## Sample Availability

Samples of compounds **6a~6x** are available from the authors.
