# Supplementary material for: A Potential Lead for Insect Growth Regulator: Design, Synthesis, and Biological Activity Evaluation of Novel Hexacyclic Pyrazolamide Derivatives
Source: Molecules. 2023 Apr 26;28(9):3741. doi: 10.3390/molecules28093741 (PMC10179764; doi:10.3390/molecules28093741)

Supplementary Material

A Potential Lead for Insect Growth Regulator: Design, Synthesis, and Biological Activity Evaluation of Novel Hexacyclic Pyrazolamide Derivatives

Bingbo Guo, Biaobiao Jiang, Chunying Wang, Xiaoyu Jin, Liuyang Wang, Zhaokai Yang, Shihui Luo, Qing Yang, Li Zhang and Xinling Yang

Content

Table S1. The price and manufacturer of the starting reagents……………………………...…2

Table S2. Comparison of the yields of hexacyclic and heptacyclic intermediates…………2

Table S3. Comparison of the yields of hexacyclic and heptacyclic compounds………..…2

Figure S1. Predicted binding modes of **6f** and **D-27** with EcR and *Of*ChtI………..…....….3

Figure S2. Predicted binding modes of **6b**, **6j** and **6n** with EcR and *Of*ChtI…………….…3

Figure S3. The HPLC chromatogram of **6j**……...……..…………4

Table S4. Elution gradient and analysis conditions of HPLC………………………….……4

Physical and chemical properties, NMR and HRMS spectra data of **6a~6x**…......................5

^{1}H NMR and ^{13}C NMR spectra of target compounds 8

HRMS spectra of target compounds ... .32

Table S1. The price and manufacturer of the starting reagents (From Chemical Book)

Reagent.	Price	Manufacturer	Reagent.	Price	Manufacturer
**Cyclohexanone**	**¥ 49 /500mL**	**Macklin**	**Cyclohexanone**	**¥ 69 /1L**	**J&K Scientific**
**Cycloheptanone**	**¥ 1802/500mL**	**Macklin**	**Cycloheptanone**	**¥ 378 /0.1L**	**J&K Scientific**

Table S2. Comparison of the yields of hexacyclic and heptacyclic intermediates [1]

Intermediates (hexacyclic)[a]	yield (%)	Intermediates (heptacyclic)[b]	yield (%)
**Intermediate 3[a]**	75	**Intermediate 3[b]**	68
**Intermediate 4[a]**	58	**Intermediate 4[b]**	50

"a" represents the intermediate synthesized in this work; "b" represents the intermediate synthesized in our previous work [1]. Intermediate 3[b] represents ethyl 2-oxo-2-(2-oxocycloheptyl) acetate. Intermediate 4[b] represents ethyl 2-phenyl-2,4,5,6,7,8-hexahydrocyclohepta[c]pyrazol63-carboxylate

Table S3. Comparison of the yields of hexacyclic and heptacyclic compounds [1].

Compd[a].	R	yield (%)	Compd[b].	R	yield (%)
**6a**	**3-Br**	**48**	**D-08**	**3-Br**	**22**
**6b**	**4-Br**	**56**	**D-09**	**4-Br**	**49**
**6c**	**3-Et**	**59**	**D-23**	**3-Et**	**54**
**6d**	**4-Et**	**72**	**D-24**	**4-Et**	**64**
**6e**	**4-OCH_3**	**56**	**D-15**	**4-OCH_3**	**16**
**6f**	**4-tBu**	**76**	**D-27**	**4-tBu**	**74**

"a" represents the compound synthesized in this work; "b" represents the compound synthesized in our previous work [1].

[1] Jiang, B.; Jin, X.; Dong, Y.; Guo, B.; Cui, L.; Deng, X.; Zhang, L.; Yang, Q.; Li, Y.; Yang, X.; Smagghe, G. Design, Synthesis, and Biological Activity of Novel Heptacyclic Pyrazolamide Derivatives: A New Candidate of Dual-Target Insect Growth Regulators. *J Agric Food Chem.* **2020**, *68*, 6347-6354.

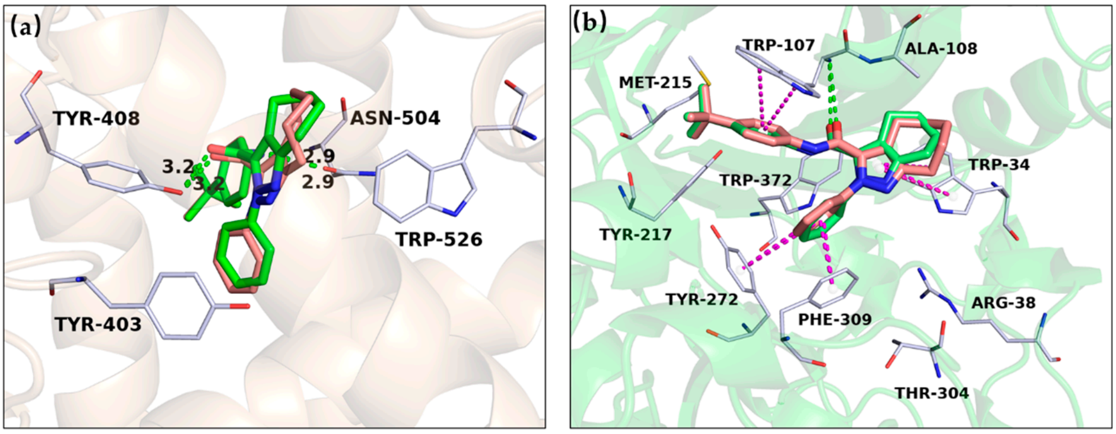

Figure S1. Predicted binding modes of **6f** and **D-27** with EcR and *Of*ChtI. (a) Superimposed conformations of the **6f** (green) and **D-27** (red) with EcR; (b) Superimposed conformations of the **6f** (green) and **D-27** (red) with *Of*ChtI.

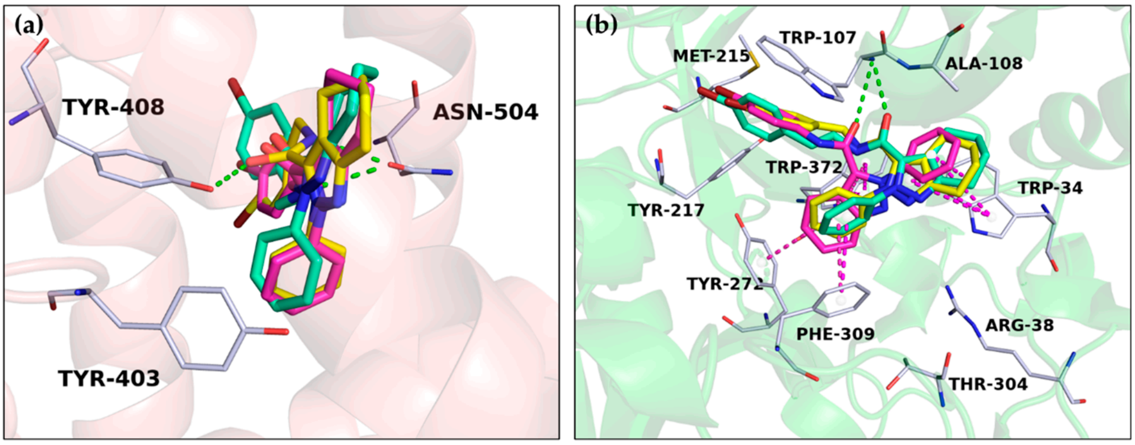

Figure S2. Predicted binding modes of **6b**, **6j**, and **6u** with EcR and *Of*ChtI. (a) Superimposed conformations of the **6b (**purple) **6j** (yellow) and **6u** (cyan) with EcR; (b) **6b** (purple) **6j** (yellow) and **6u** (cyan) with *Of*ChtI.

The HPLC chromatogram of 6j.

The samples were analyzed using an Agilent 1290 high-performance liquid chromatography system (Agilent Technologies, Santa Clara, CA, USA). Analysis conditions are listed in **Table S4**.

Table S4. Elution gradient and analysis conditions of HPLC.

Time（min）	Flow（mL/ min）	A%	B%
0	0.3	90.0	10.0
1.0	0.3	90.0	10.0
2.5	0.3	10.0	90.0
4.0	0.3	10.0	90.0
4.1	0.3	90.0	10.0
5.0	0.3	90.0	10.0

A: Acetonitrile

B: 0.2% formic acid aqueous solution

Column: Agilent Poroshell 120 EC-C18 (2.1 mm × 100 mm, particle size 1.9 μm)

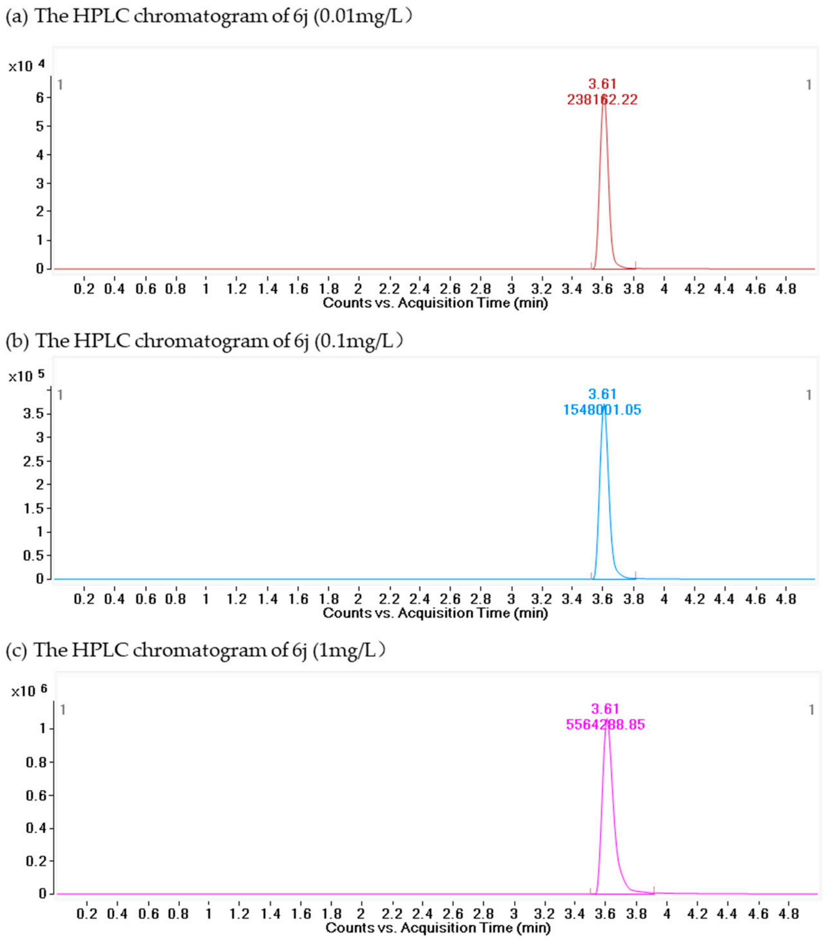

Figure S3. The HPLC chromatogram of **6j**. (a) The HPLC chromatogram of **6j** (0.01 mg/L); (b) The HPLC chromatogram of **6j** (0.1 mg/L); (c) The HPLC chromatogram of **6j** (1 mg/L).

Physical and chemical properties, NMR and HRMS spectra data of 6a~6x

(N-(3-bromophenyl)-2-phenyl-4,5,6,7-tetrahydro-2H-indazole-5-carboxamide) **(6a)**: white solid, yield 48%, m.p. 168-169℃. ^{1}H NMR (500 MHz, DMSO-*d*$_6$) *δ* 7.98 (t, *J* = 9.0 Hz, 1H), 7.77–7.28 (m, 9H), 2.73 (t, *J* = 6.1 Hz, 2H), 2.63 (t, *J* = 6.1 Hz, 2H), 1.81–1.67 (m, 4H). ^{13}C NMR (126 MHz, DMSO-*d*$_6$) *δ* 161.11, 149.75, 141.24, 130.34, 128.83, 128.29, 128.08, 127.86, 125.98, 124.96, 123.19, 119.70, 110.02, 23.26, 22.94, 22.92, 22.33. HRMS (ESI) calcd for $C_{20}H_{18}BrN_3O$ $(M+H)^+$ 396.0706, found 396.0709.

N-(4-bromophenyl)-2-phenyl-4,5,6,7-tetrahydro-2H-indazole-5-carboxamide **(6b):** white solid, yield 56%, m.p. 226-227℃. ^{1}H NMR (500 MHz, $CDCl_3$) *δ* 7.43–7.26 (m, 7H), 7.20–7.08 (m, 3H), 2.75 (t, *J* = 5.9 Hz, 2H), 2.68 (t, *J* = 6.1 Hz, 2H), 1.83–1.71 (m, 4H). ^{13}C NMR (126 MHz, $CDCl_3$) *δ* 157.02, 149.58, 138.86, 135.22, 131.66, 131.01, 128.29, 127.39, 124.00, 120.29, 120.06, 116.37, 22.26, 21.93, 21.82, 20.79. HRMS (ESI) calcd for $C_{20}H_{18}BrN_3O$ $(M+H)^+$ 396.0706, found 396.0706.

N-(3-ethylphenyl)-2-phenyl-4,5,6,7-tetrahydro-2H-indazole-5-carboxamide Chemical Formula **(6c)**: white solid, yield 59%, m.p. 154-155℃. ^{1}H NMR (500 MHz, DMSO-*d*$_6$) *δ* 10.46 (s, 1H), 7.55–7.39 (m, 6H), 7.36–7.19 (m, 2H), 6.96 (d, *J* = 7.6 Hz, 1H), 2.69 (t, *J* = 5.1 Hz, 4H), 2.57 (q, *J* = 7.6 Hz, 2H), 1.84-1.71 (m, 4H), 1.16 (t, *J* = 7.6 Hz, 3H). ^{13}C NMR (126 MHz, DMSO-*d*$_6$) *δ* 159.56, 149.66, 144.88, 140.35, 139.03, 134.85, 129.49, 129.17, 127.40, 124.03, 123.27, 119.50, 118.79, 117.62, 28.70, 23.32, 23.15, 23.00, 20.66, 15.95. HRMS (ESI) calcd for $C_{22}H_{23}N_3O$ $(M+H)^+$ 346.1914, found 346.1916.

N-(4-ethylphenyl)-2-phenyl-4,5,6,7-tetrahydro-2H-indazole-5-carboxamide **(6d)**: white solid, yield 72%, m.p. 189-190℃.^{1}H NMR (500 MHz, DMSO-*d*$_6$) *δ* 10.45 (s, 1H), 7.55–7.41 (m, 6H), 7.34–7.27 (m, 1H), 7.16 (d, *J* = 8.3 Hz, 2H), 2.71–2.63(m, 4H), 2.55 (q, *J* = 7.6 Hz, 2H), 1.85–1.70 (m, 4H), 1.15 (t, *J* = 6.3 Hz, 3H). ^{13}C NMR (126 MHz, DMSO-*d*$_6$) *δ* 159.44, 149.66, 140.35, 140.09, 136.71, 134.89, 129.47, 128.46, 127.36, 123.19, 120.25, 118.70, 28.13, 23.32, 23.15, 23.01, 20.63, 16.24. HRMS (ESI) calcd for $C_{22}H_{23}N_3O$ $(M+H)^+$ 346.1914, found 346.1915.

N-(4-methoxyphenyl)-2-phenyl-4,5,6,7-tetrahydro-2H-indazole-5-carboxamide **(6e)**: white solid, yield 56%, m.p. 210-221℃. ^{1}H NMR (500 MHz, DMSO-*d*$_6$) *δ* 10.38 (s, 1H), 7.57–7.40 (m, 6H), 7.36–7.27 (m, 1H), 6.90 (d, *J* = 8.8 Hz, 2H), 3.73 (d, *J* = 3.1 Hz, 3H), 2.73–2.62(m, 4H), 1.86–1.68 (m, 4H). ^{13}C NMR (126 MHz, DMSO-*d*$_6$) *δ* 159.20, 156.31, 149.65, 140.37, 134.93, 132.12, 129.47, 127.34, 123.17, 121.72, 118.61, 114.41, 55.71, 23.33, 23.16, 23.02, 20.64. HRMS (ESI) calcd for $C_{21}H_{21}N_3O_2$ $(M+H)^+$ 348.1707, found 348.1708.

N-(4-(tert-butyl)phenyl)-2-phenyl-4,5,6,7-tetrahydro-2H-indazole-5-carboxamide **(6f)**: white solid, yield 76%, m.p. 155-156℃. ^{1}H NMR (500 MHz, DMSO-*d*$_6$) *δ* 10.45 (s, 1H), 7.54 (d, *J* = 8.6 Hz, 2H), 7.45–7.41 (m, 4H), 7.36–7.28 (m, 3H), 2.68 (q, *J* = 6.2 Hz, 4H), 1.85–1.71 (m, 4H), 1.26 (s, 9H). ^{13}C NMR (126 MHz, DMSO-*d*$_6$) *δ* 159.46, 149.66, 146.97, 140.34, 136.46, 134.89, 129.48, 127.35, 125.88, 123.16, 119.92, 118.69, 34.54, 31.63, 23.33, 23.16, 23.02, 20.61. HRMS (ESI) calcd for $C_{24}H_{27}N_3O$ $(M+H)^+$ 374.2227, found 374.2224.

N-(3-fluorobenzyl)-2-phenyl-4,5,6,7-tetrahydro-2H-indazole-5-carboxamide **(6g)**: white solid, yield 64%, m.p. 214-215℃. ^{1}H NMR (500 MHz, DMSO-*d*$_6$) *δ* 9.02 (t, *J* = 6.0 Hz, 1H), 7.42–7.28 (m, 6H), 7.15–7.02 (m, 3H), 4.41 (d, *J* = 6.0 Hz, 2H), 2.68–2.57 (m, 4H)., 1.82–1.67 (m, 4H). ^{13}C NMR (126 MHz, DMSO-*d*$_6$) *δ* 162.64 (d, *J* = 243.2 Hz), 161.32, 149.56, 142.41 (d, *J* = 7.2 Hz), 140.41, 134.56, 130.73 (d, *J* = 8.7 Hz), 129.25, 127.33, 123.84 (d, *J* = 2.8 Hz), 123.40, 118.22, 114.52 (d, *J* = 21.7 Hz), 114.15 (d, *J* = 20.9 Hz), 42.52, 23.30, 23.14, 23.04, 20.70. HRMS (ESI) calcd for $C_{21}H_{20}FN_3O$ $(M+H)^+$ 350.1663, found 350.1661.

N-(4-fluorobenzyl)-2-phenyl-4,5,6,7-tetrahydro-2H-indazole-5-carboxamide **(6h)**: white solid, yield 68%, m.p. 189-190℃.^{1}H NMR (500 MHz, DMSO-*d*$_6$) *δ* 8.99 (t, *J* = 6.0 Hz, 1H), 7.40–7.27 (m, 7H), 7.19–7.13 (m, 2H), 4.37 (d, *J* = 6.0 Hz, 2H), 2.67–2.58 (m, 4H), 1.81–1.66 (m, 4H). ^{13}C NMR (126 MHz, DMSO-*d*$_6$) *δ* 161.30, 149.53, 140.48, 137.66, 134.48, 132.91, 129.54, 129.50, 129.32, 128.18, 127.37, 123.59, 122.90, 118.34, 43.42, 23.32, 23.15, 23.07, 20.79. HRMS (ESI) calcd for $C_{21}H_{20}FN_3O$ $(M+H)^+$ 350.1663, found 350.1665.

N-(3-bromobenzyl)-2-phenyl-4,5,6,7-tetrahydro-2H-indazole-5-carboxamide **(6i):** white solid, yield 64%, m.p. 217-218℃. ^{1}H NMR (500 MHz, DMSO-*d*$_6$) *δ* 9.02 (t, *J* = 6.1 Hz, 1H), 7.52–7.24

(m, 9H), 4.39 (d, *J* = 6.1 Hz, 2H), 2.68–2.59(m, 4H), 1.82–1.68 (m, 4H). ^{13}C NMR (126 MHz, DMSO-*d*$_6$) *δ* 161.30, 149.58, 142.30, 140.37, 134.48, 130.99, 130.57, 130.24, 129.29, 127.33, 126.94, 123.32, 122.07, 118.27, 42.42, 23.29, 23.14, 23.03, 20.73. HRMS (ESI) calcd for $C_{21}H_{20}BrN_3O$ $(M+H)^+$ 410.0863, found 410.0859.

N-(4-bromobenzyl)-2-phenyl-4,5,6,7-tetrahydro-2H-indazole-5-carboxamide**(6j):** white solid, yield 65%, m.p. 220-221℃. ^{1}H NMR (500 MHz, DMSO-*d*$_6$) *δ* 8.93 (t, *J* = 6.0 Hz, 1H), 7.46 (d, *J* = 8.3 Hz, 2H), 7.35–7.21 (m, 5H), 7.15 (d, *J* = 8.3 Hz, 2H), 4.28 (d, *J* = 6.0 Hz, 2H), 2.62–2.49 (m, 4H), 1.75–1.60 (m, 4H). ^{13}C NMR (151 MHz, DMSO-*d*$_6$) *δ* 161.24, 149.54, 140.40, 138.92, 134.54, 131.65, 130.13, 129.29, 127.32, 123.38, 120.42, 118.21, 42.39, 23.29, 23.14, 23.04, 20.69. HRMS (ESI) calcd for $C_{21}H_{20}BrN_3O$ $(M+H)^+$ 410.0863, found 410.0864.

2-phenyl-N-(3-(trifluoromethoxy)benzyl)-4,5,6,7-tetrahydro-2H-indazole-5-carboxamide **(6k):** white solid, yield 60%, m.p. 198-199℃. ^{1}H NMR (500 MHz, DMSO-*d*$_6$) *δ* 9.05 (t, *J* = 6.1 Hz, 1H), 7.48 (t, *J* = 7.8 Hz, 1H), 7.39–7.24 (m, 8H), 4.44 (d, *J* = 6.1 Hz, 2H), 2.68–2.59 (m, 4H), 1.83–1.67 (m, 4H). ^{13}C NMR (126 MHz, DMSO-*d*$_6$) *δ* 161.30, 149.54, 146.60, 140.44, 134.41, 131.54, 129.96, 129.40, 129.29, 127.93, 127.33, 123.48, 121.11, 118.35, 37.52, 23.30, 23.13, 23.04, 20.67. HRMS (ESI) calcd for $C_{22}H_{20}F_3N_3O_2$ $(M+H)^+$ 416.1580, found 416.1583.

2-phenyl-N-(4-(trifluoromethoxy)benzyl)-4,5,6,7-tetrahydro-2H-indazole-5-carboxamide **(6l):** white solid, yield 72%, m.p. 214-215℃.^{1}H NMR (500 MHz, DMSO-*d*$_6$) *δ* 8.98 (t, *J* = 5.9 Hz, 1H), 7.44 – 7.29 (m, 9H), 4.47 (d, *J* = 5.9 Hz, 2H), 2.69–2.60 (m, 4H), 1.81–1.69 (m, 4H). ^{13}C NMR (126 MHz, DMSO-*d*$_6$) *δ* 161.27, 149.55, 147.71, 140.39, 138.96, 134.54, 129.72, 129.24, 127.28, 123.37, 121.44, 118.21, 42.32, 23.29, 23.14, 23.04, 20.69. HRMS (ESI) calcd for $C_{22}H_{20}F_3N_3O_2$ $(M+H)^+$ 416.1580, found 416.1583.

N-(3-methylbenzyl)-2-phenyl-4,5,6,7-tetrahydro-2H-indazole-5-carboxamide **(6m):** white solid, yield 77%, m.p. 200-201℃. ^{1}H NMR (500 MHz, DMSO-*d*$_6$) *δ* 8.88 (t, *J* = 5.8 Hz, 1H), 7.40–7.28 (m, 5H), 7.21–7.11 (m, 4H), 4.37 (d, *J* = 5.8 Hz, 2H), 2.67–2.58 (m, 4H), 2.26 (s, 3H), 1.81–1.67 (m, 4H). ^{13}C NMR (126 MHz, DMSO-*d*$_6$) *δ* 161.20, 149.52, 140.44, 139.33, 137.80, 134.71, 129.26, 128.68, 128.43, 127.98, 127.24, 124.91, 123.30, 118.14, 42.91, 23.31, 23.16, 23.05, 21.51, 20.72. HRMS (ESI) calcd for $C_{22}H_{23}N_3O$ $(M+H)^+$ 346.1914, found 346.1915.

N-(4-methylbenzyl)-2-phenyl-4,5,6,7-tetrahydro-2H-indazole-5-carboxamide (**6n):** white solid, yield 72%, m.p. 201-202℃. ^{1}H NMR (500 MHz, DMSO-*d*$_6$) *δ* 8.94 (t, *J* = 6.0 Hz, 1H), 7.40–7.28 (m, 5H), 7.18–7.10 (m, 4H), 4.34 (d, *J* = 6.0 Hz, 2H), 2.68–2.57 (m, 4H), 2.29 (s, 3H), 1.81–1.67 (m, 4H). ^{13}C NMR (126 MHz, DMSO-*d*$_6$) *δ* 161.15, 149.50, 140.43, 136.44, 136.40, 134.73, 129.30, 129.27, 127.84, 127.23, 123.29, 118.10, 42.70, 23.30, 23.16, 23.05, 21.16, 20.69. HRMS (ESI) calcd for $C_{22}H_{23}N_3O$ $(M+H)^+$ 346.1914, found 346.1913.

N-(4-ethylbenzyl)-2-phenyl-4,5,6,7-tetrahydro-2H-indazole-5-carboxamide **(6o):** white solid, yield 57%, m.p. 177-178℃.^{1}H NMR (500 MHz, DMSO-*d*$_6$) *δ* 8.95 (t, *J* = 6.0 Hz, 1H), 7.41–7.27 (m, 5H), 7.20–7.12 (m, 4H), 4.35 (d, *J* = 6.0 Hz, 2H), 2.68–2.55 (m, 6H), 1.81–1.66 (m, 4H), 1.17 (t, *J* = 7.6 Hz, 3H). ^{13}C NMR (126 MHz, DMSO-*d*$_6$) *δ* 161.17, 149.50, 142.87, 140.44, 136.64, 134.74, 129.26, 128.13, 127.89, 127.20, 123.28, 118.09, 42.74, 28.32, 23.31, 23.16, 23.05, 20.70, 16.22. HRMS (ESI) calcd for $C_{23}H_{25}N_3O$ $(M+H)^+$ 360.2070, found 360.2071.

N-(4-methoxybenzyl)-2-phenyl-4,5,6,7-tetrahydro-2H-indazole-5-carboxamide **(6p)**: white solid, yield 86%, m.p. 181-182℃ , ^{1}H NMR (300 MHz, DMSO) *δ* 8.94 (t, *J* = 6.0 Hz, 1H), 7.43–7.17 (m, 7H), 6.91 (d, *J* = 8.6 Hz, 2H), 4.34 (d, *J* = 6.0 Hz, 2H), 3.76 (s, 3H), 2.70–2.57(m, 4H), 1.85–1.66 (m, 4H). ^{13}C NMR (151 MHz, DMSO-d$_6$) *δ* 161.08, 158.74, 149.49, 140.40, 134.73, 131.38, 129.27, 129.20, 127.20, 123.22, 118.07, 114.15, 55.55, 42.40, 23.30, 23.15, 23.04, 20.67. HRMS (ESI) calcd for $C_{22}H_{23}N_3O_2$ $(M+H)^+$ 362.1863, found 362.1864.

N-(4-(tert-butyl)benzyl)-2-phenyl-4,5,6,7-tetrahydro-2H-indazole-5-carboxamide **(6q):** white solid, yield 84%, m.p. 162-163℃.^{1}H NMR (300 MHz, DMSO-*d*$_6$) *δ* 8.95 (t, *J* = 5.9 Hz, 1H), 7.48–7.26 (m, 7H), 7.21 (d, *J* = 8.2 Hz, 2H), 4.37 (d, *J* = 6.0 Hz, 2H,), 2.75-2.56 (m, 4H), 1.88-1.65 (m, 4H), 1.30 (s, 9H). ^{13}C NMR (151 MHz, DMSO-*d*$_6$) *δ* 161.18, 149.75, 149.49, 140.41, 136.33, 134.72, 129.25, 127.59, 127.17, 125.49, 123.25, 118.08, 42.66, 34.67, 31.66, 23.30, 23.16, 23.05, 20.71. HRMS (ESI) calcd for $C_{25}H_{29}N_3O$ $(M+H)^+$ 388.2383, found 388.2382.

N-(3-fluorophenethyl)-2-phenyl-4,5,6,7-tetrahydro-2H-indazole-5-carboxamide **(6r):** white solid, yield 68%, m.p. 165-166℃. ^{1}H NMR (500 MHz, DMSO-*d*$_6$) *δ* 8.47 (t, *J* = 5.5 Hz, 1H), 7.44–

7.25 (m, 6H), 7.11–7.03 (m, 3H), 3.50 (dd, *J* = 12.7, 6.7 Hz, 2H), 2.84 (t, *J* = 6.9 Hz, 2H), 2.62 (t, *J* = 6.2 Hz, 2H), 2.47 (t, *J* = 6.1 Hz, 2H), 1.80–1.64 (m, 4H). ^{13}C NMR (126 MHz, DMSO-d_6) δ 162.68 (d, *J* = 243.0 Hz), 161.04, 149.42, 142.64 (d, *J* = 7.2 Hz), 140.43, 134.67, 130.55 (d, *J* = 8.1 Hz), 129.25, 127.13, 125.40, 123.09, 118.08, 115.92 (d, *J* = 20.5 Hz), 113.40 (d, *J* = 21.1 Hz), 40.26, 34.63, 23.27, 23.15, 23.02, 20.59. HRMS (ESI) calcd for $C_{22}H_{22}FN_3O$ $(M+H)^+$ 364.1820, found 364.1821.

N-(4-fluorophenethyl)-2-phenyl-4,5,6,7-tetrahydro-2H-indazole-5-carboxamide **(6s):** white solid, yield 83%, m.p. 203-204℃. ^{1}H NMR (500 MHz, DMSO-d_6) δ 8.45 (t, *J* = 5.5 Hz, 1H), 7.43–7.22 (m, 7H), 7.12 (t, *J* = 8.8 Hz, 2H), 3.46 (dd, *J* = 12.8, 6.8 Hz, 2H), 2.79 (t, *J* = 6.9 Hz, 2H), 2.62 (t, *J* = 6.1 Hz, 2H), 2.48 (t, *J* = 6.1 Hz, 2H), 1.82–1.62 (m, 4H). ^{13}C NMR (126 MHz, DMSO-d_6) δ 161.36 (d, *J* = 241.7 Hz), 161.00, 149.42, 140.44, 135.75 (d, *J* = 2.7 Hz), 134.70, 131.03, 130.97, 129.23, 127.13, 123.13, 118.07, 115.48, 115.31, 40.63, 34.17, 23.27, 23.15, 23.01, 20.59. HRMS (ESI) calcd for $C_{22}H_{22}FN_3O$ $(M+H)^+$ 364.1820, found 364.1821.

N-(3-bromophenethyl)-2-phenyl-4,5,6,7-tetrahydro-2H-indazole-5-carboxamide **(6t):** white solid, yield 61%, m.p. 185-186℃. ^{1}H NMR (500 MHz, DMSO-d_6) δ 8.46 (t, *J* = 5.5 Hz, 1H), 7.47–7.35 (m, 4H), 7.32–7.23 (m, 5H), 3.48 (q, *J* = 6.4 Hz, 2H), 2.81 (t, *J* = 6.7 Hz, 2H), 2.61 (t, *J* = 6.1 Hz, 2H), 2.45 (t, *J* = 6.0 Hz, 2H), 1.78–1.65 (m, 4H). ^{13}C NMR (126 MHz, DMSO-d_6) δ 161.04, 149.41, 142.65, 140.42, 134.66, 132.12, 130.88, 129.52, 129.27, 128.41, 127.12, 123.07, 122.06, 118.08, 40.39, 34.46, 23.28, 23.16, 23.08, 20.64. HRMS (ESI) calcd for $C_{22}H_{22}BrN_3O$ $(M+H)^+$ 424.1019, found 424.1020.

N-(4-bromophenethyl)-2-phenyl-4,5,6,7-tetrahydro-2H-indazole-5-carboxamide **(6u):** white solid; yield 77%, m.p. 209-210℃. ^{1}H NMR (500 MHz, DMSO-d_6) δ 8.43 (t, *J* = 5.6 Hz, 1H), 7.51–7.36 (m, 4H), 7.32–7.17 (m, 5H), 3.47 (q, *J* = 6.8 Hz, 2H), 2.78 (t, *J* = 6.9 Hz, 2H), 2.61 (t, *J* = 6.2 Hz, 2H), 2.45 (t, *J* = 6.1 Hz, 2H), 1.78–1.63 (m, 4H).^{13}C NMR (126 MHz, DMSO-d_6) δ 160.99, 149.42, 140.43, 139.11, 134.67, 131.56, 129.24, 127.12, 123.12, 119.74, 118.09, 34.37, 23.28, 23.15, 23.02, 20.58. ESI-HRMS (m/z) found: 424.1019 (calcd. for $C_{22}H_{22}BrN_3O$ $[M+H]^+$: 424.1019). HRMS (ESI) calcd for $C_{22}H_{22}BrN_3O$ $(M+H)^+$ 424.1019, found 424.1019.

N-(4-methylphenethyl)-2-phenyl-4,5,6,7-tetrahydro-2H-indazole-5-carboxamide (**6v**): white solid, yield 76%, m.p. 184-185℃. ^{1}H NMR (500 MHz, DMSO-d_6) δ 8.44 (t, *J* = 5.6 Hz, 1H), 7.43–7.07 (m, 9H), 3.43 (dd, *J* = 13.0, 7.0 Hz, 2H), 2.75 (t, *J* = 7.2 Hz, 2H), 2.62 (t, *J* = 6.2 Hz, 2H), 2.49–2.43 (m, 2H), 2.27 (s, 3H), 1.79–1.62 (m, 4H). ^{13}C NMR (126 MHz, DMSO-d_6) δ 160.97, 149.41, 140.44, 136.47, 135.52, 134.74, 129.33, 129.25, 129.06, 127.12, 123.12, 118.08, 40.77, 34.70, 23.28, 23.16, 23.03, 21.12, 20.59. HRMS (ESI) calcd for $C_{23}H_{25}N_3O$ $(M+H)^+$ 360.2070, found 360.2069.

N-(4-ethylphenethyl)-2-phenyl-4,5,6,7-tetrahydro-2H-indazole-5-carboxamide (**6w**): white solid, yield 73%, m.p. 130-131℃. ^{1}H NMR (500 MHz, DMSO-d_6) δ 8.45 (t, *J* = 5.6 Hz, 1H), 7.41–7.36 (m, 2H), 7.33–7.28 (m, 3H), 7.16–7.09 (m, 4H), 3.44 (dd, *J* = 13.0, 7.0 Hz, 2H), 2.76 (t, *J* = 7.2 Hz, 2H), 2.64–2.54 (m, 4H), 2.45 (t, *J* = 6.2 Hz, 2H), 1.78–1.63 (m, 4H), 1.16 (t, *J* = 7.6 Hz, 3H). ^{13}C NMR (126 MHz, DMSO-d_6) δ 160.99, 149.41, 141.95, 140.45, 136.73, 134.76, 129.26, 129.09, 128.14, 127.12, 123.13, 118.07, 40.72, 34.67, 28.28, 23.28, 23.16, 23.03, 20.56, 16.18. HRMS (ESI) calcd for $C_{24}H_{27}N_3O$ $(M+H)^+$ 374.2227, found 374.2229.

N-(4-methoxyphenethyl)-2-phenyl-4,5,6,7-tetrahydro-2H-indazole-5-carboxamide (**6x**): white solid, yield 90%, m.p. 187-188℃. ^{1}H NMR (300 MHz, DMSO-d_6) δ 8.43 (t, *J* = 5.7 Hz, 1H), 7.48–7.11 (m, 7H), 6.94–6.81 (m, 2H), 3.74 (s, 3H), 3.44 (dd, *J* = 12.8, 7.1 Hz, 2H), 2.75 (t, *J* = 7.1 Hz, 2H), 2.64 (t, *J* = 6.0 Hz, 2H), 2.51–2.47 (m, 2H), 1.84 – 1.54 (m, 4H). ^{13}C NMR (151 MHz, DMSO-d_6) δ 160.94, 158.19, 149.40, 140.43, 134.74, 131.43, 130.16, 129.24, 127.11, 123.11, 118.06, 114.17, 55.46, 40.90, 34.22, 23.28, 23.15, 23.02, 20.60. HRMS (ESI) calcd for $C_{23}H_{25}N_3O_2$ $(M+Na)^+$ 398.1839, found 398.1840.

^{1}H NMR and ^{13}C NMR spectra of target compounds

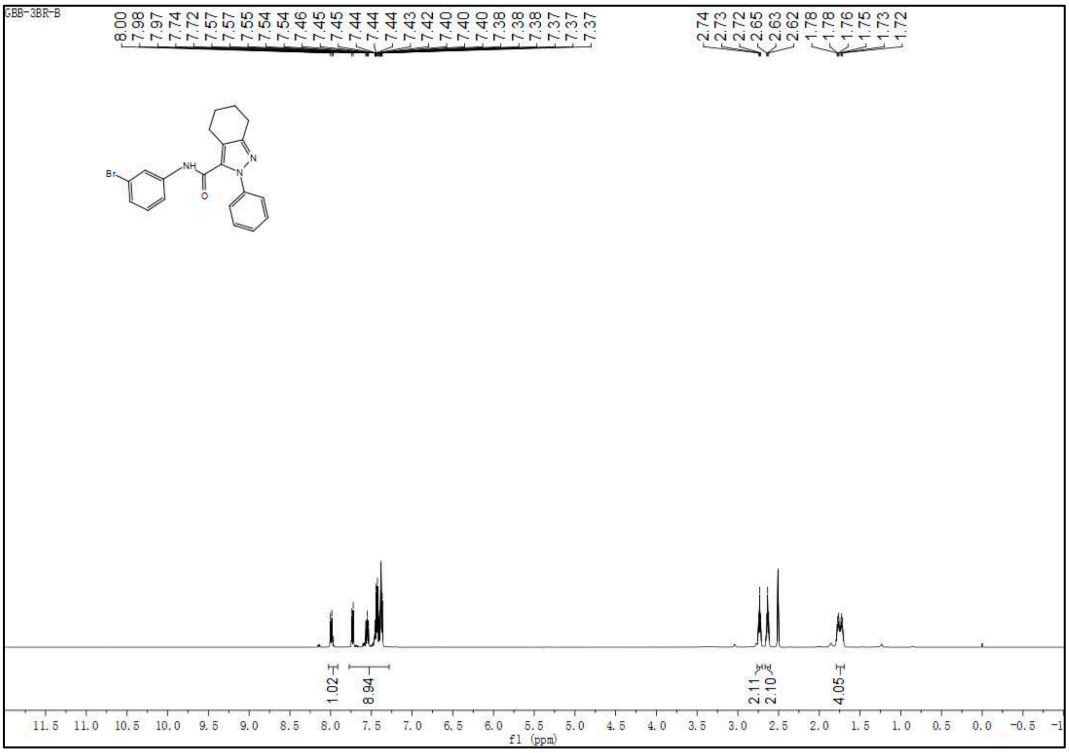

^{1}H NMR spectra of compound **6a**

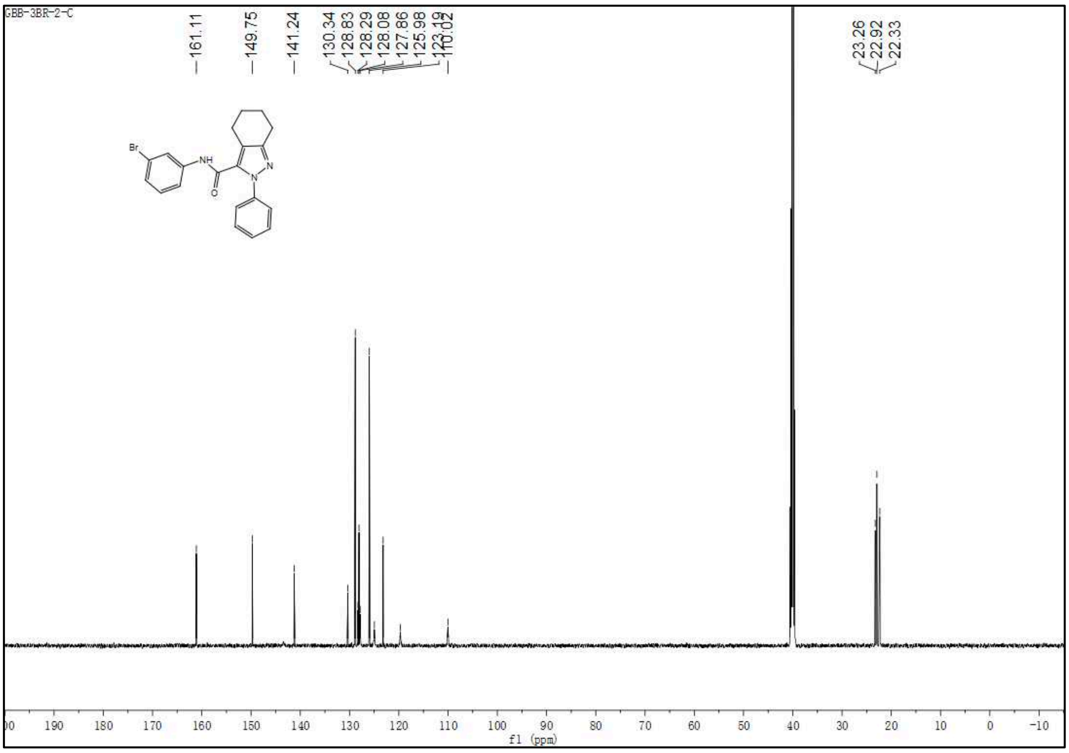

^{13}C NMR spectra of compound **6a**

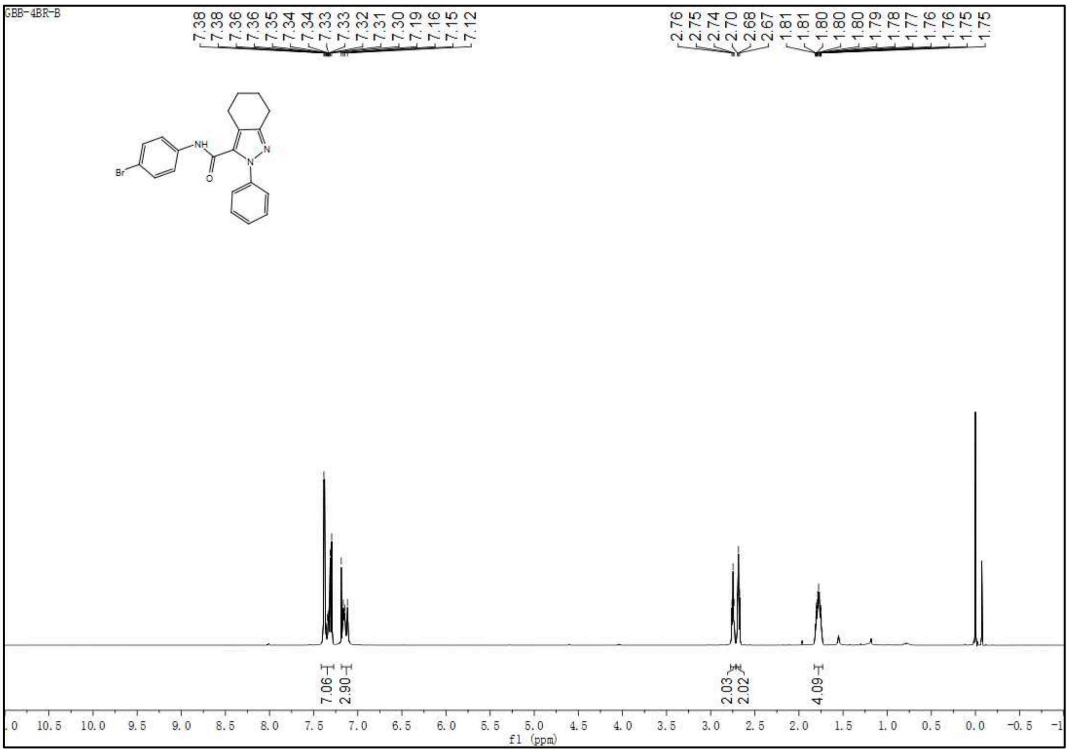

^{1}H NMR spectra of compound **6b**

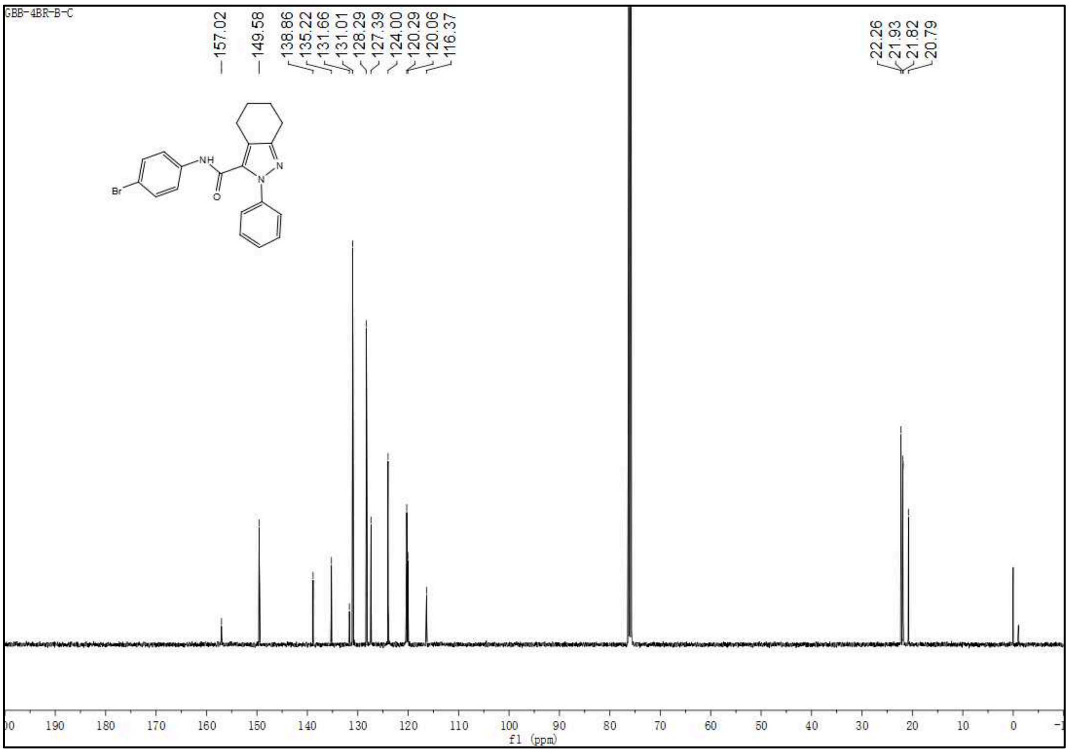

^{13}C NMR spectra of compound **6b**

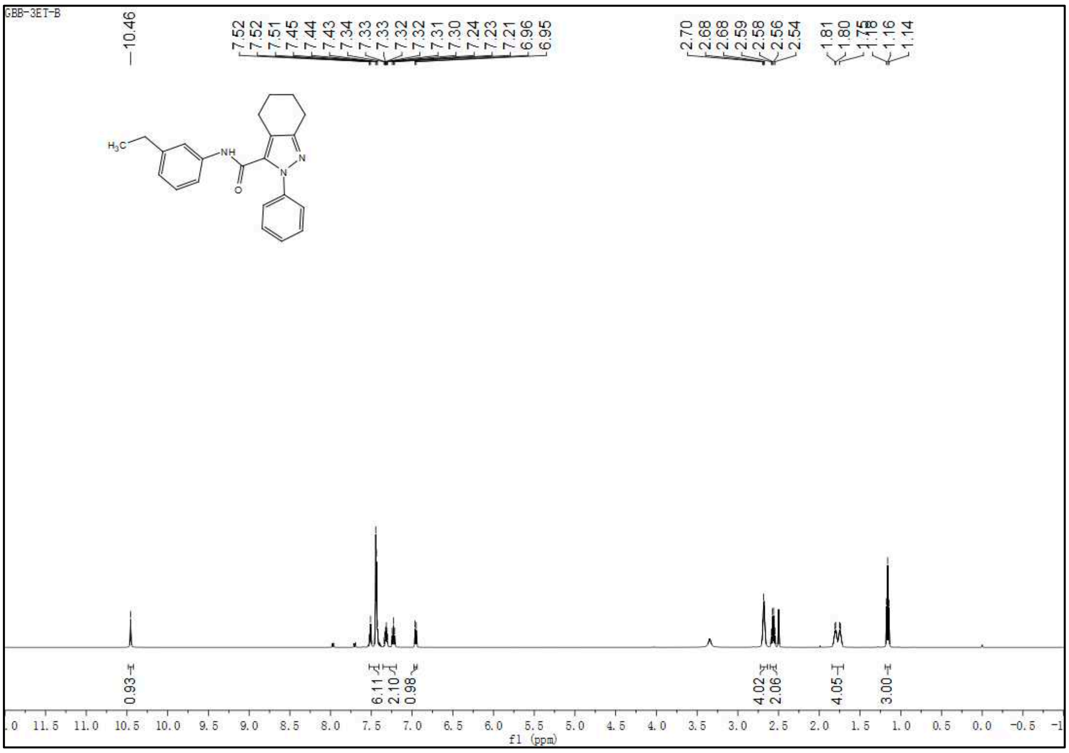

^{1}H NMR spectra of compound **6c**

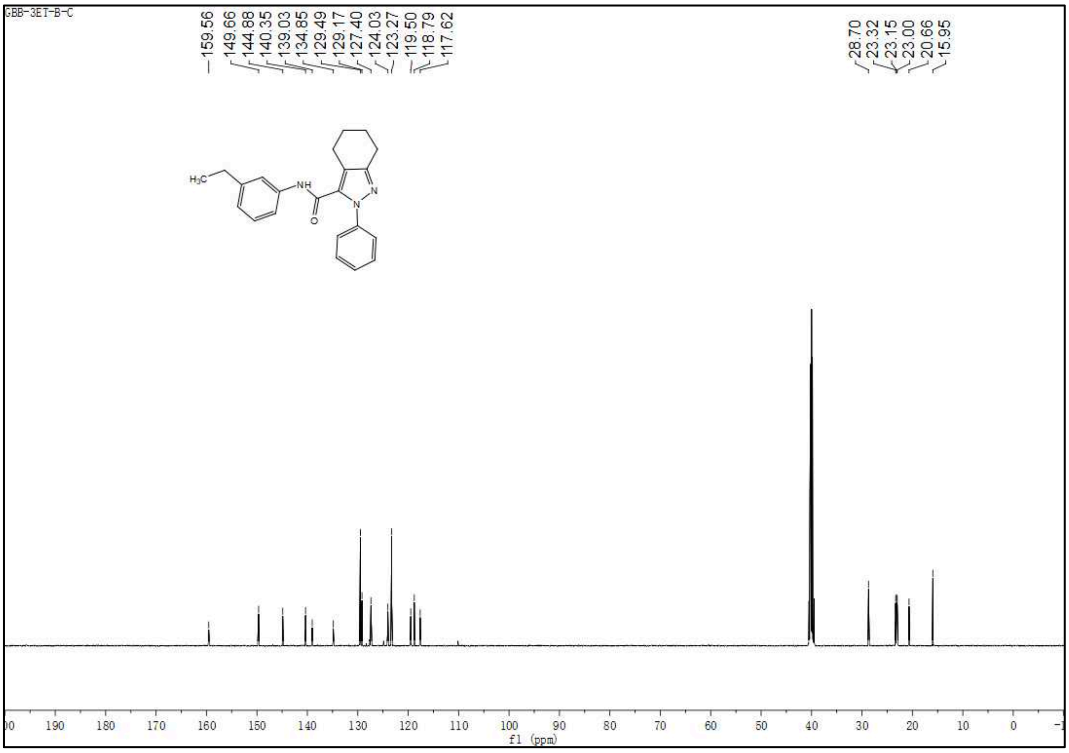

^{13}C NMR spectra of compound **6c**

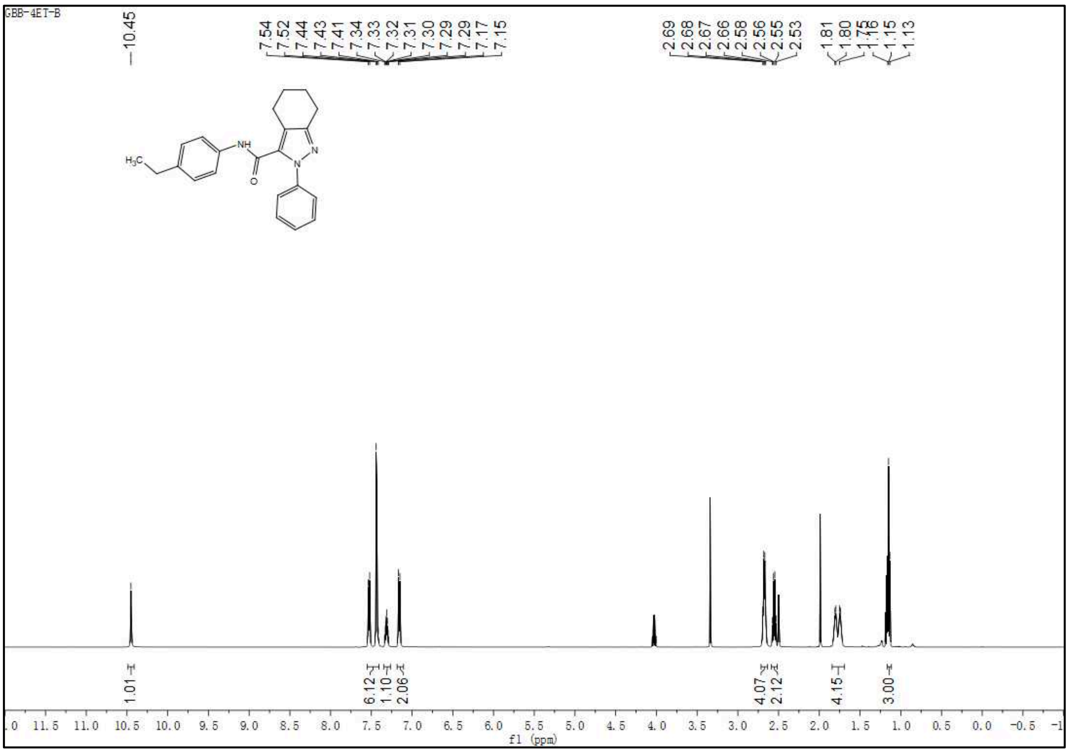

^{1}H NMR spectra of compound **6d**

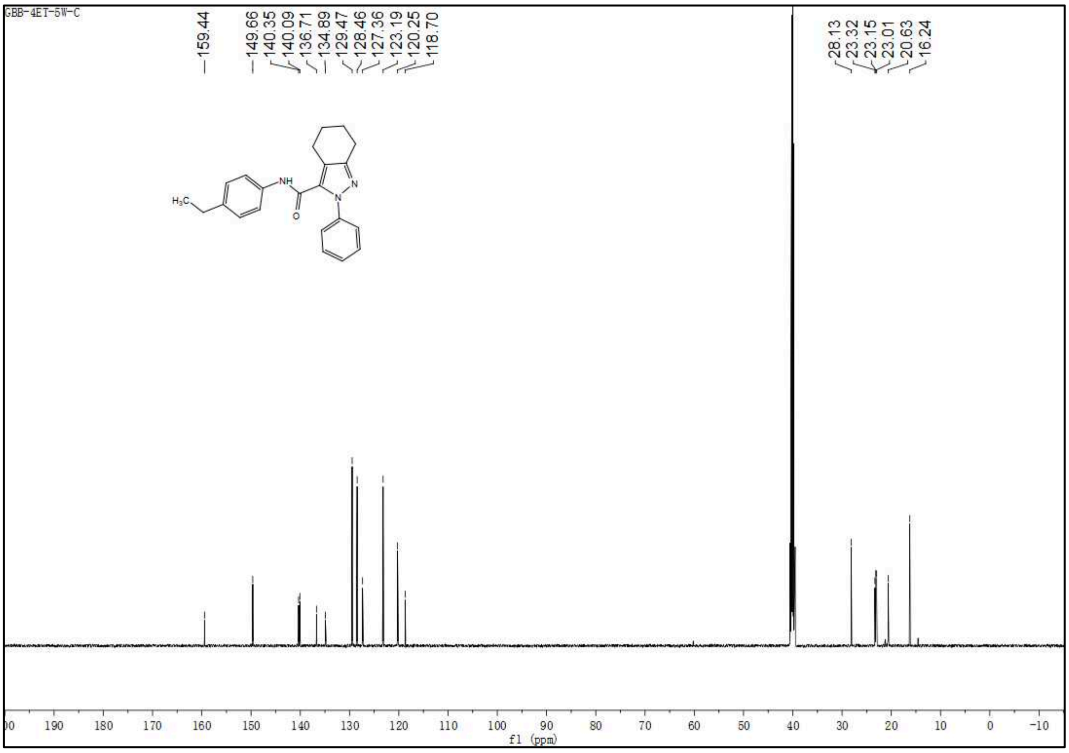

^{13}C NMR spectra of compound **6d**

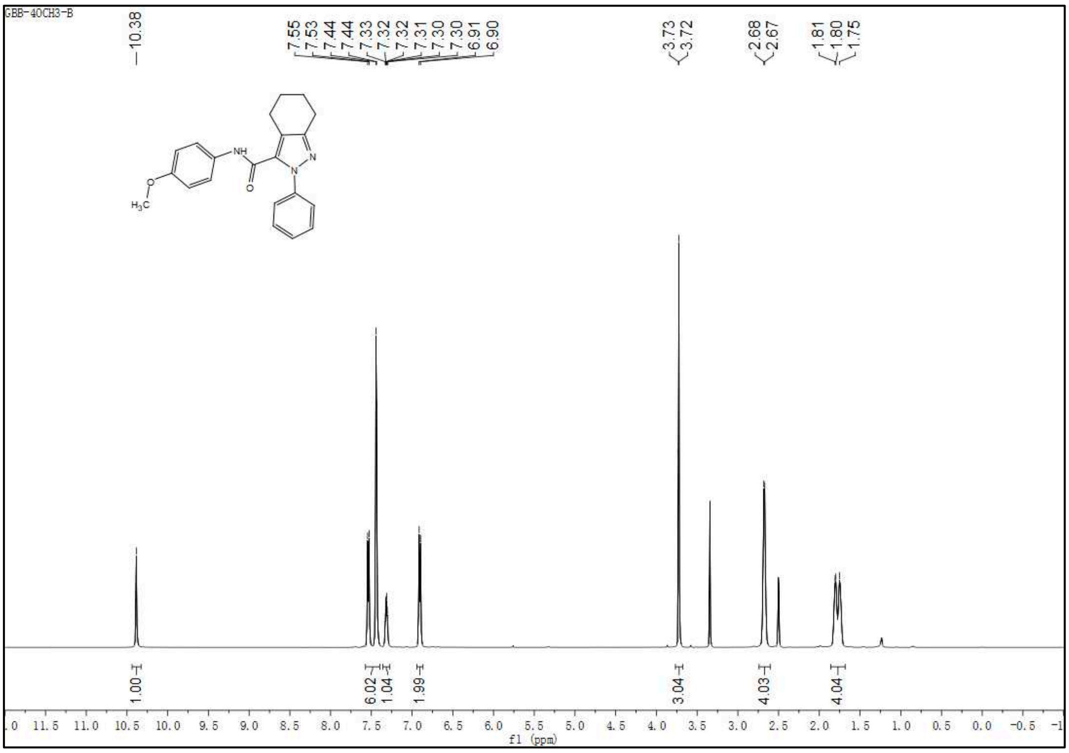

^{1}H NMR spectra of compound **6e**

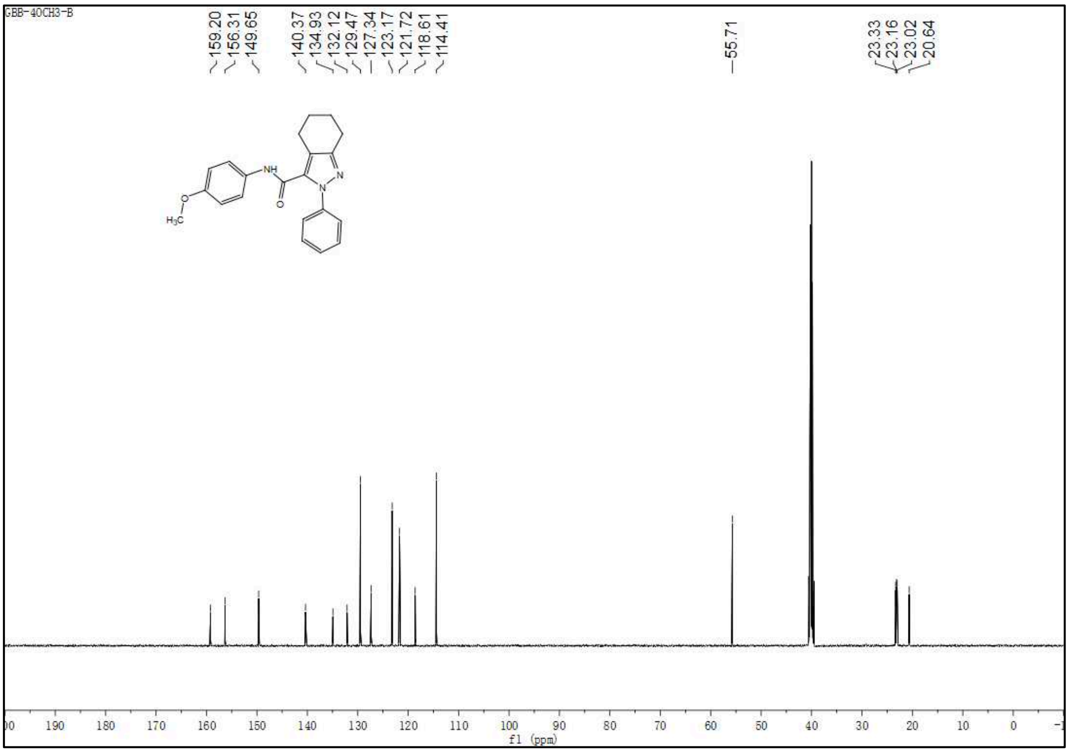

^{13}C NMR spectra of compound **6e**

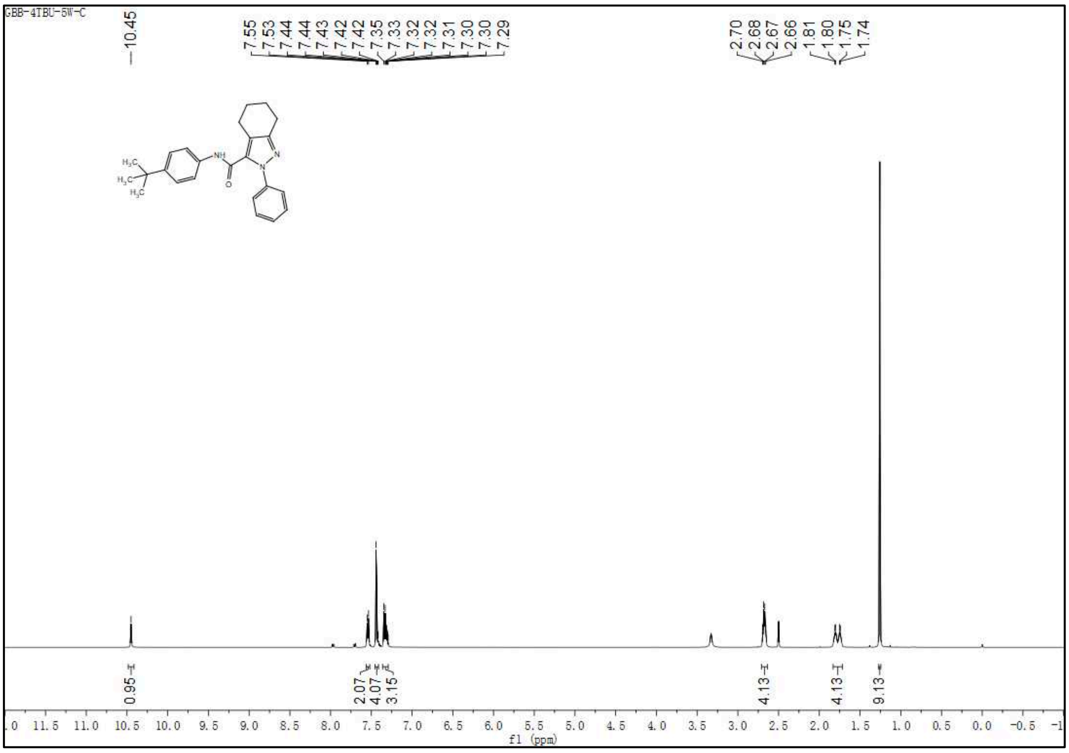

^{1}H NMR spectra of compound **6f**

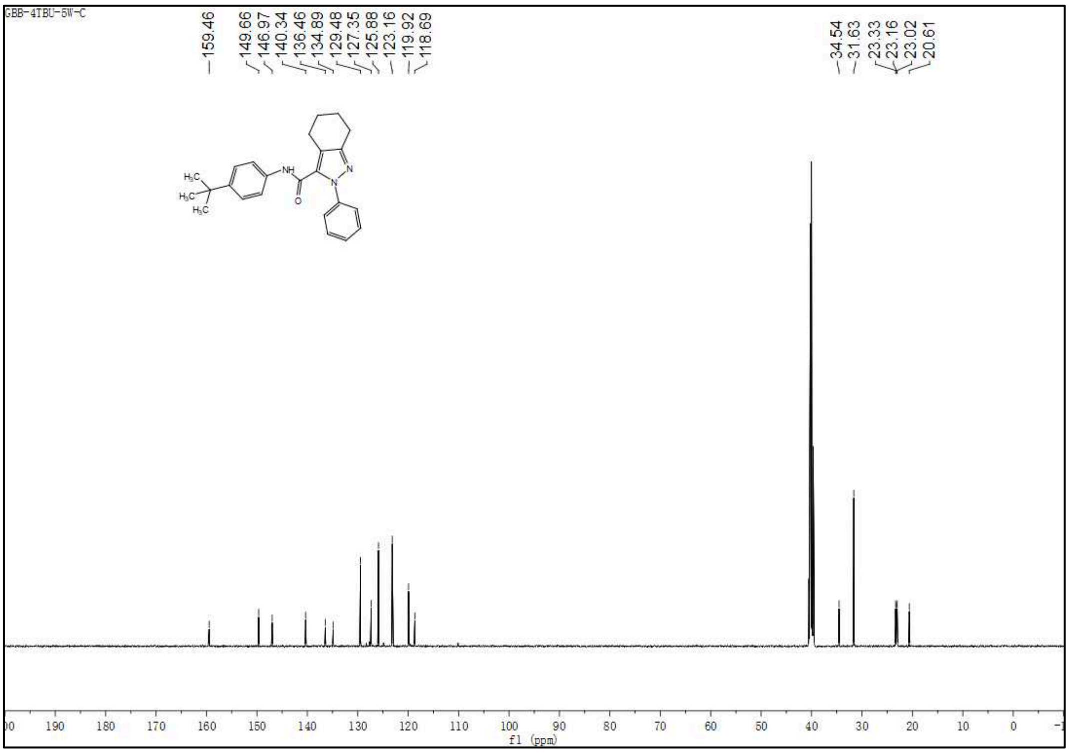

^{13}C NMR spectra of compound **6f**

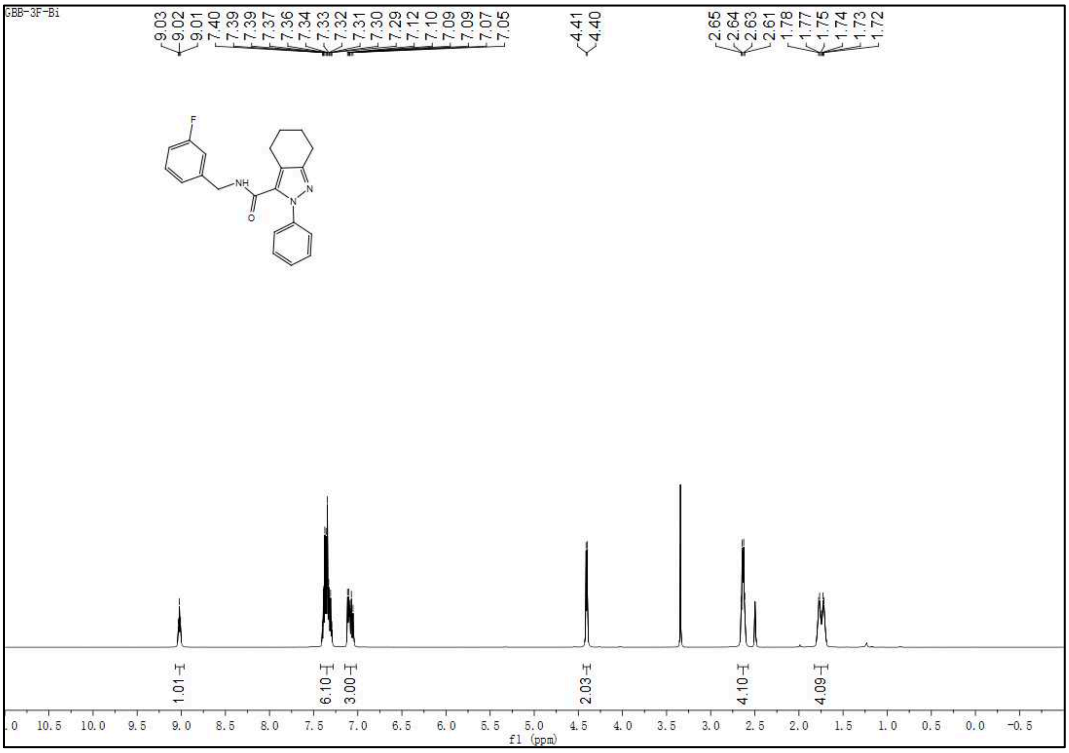

^{1}H NMR spectra of compound **6g**

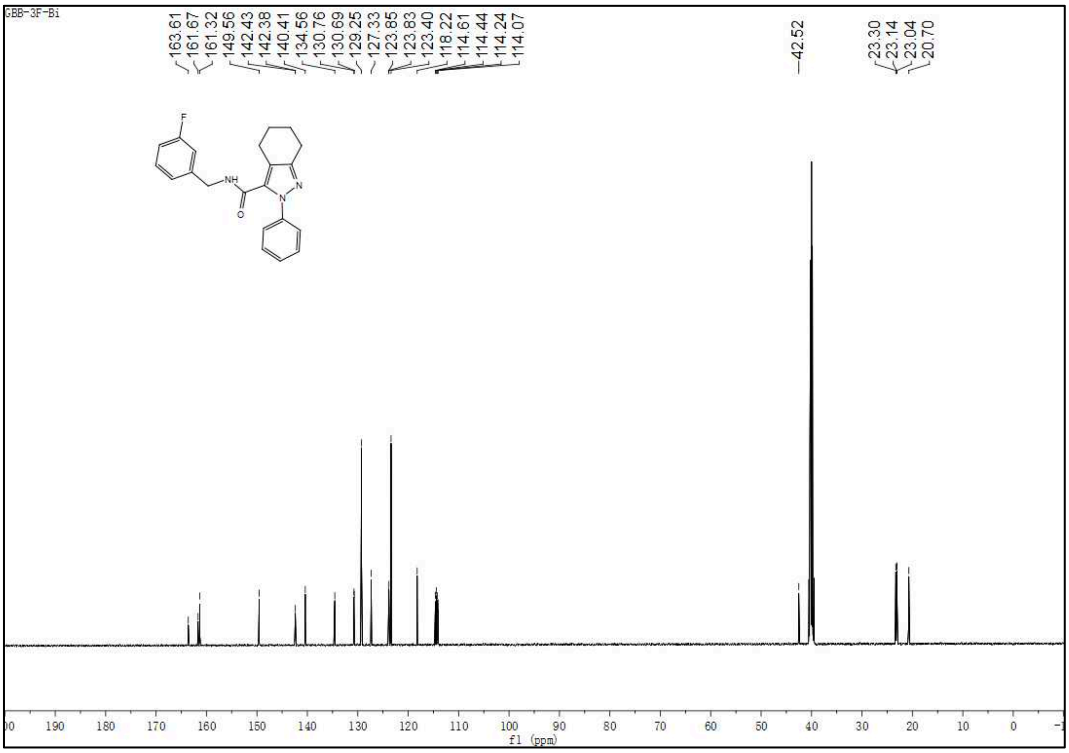

^{13}C NMR spectra of compound **6g**

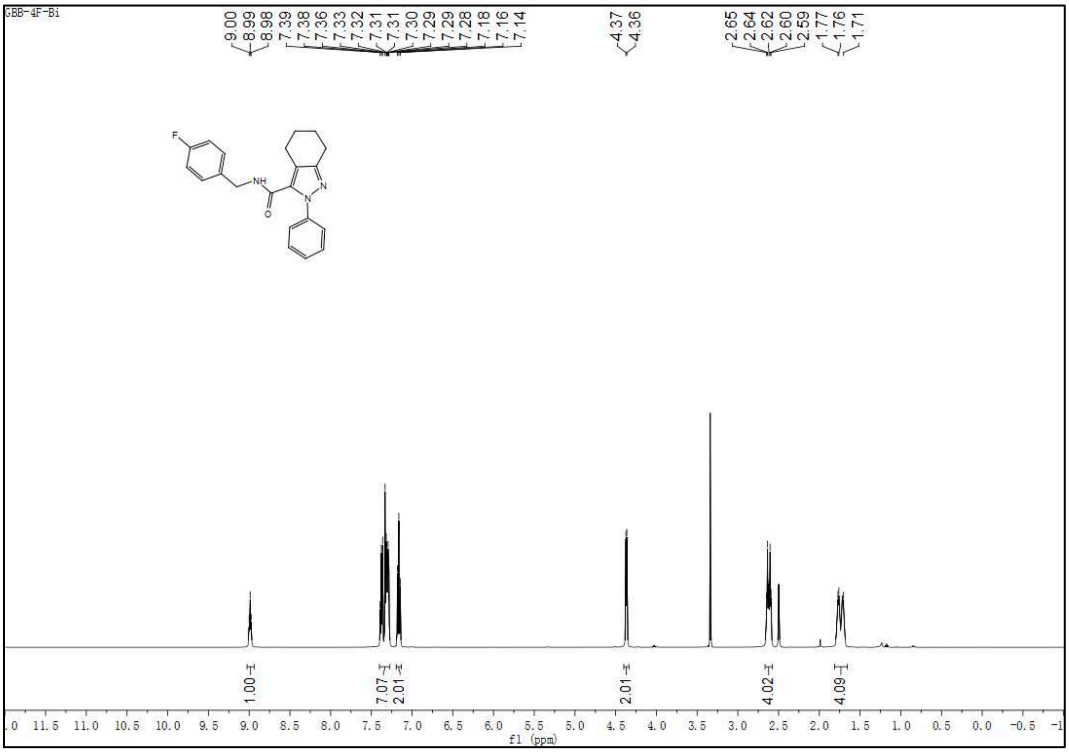

^{1}H NMR spectra of compound **6h**

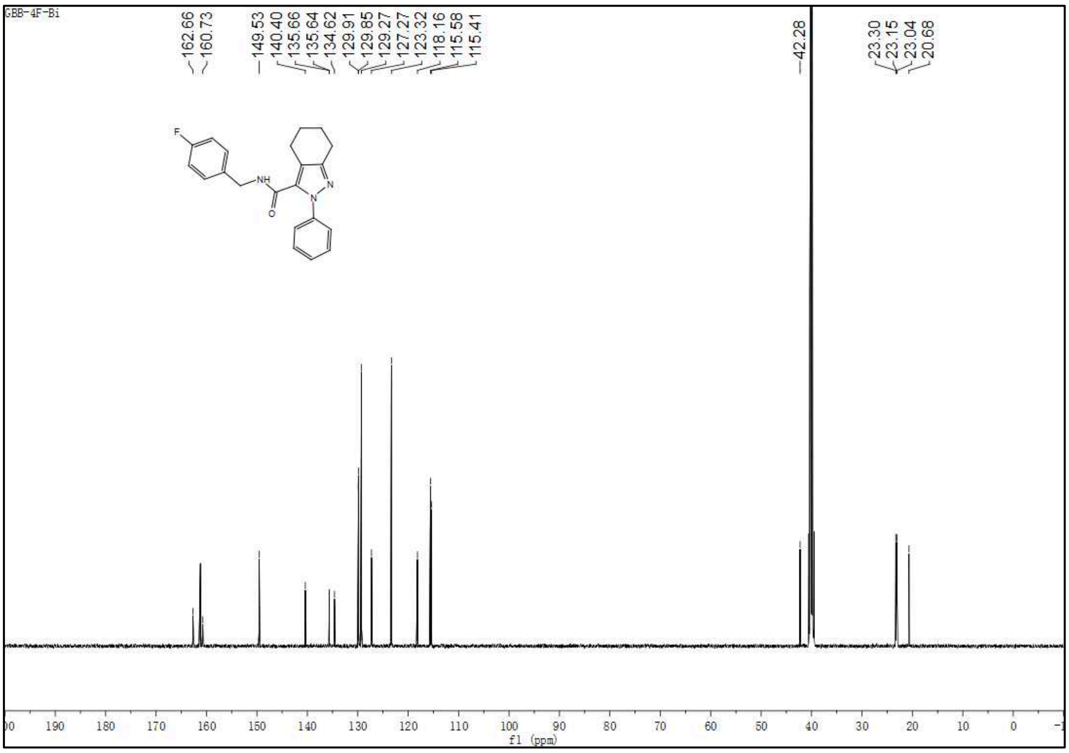

^{13}C NMR spectra of compound **6h**

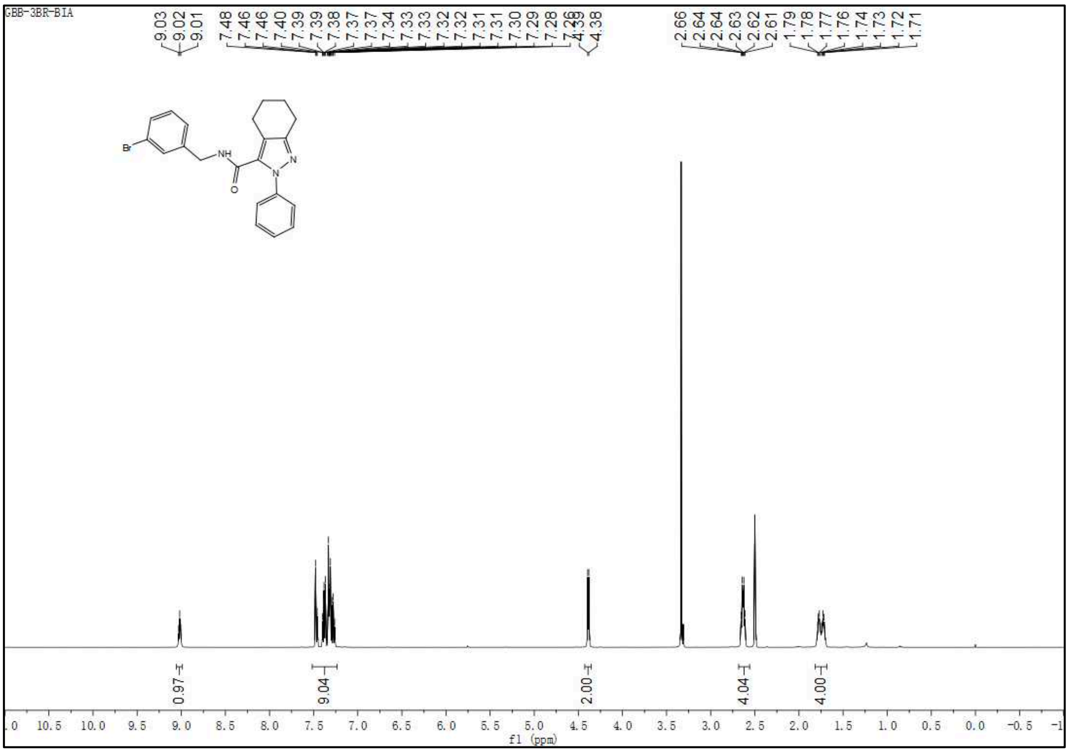

^{1}H NMR spectra of compound **6i**

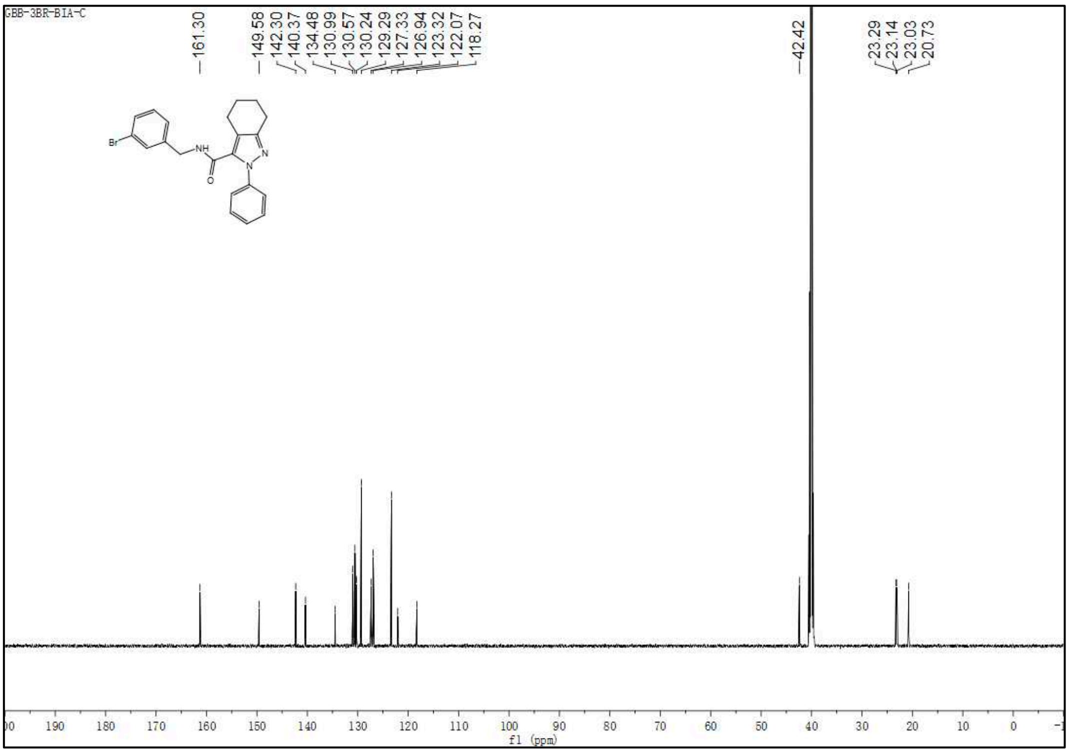

^{13}C NMR spectra of compound **6i**

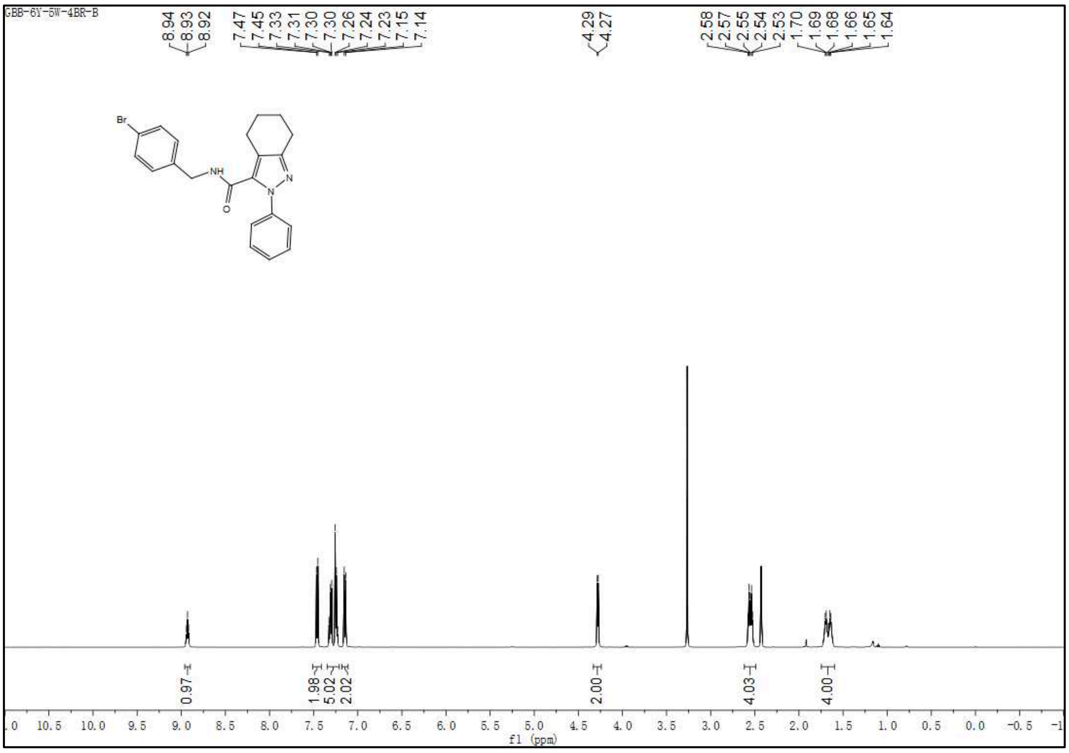

^{1}H NMR spectra of compound **6j**

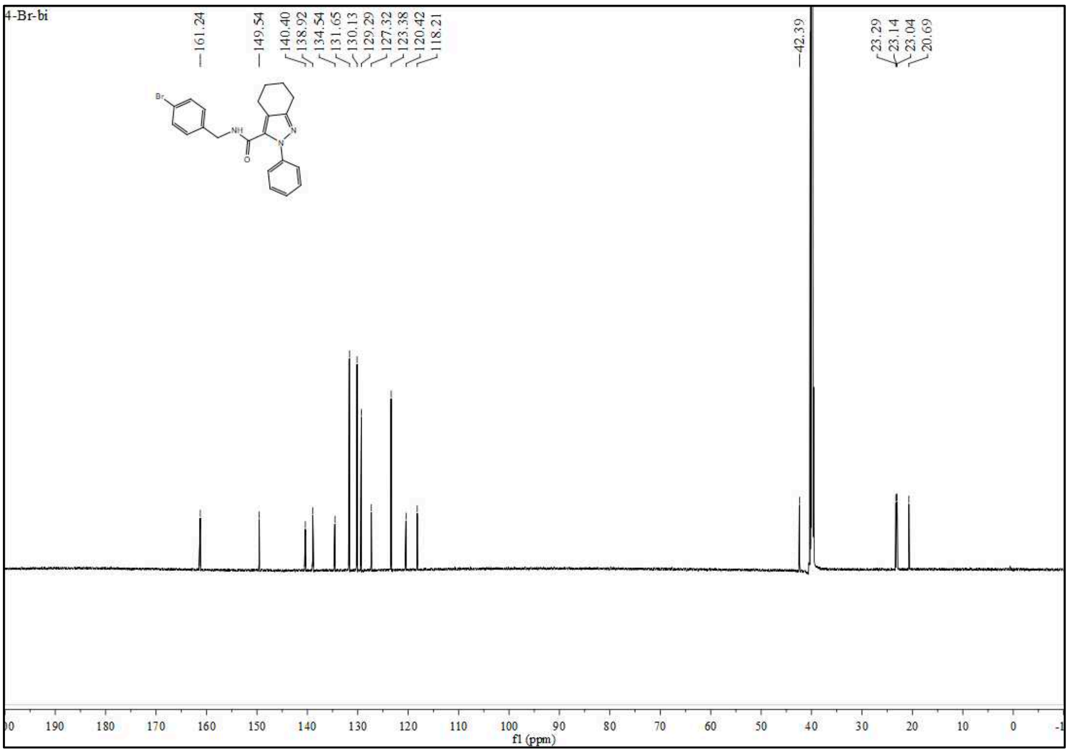

^{13}C NMR spectra of compound **6j**

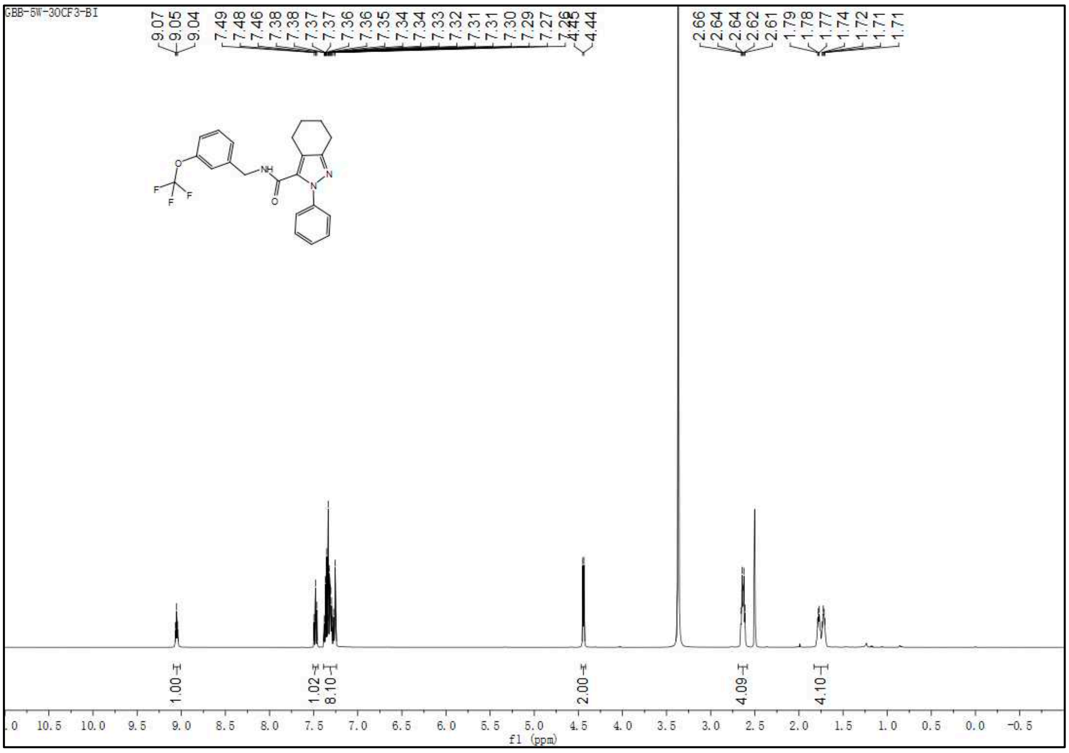

^{1}H NMR spectra of compound **6k**

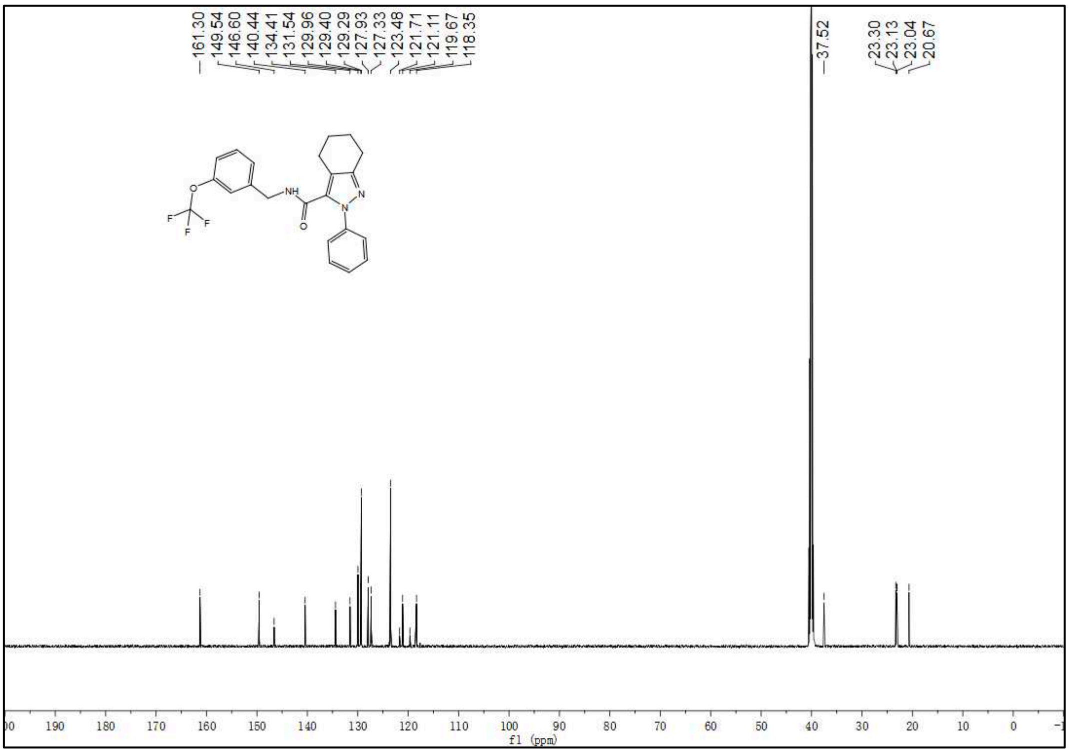

^{13}C NMR spectra of compound **6k**

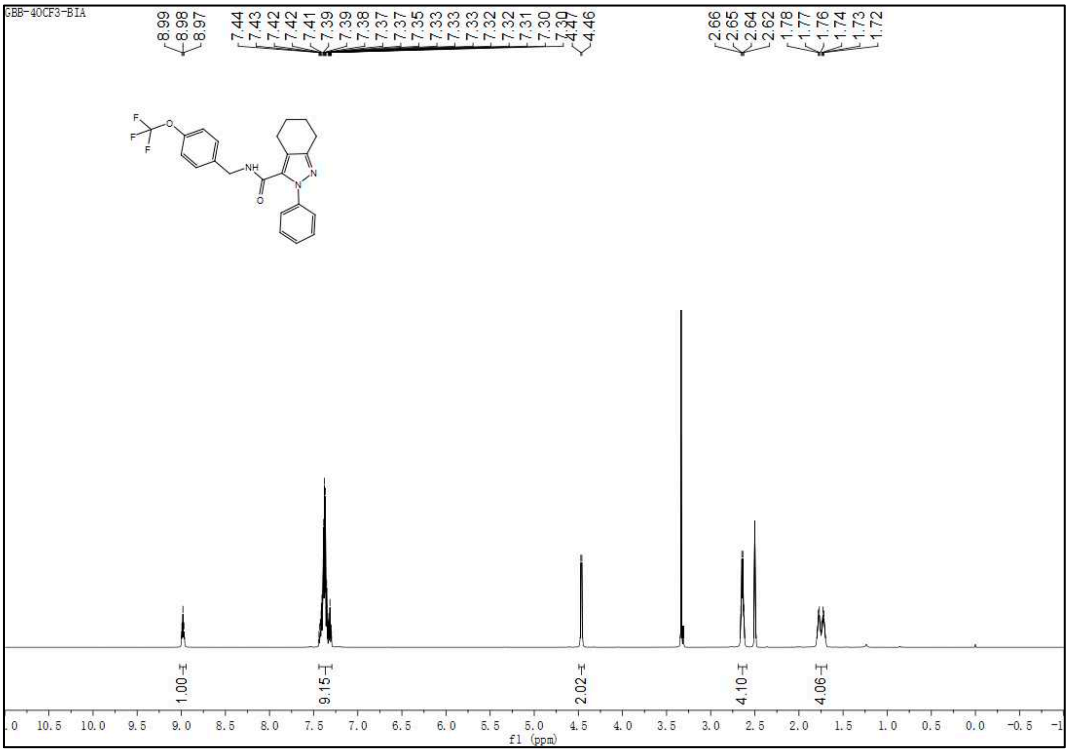

^{1}H NMR spectra of compound **6l**

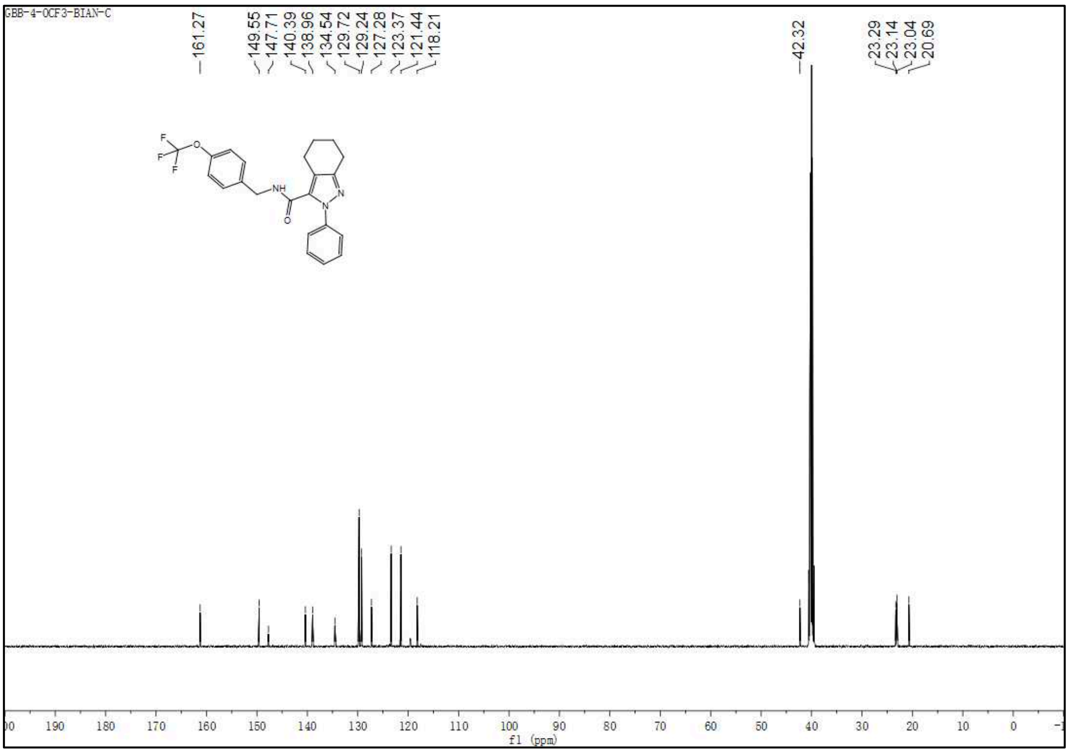

^{13}C NMR spectra of compound **6l**

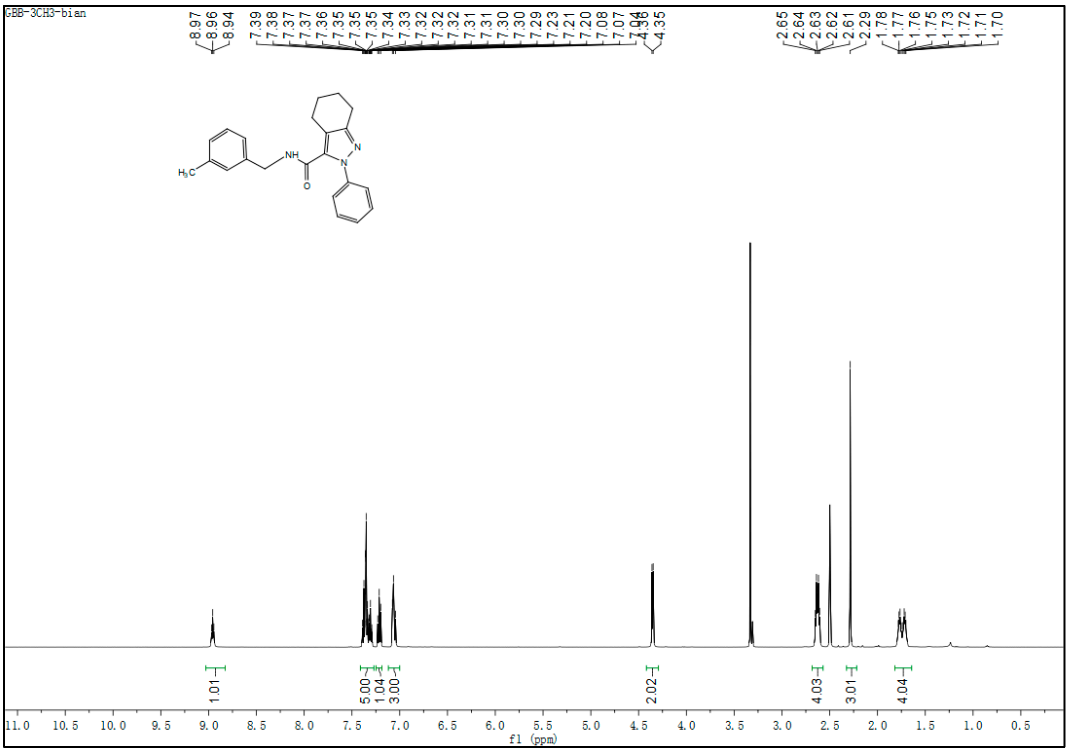

^{1}H NMR spectra of compound **6m**

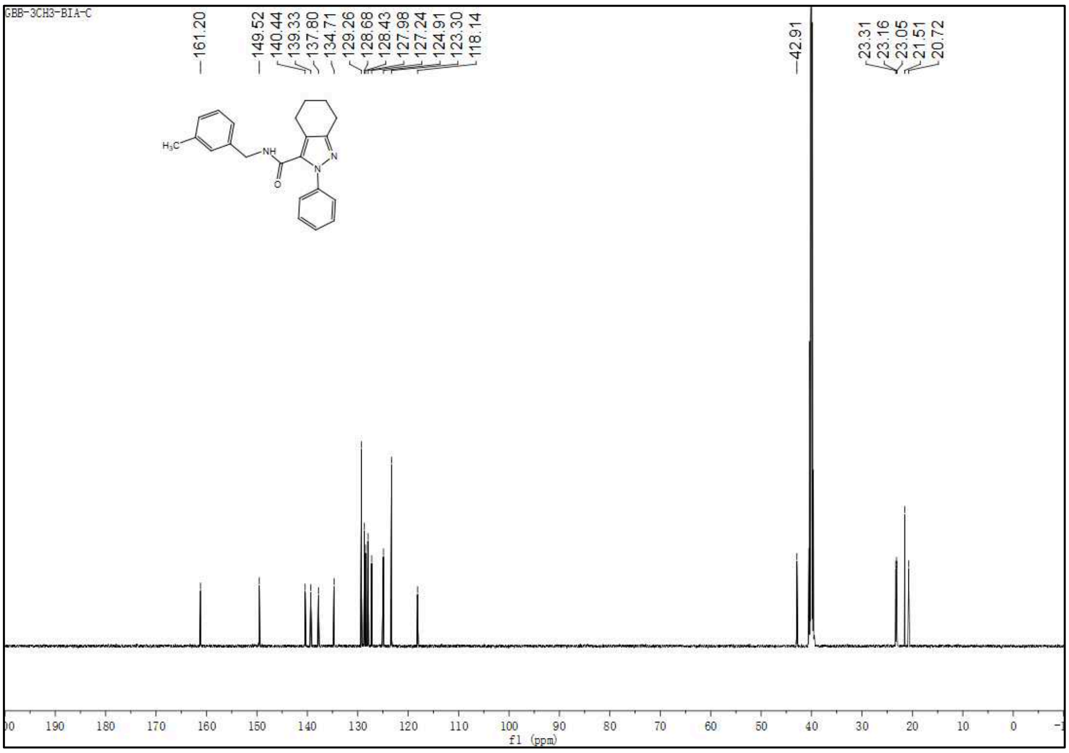

^{13}C NMR spectra of compound **6m**

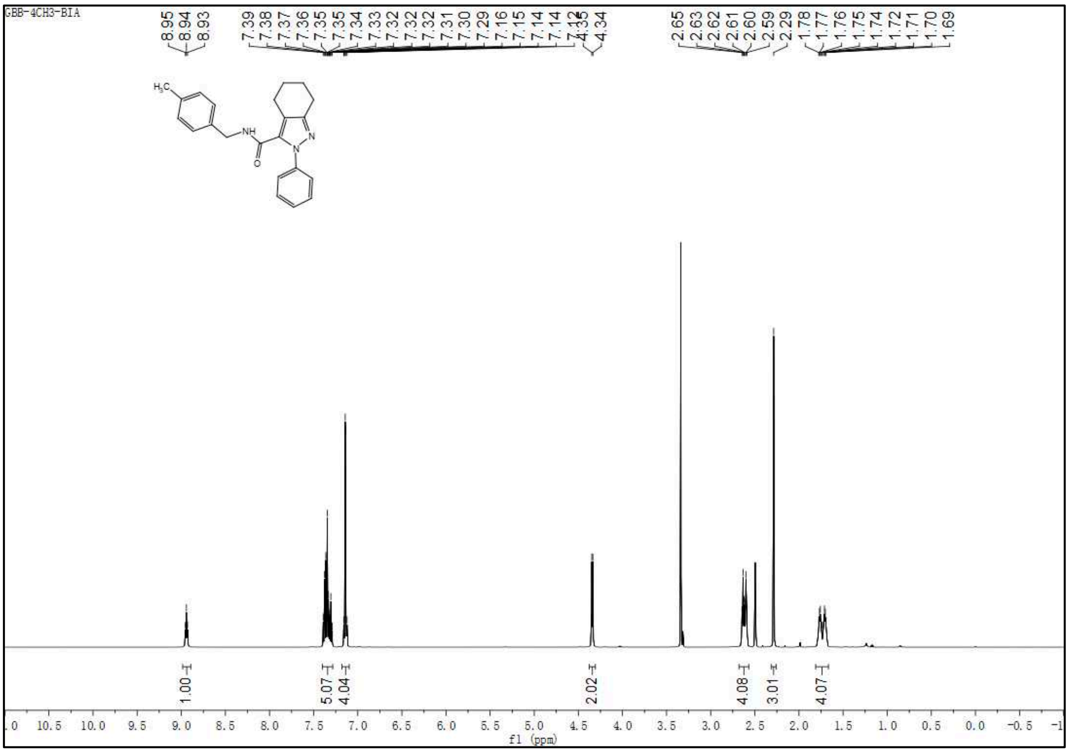

^{1}H NMR spectra of compound **6n**

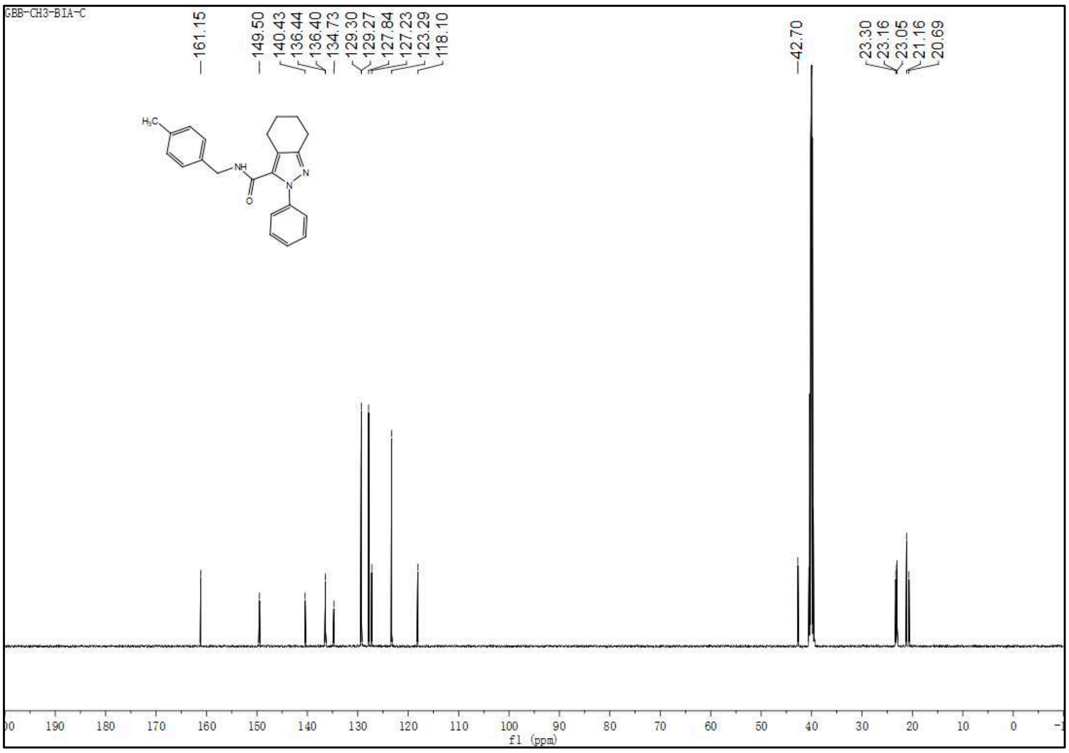

^{13}C NMR spectra of compound **6n**

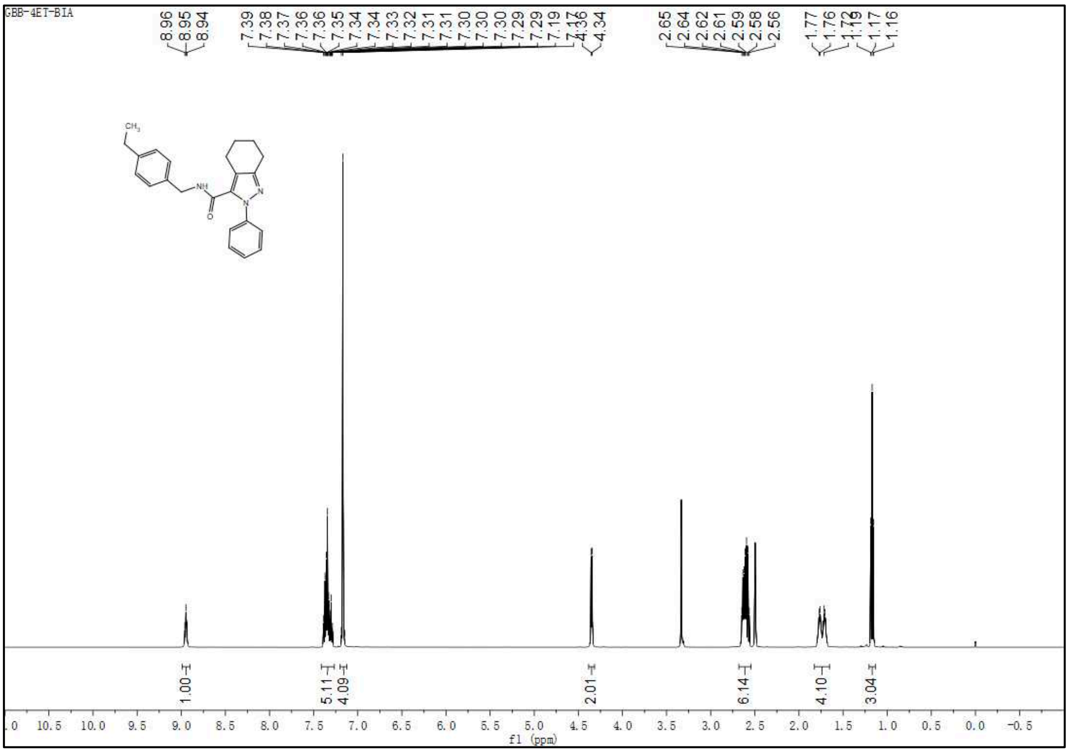

^{1}H NMR spectra of compound **6o**

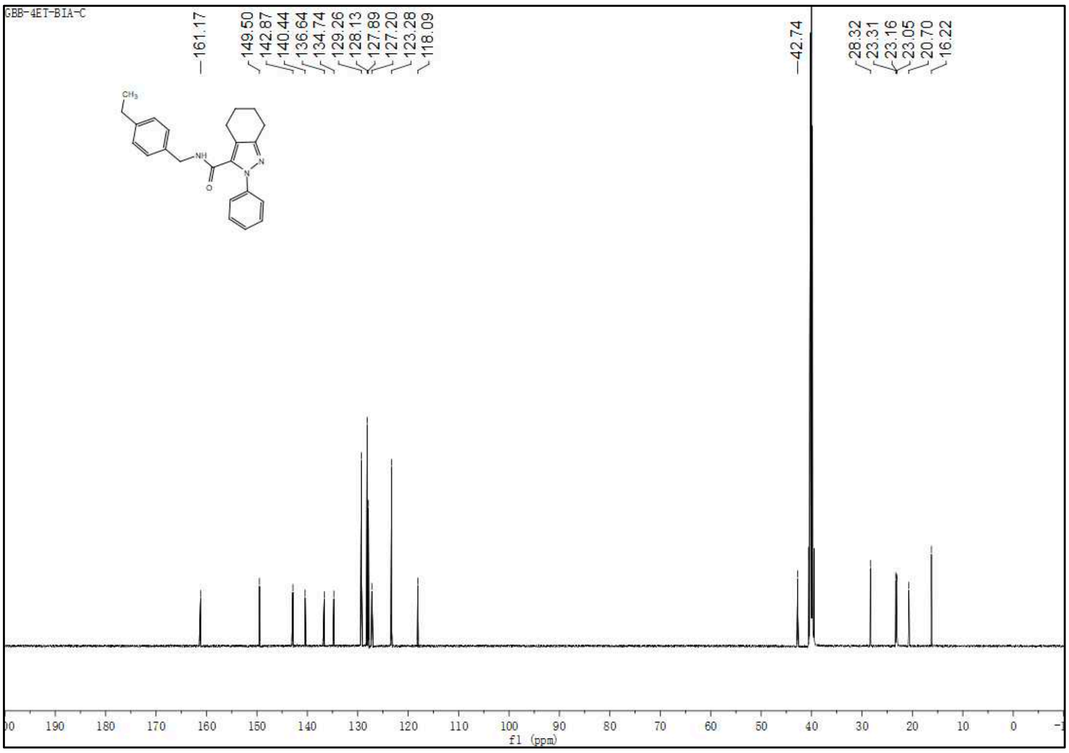

^{13}C NMR spectra of compound **6o**

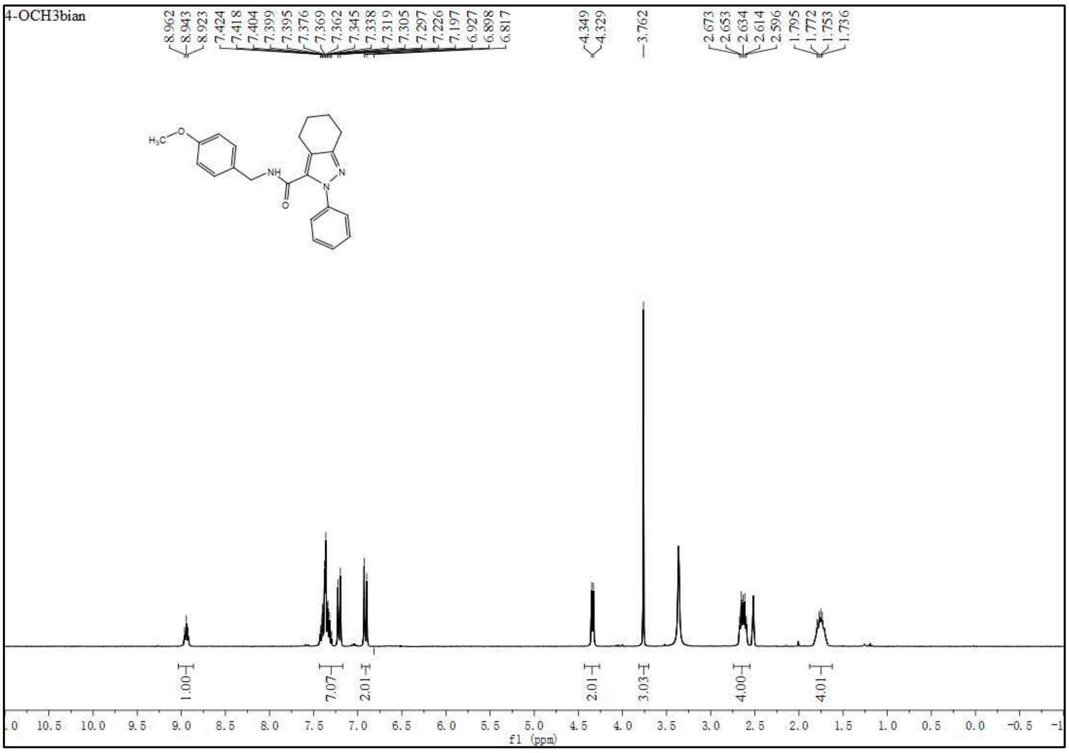

^1H NMR spectra of compound **6p**

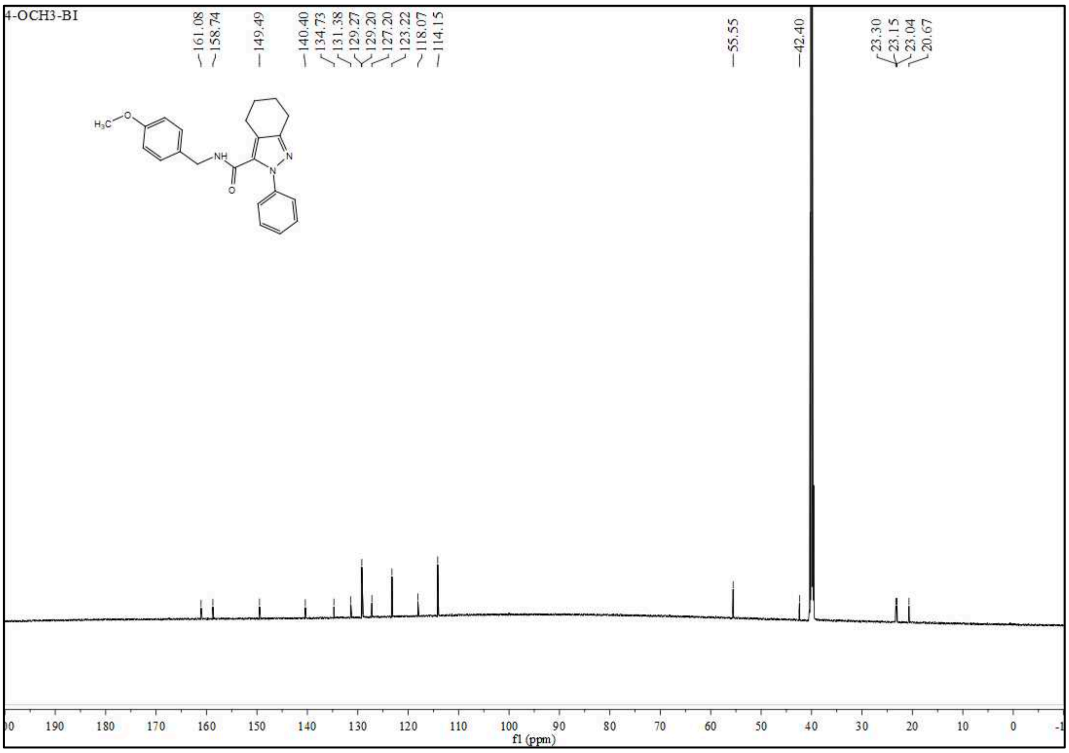

^{13}C NMR spectra of compound **6p**

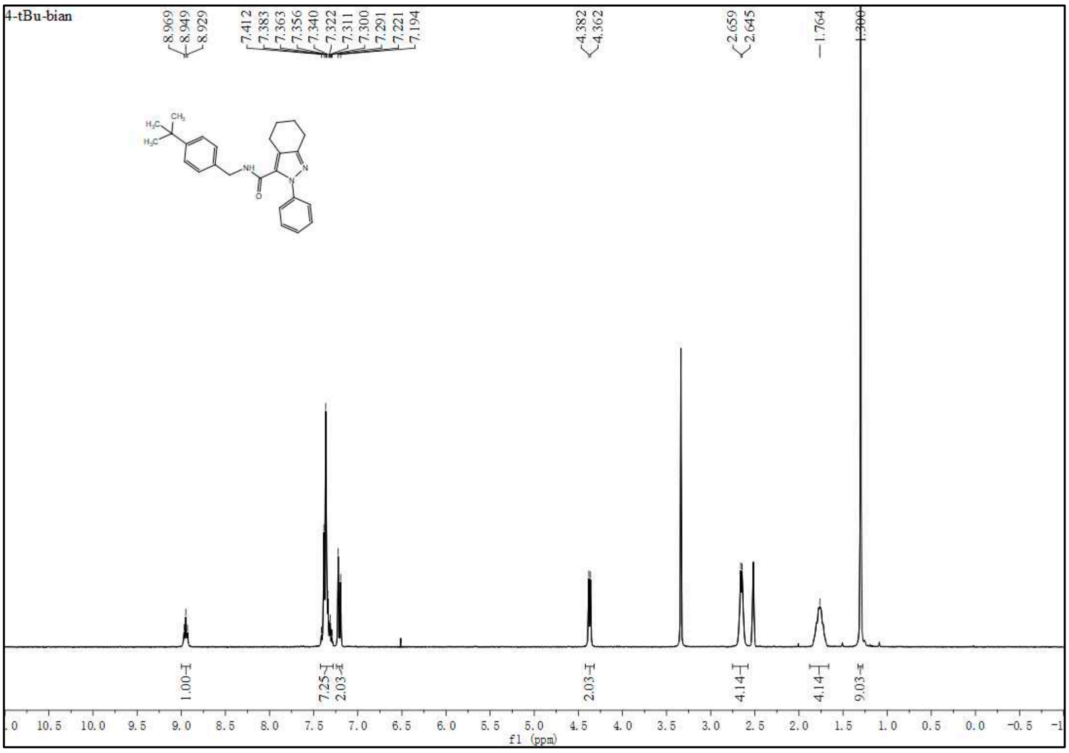

^{1}H NMR spectra of compound **6q**

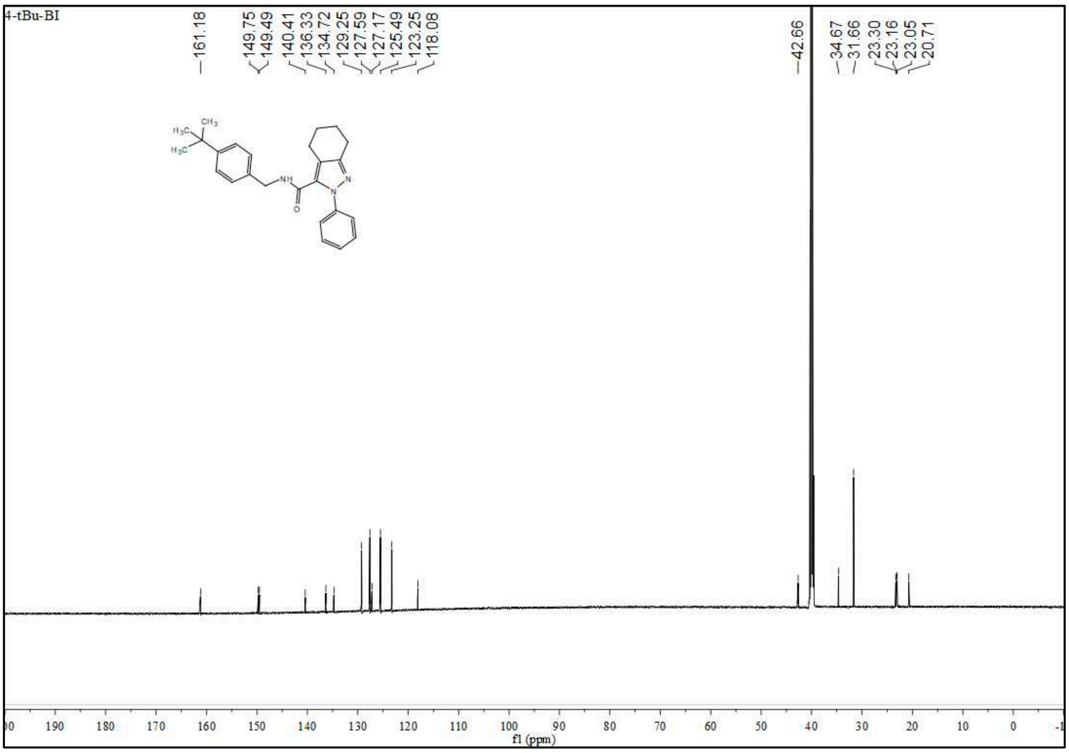

^{13}C NMR spectra of compound **6q**

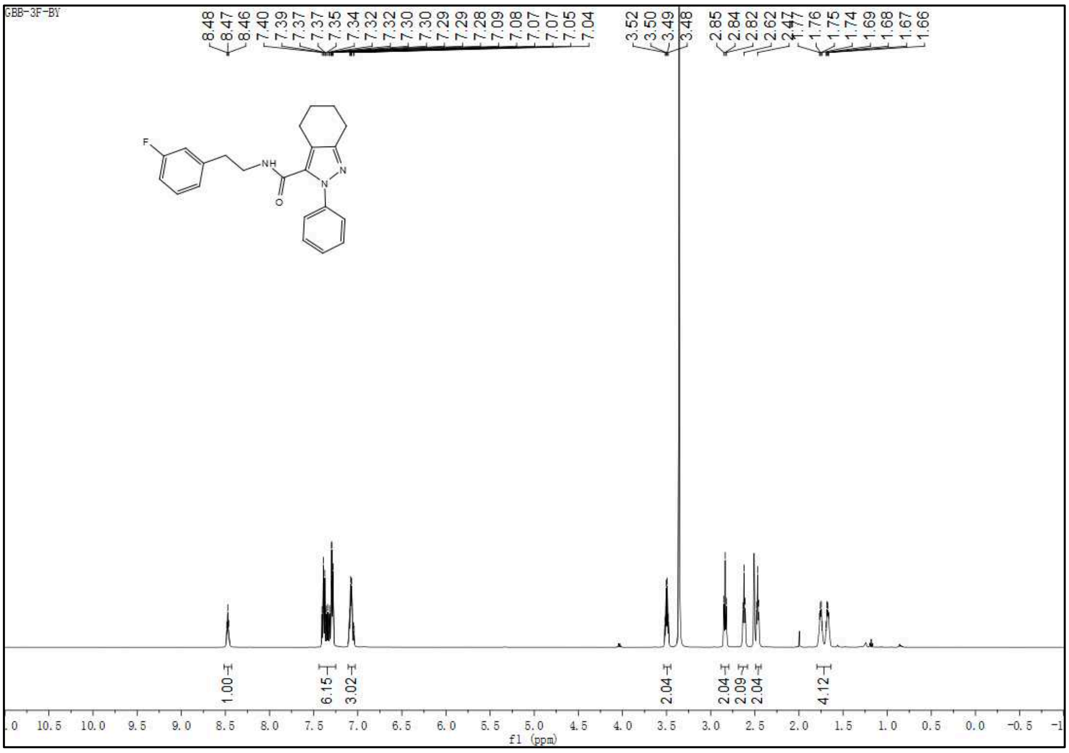

^{1}H NMR spectra of compound **6r**

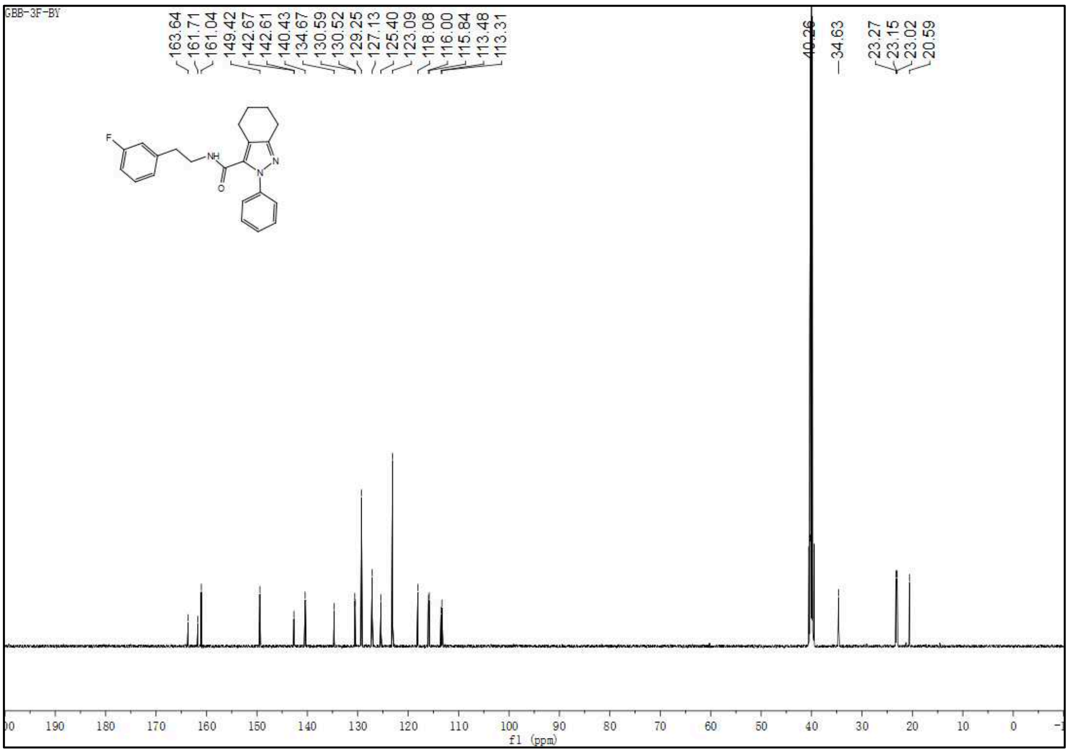

^{13}C NMR spectra of compound **6r**

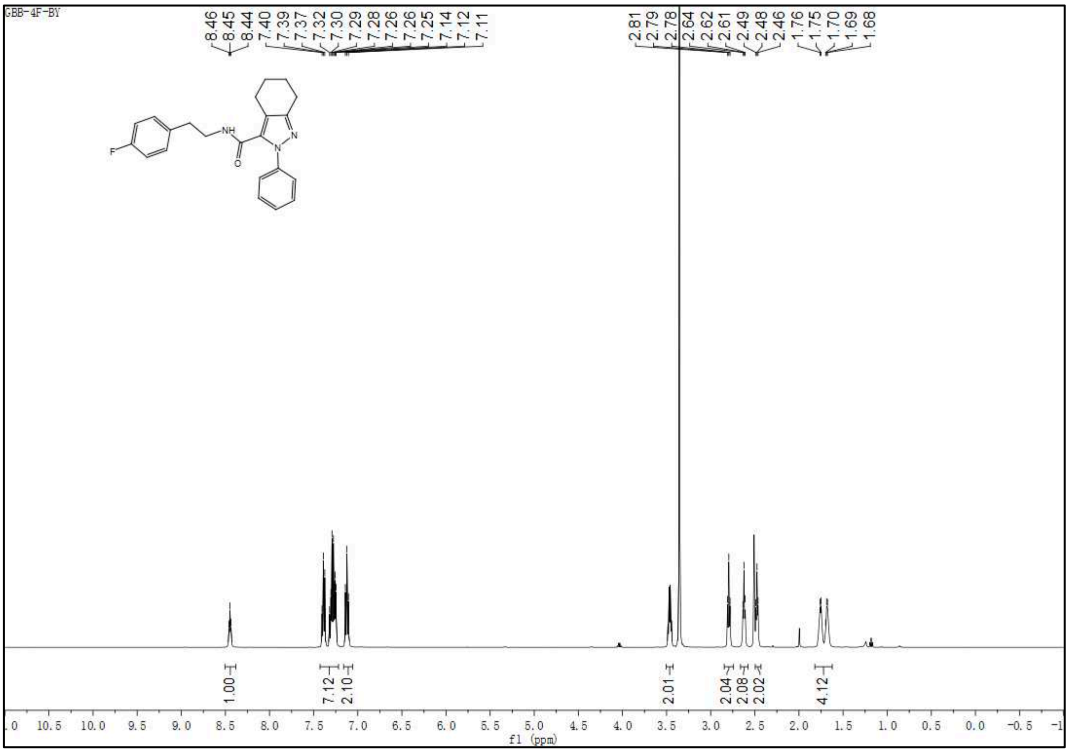

^{1}H NMR spectra of compound **6s**

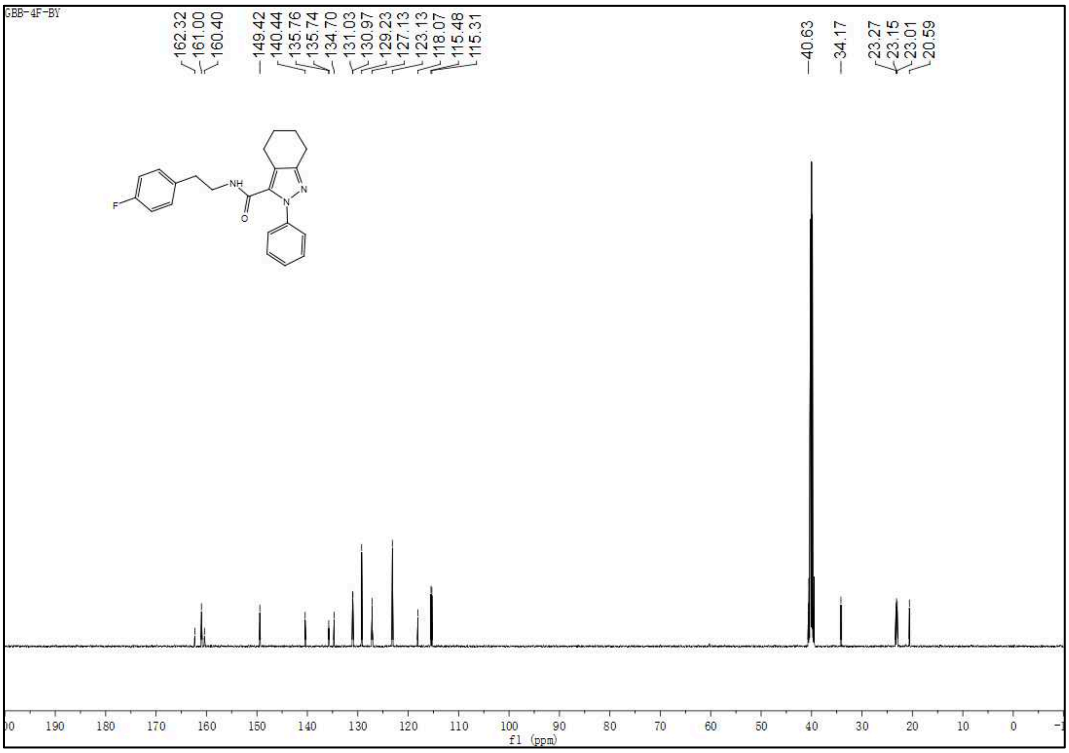

^{13}C NMR spectra of compound **6s**

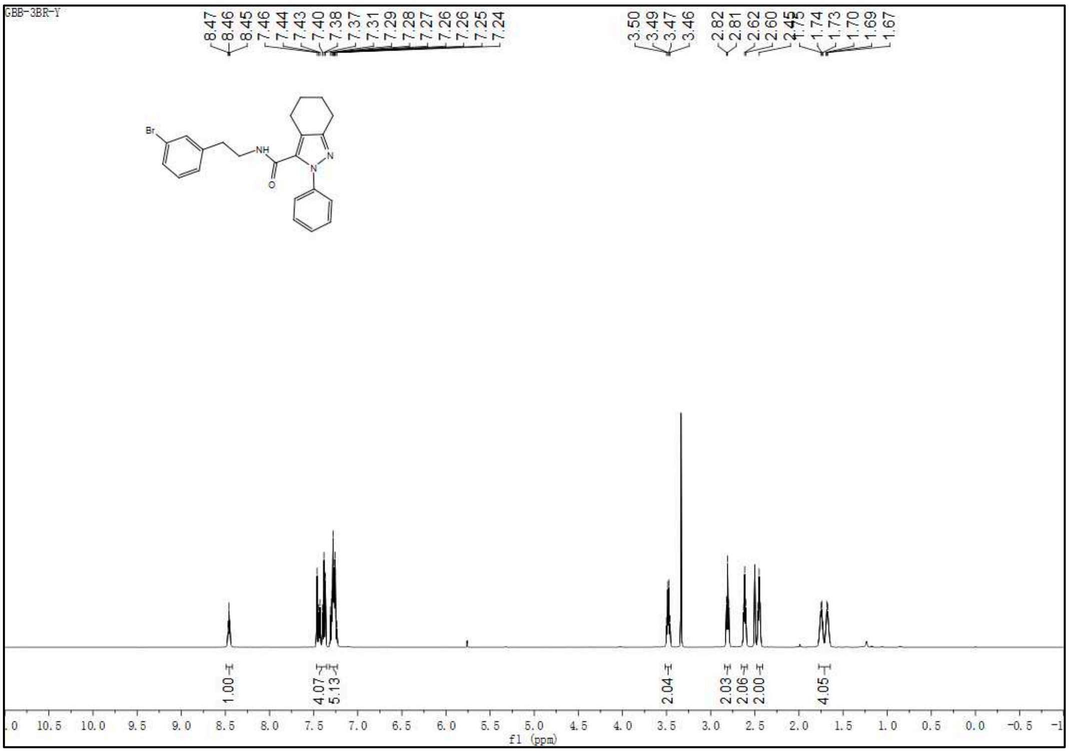

^{1}H NMR spectra of compound **6t**

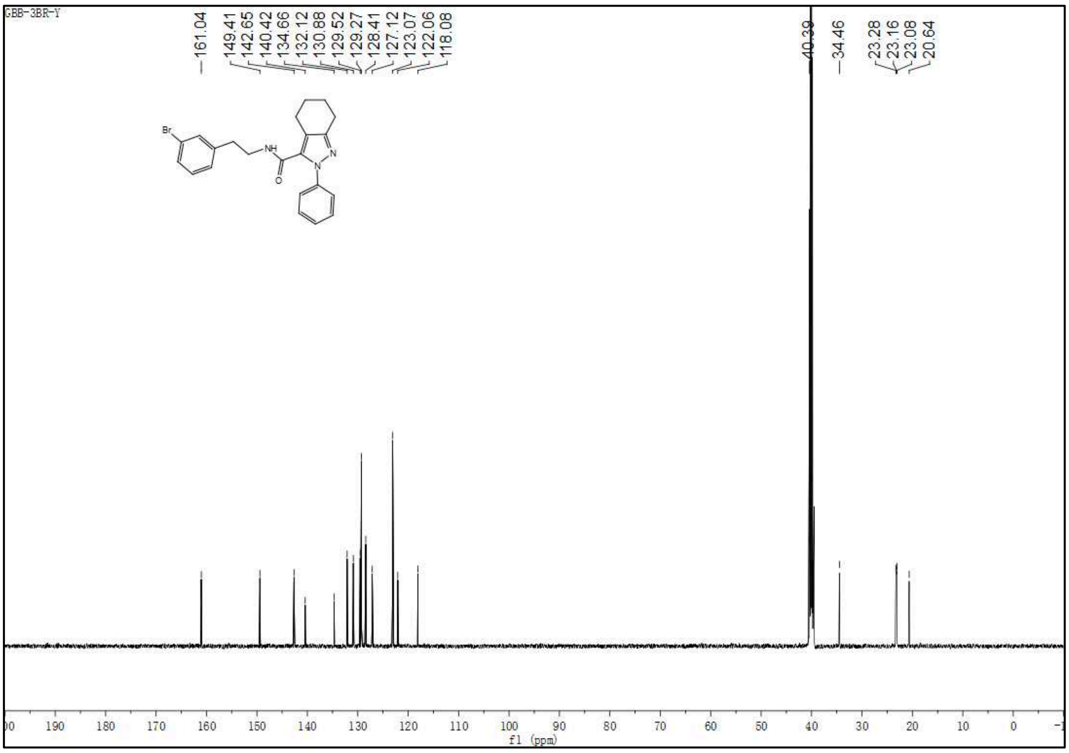

^{13}C NMR spectra of compound **6t**

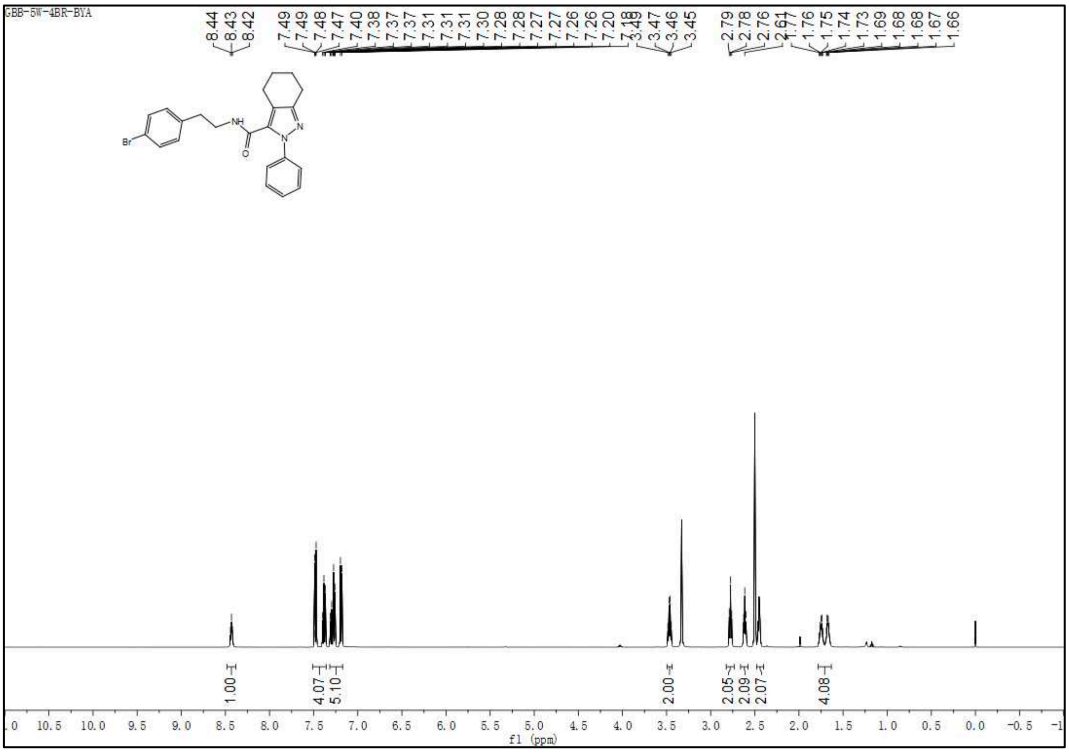

^{1}H NMR spectra of compound **6u**

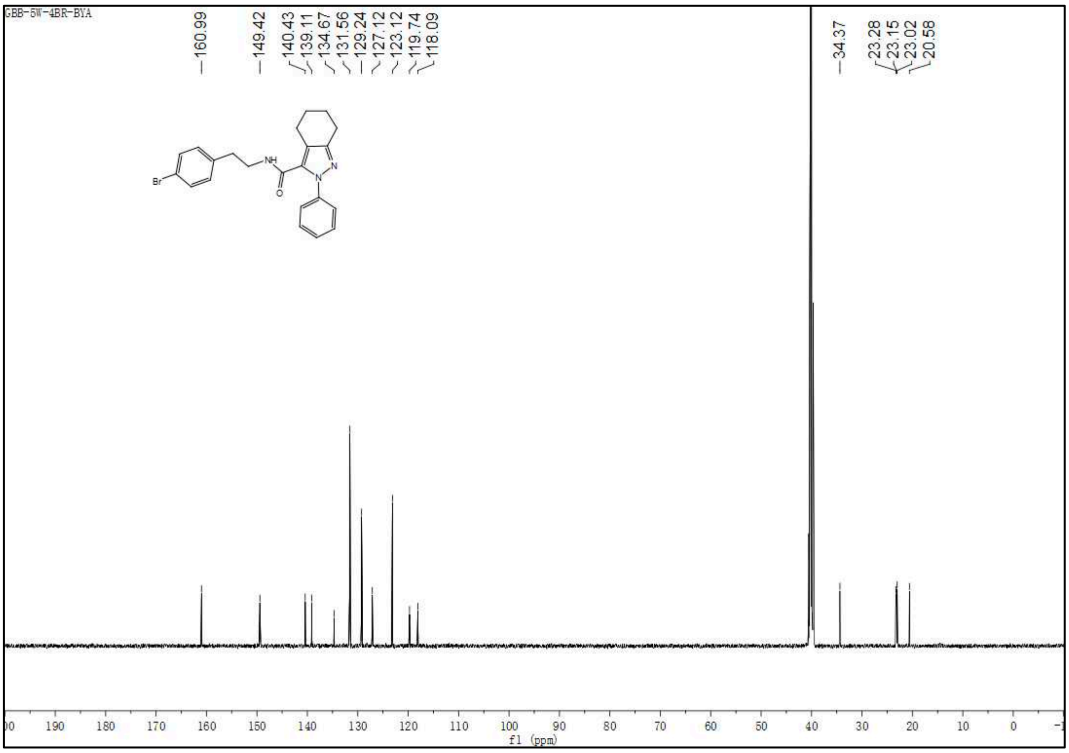

^{13}C NMR spectra of compound **6u**

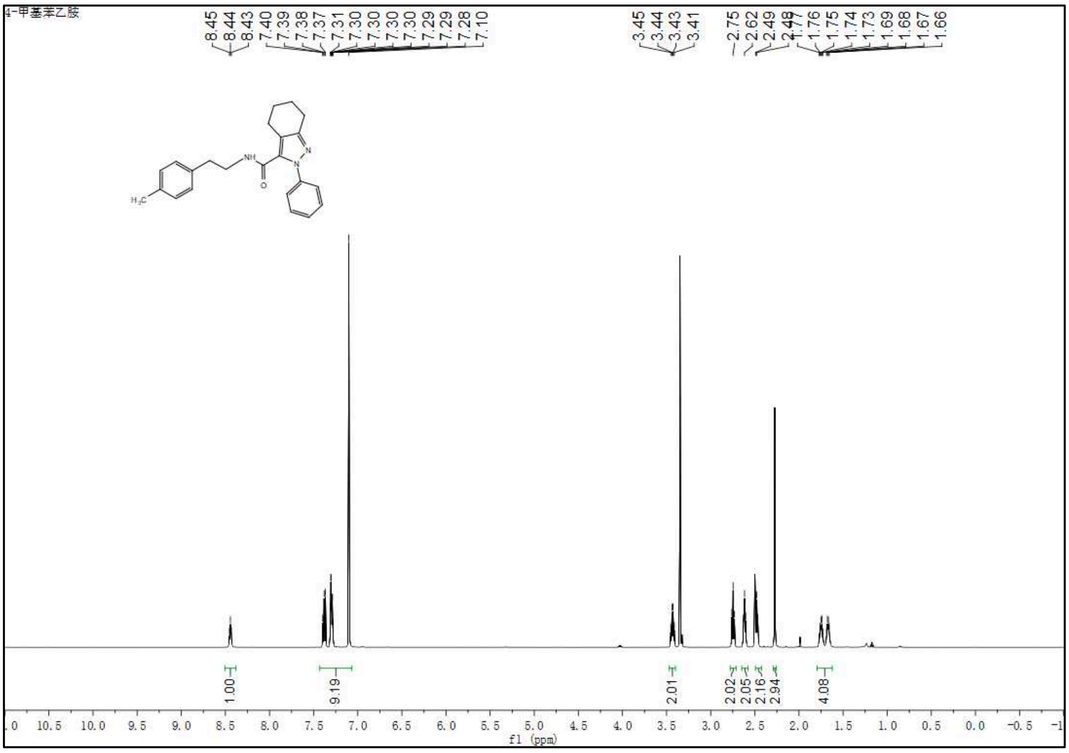

^{1}H NMR spectra of compound **6v**

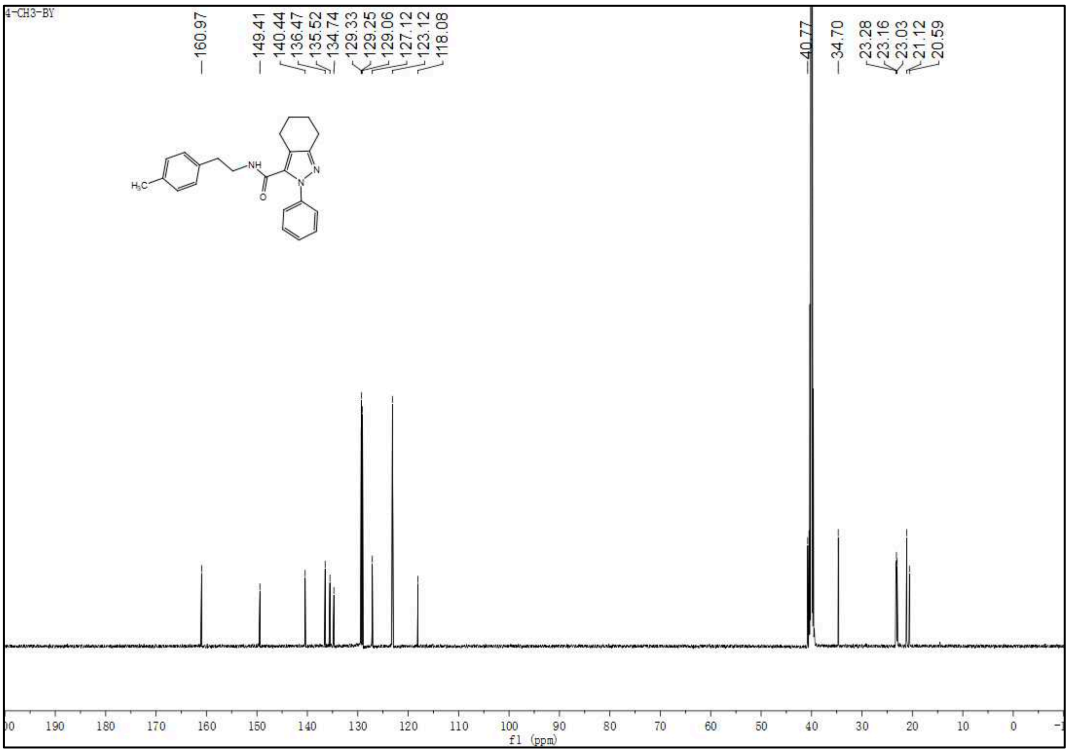

^{13}C NMR spectra of compound **6v**

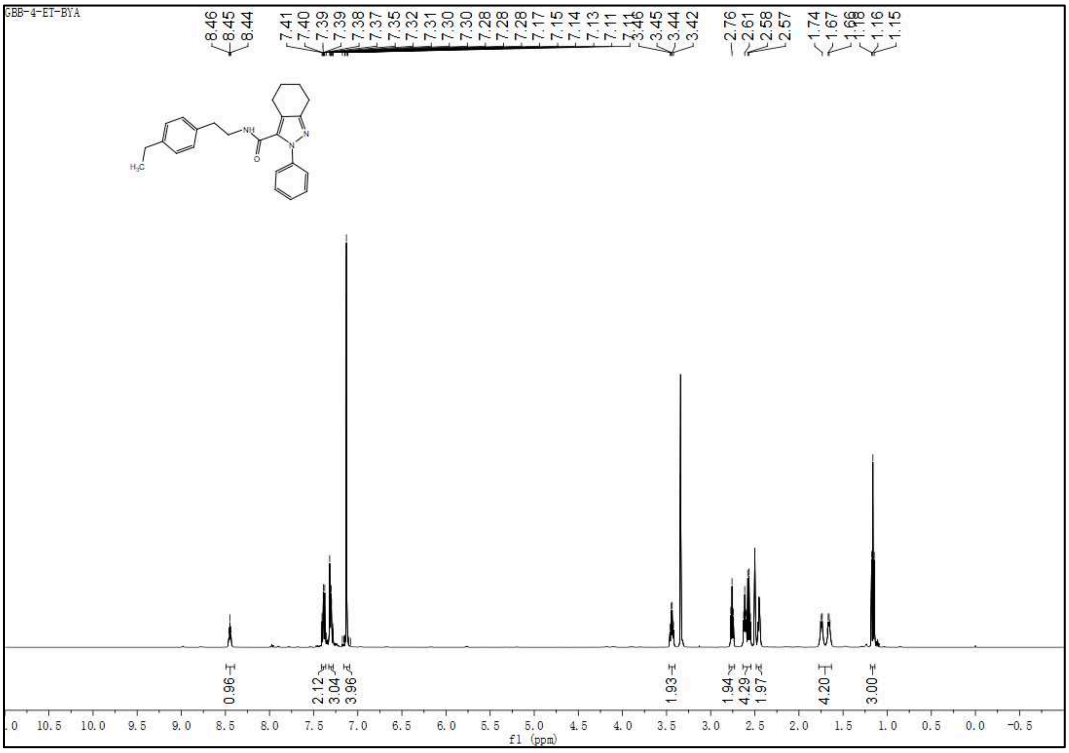

^{1}H NMR spectra of compound **6w**

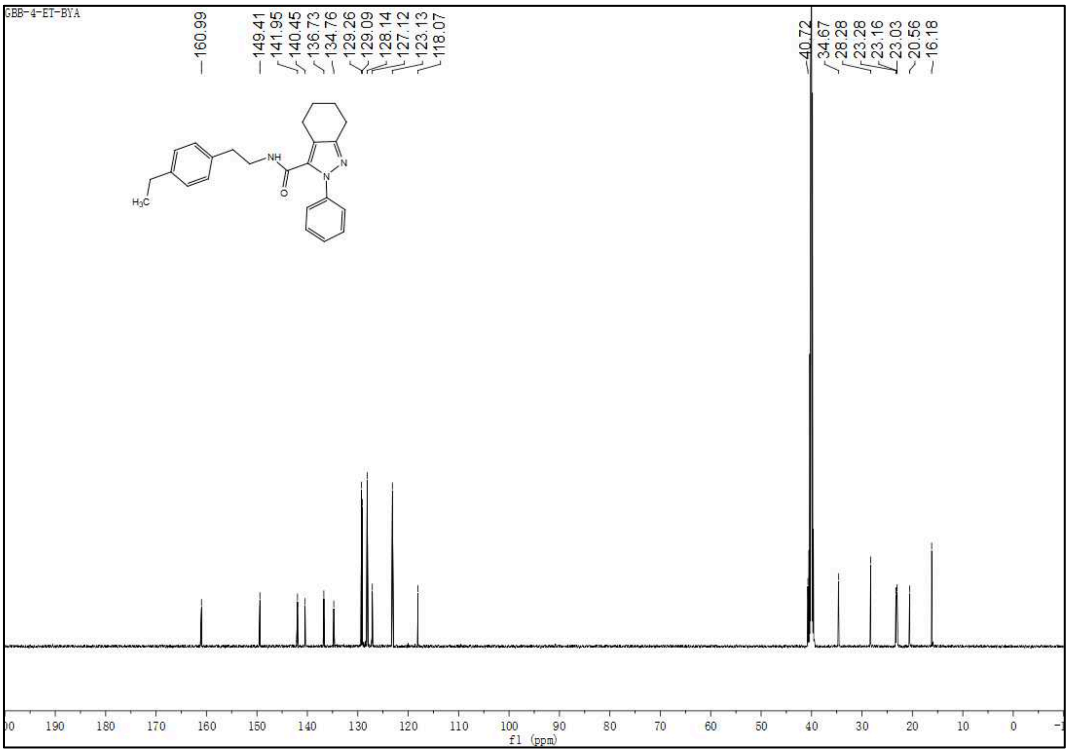

^{13}C NMR spectra of compound **6w**

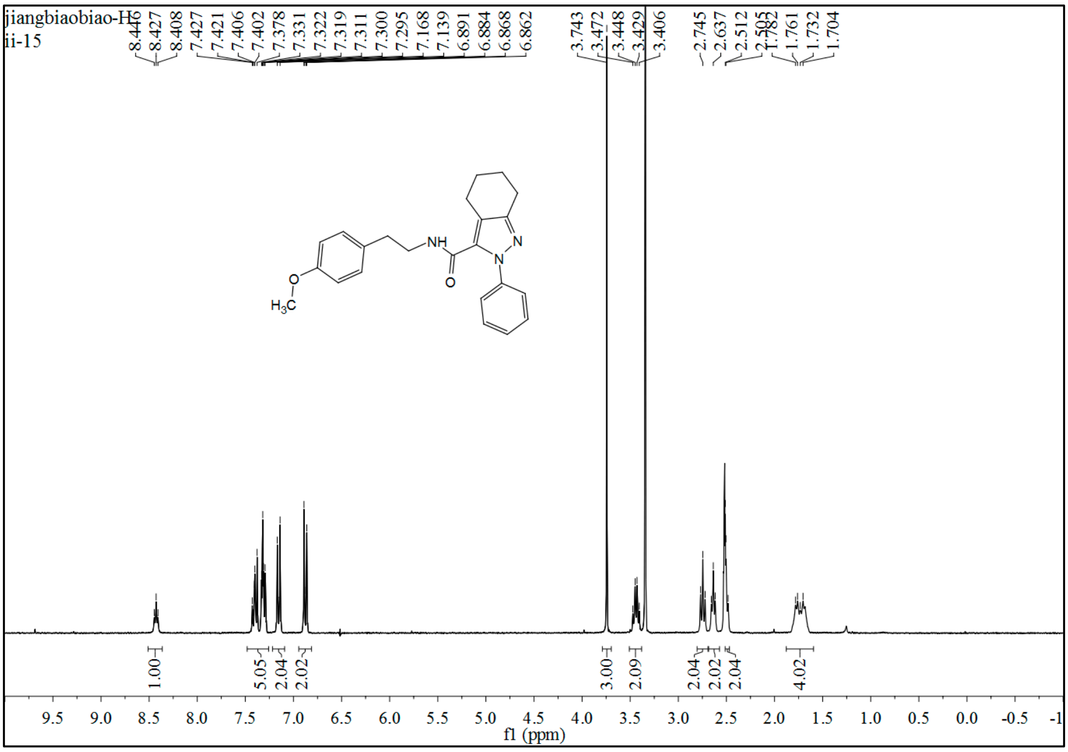

^{1}H NMR spectra of compound **6x**

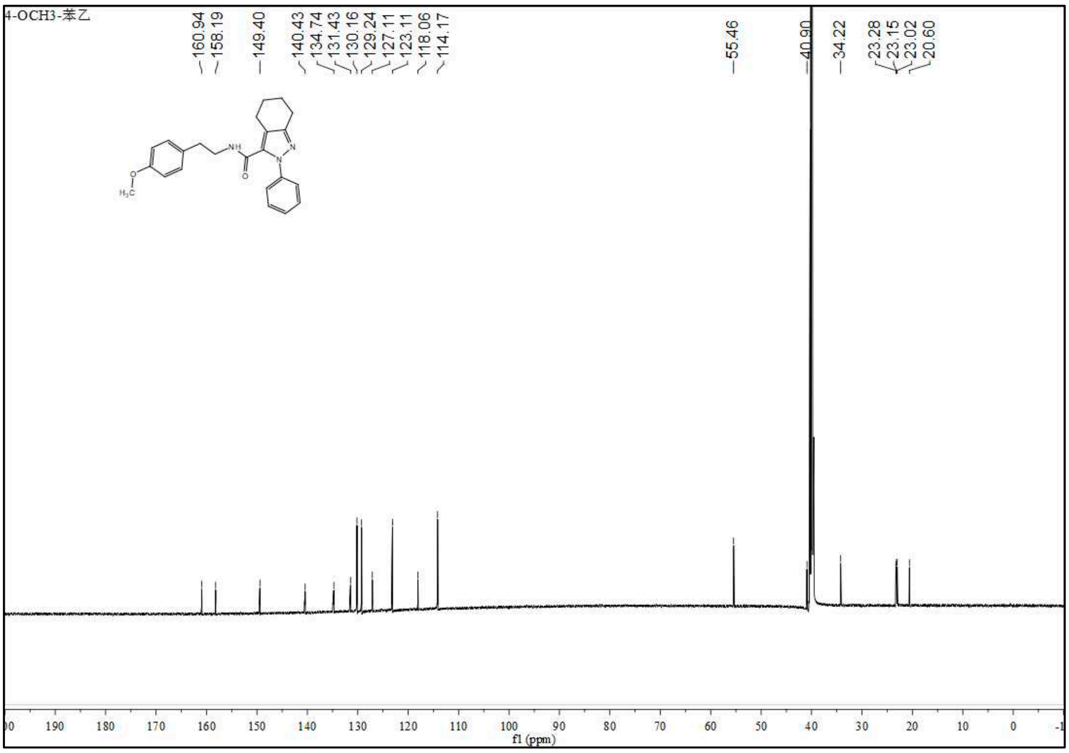

^{13}C NMR spectra of compound **6x**

HRMS Spectrum of **6a**

Sample No.	Formula (M)	Ion Formula	Measured m/z	Calc m/z	Diff (ppm)
**6a**	$C_{20}H_{18}BrN_3O$	$C_{20}H_{19}BrN_3O$	396.0709	396.0706	0.76

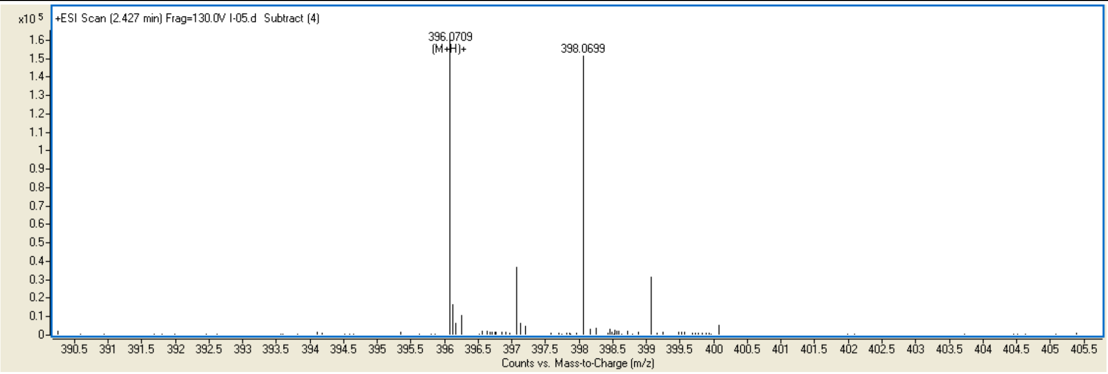

HRMS Spectrum of **6b**

Sample No.	Formula (M)	Ion Formula	Measured m/z	Calc m/z	Diff (ppm)
**6b**	$C_{20}H_{19}BrN_3O$	$C_{20}H_{19}BrN_3O$	396.0706	396.0706	0

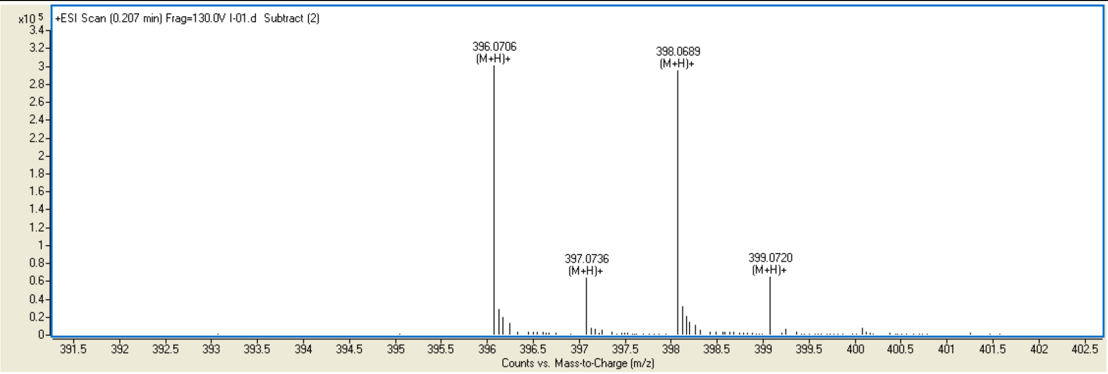

HRMS Spectrum of **6c**

Sample No.	Formula (M)	Ion Formula	Measured m/z	Calc m/z	Diff (ppm)
**6c**	$C_{22}H_{23}N_3O$	$C_{22}H_{24}N_3O$	346.1916	346.1914	0.58

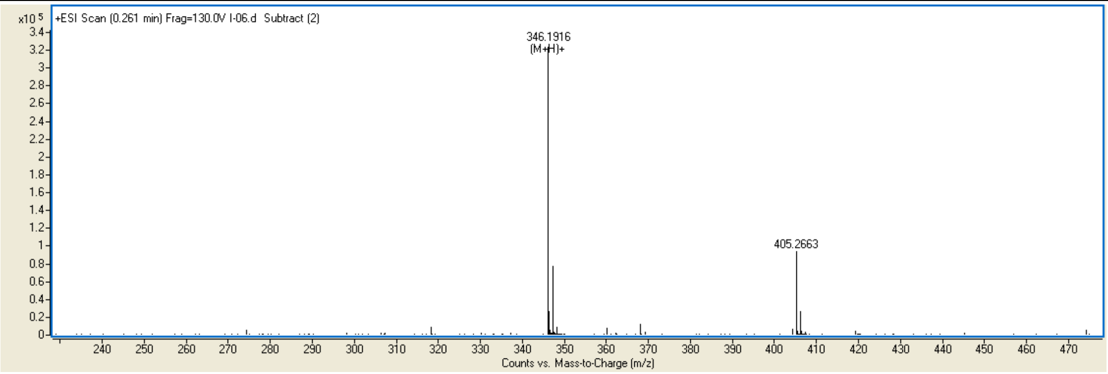

HRMS Spectrum of **6d**

Sample No.	Formula (M)	Ion Formula	Measured m/z	Calc m/z	Diff (ppm)
**6d**	$C_{22}H_{23}N_3O$	$C_{22}H_{24}N_3O$	346.1915	346.1914	0.29

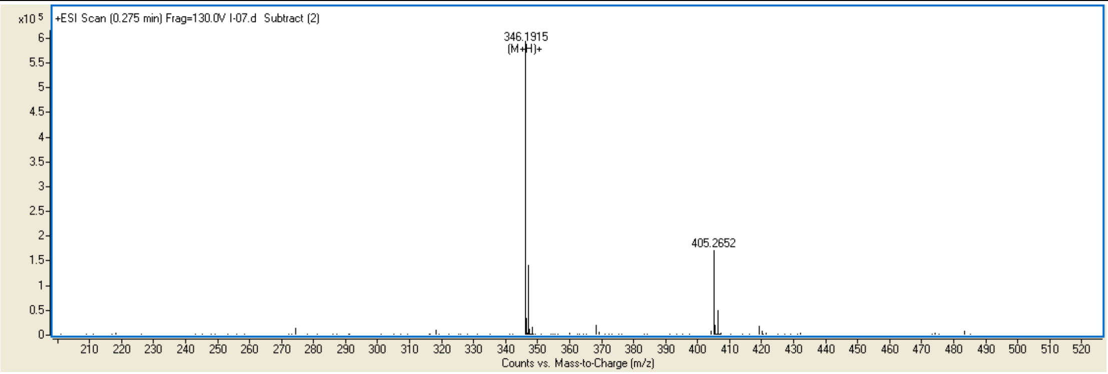

HRMS Spectrum of **6e**

Sample No.	Formula (M)	Ion Formula	Measured m/z	Calc m/z	Diff (ppm)
**6e**	$C_{21}H_{21}N_3O_2$	$C_{21}H_{21}N_3O_2$	348.1708	348.1707	0.29

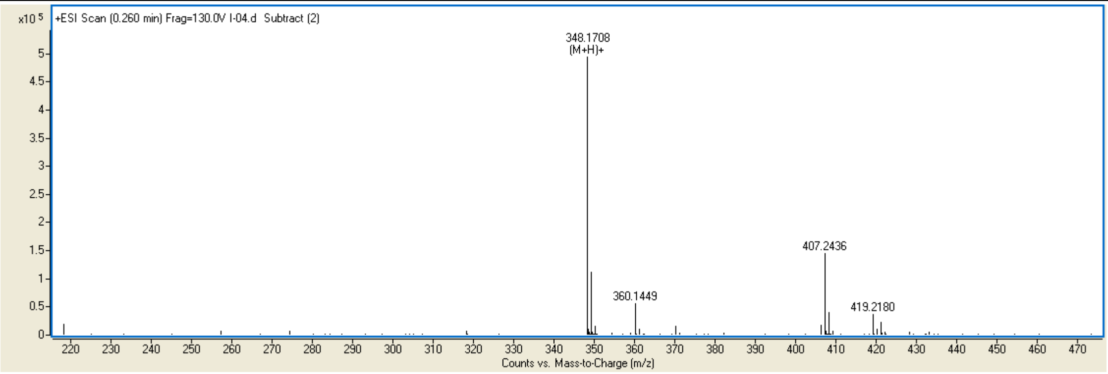

HRMS Spectrum of **6f**

Sample No.	Formula (M)	Ion Formula	Measured m/z	Calc m/z	Diff (ppm)
**6f**	$C_{24}H_{27}N_3O$	$C_{24}H_{28}N_3O$	374.2224	374.2227	0.80

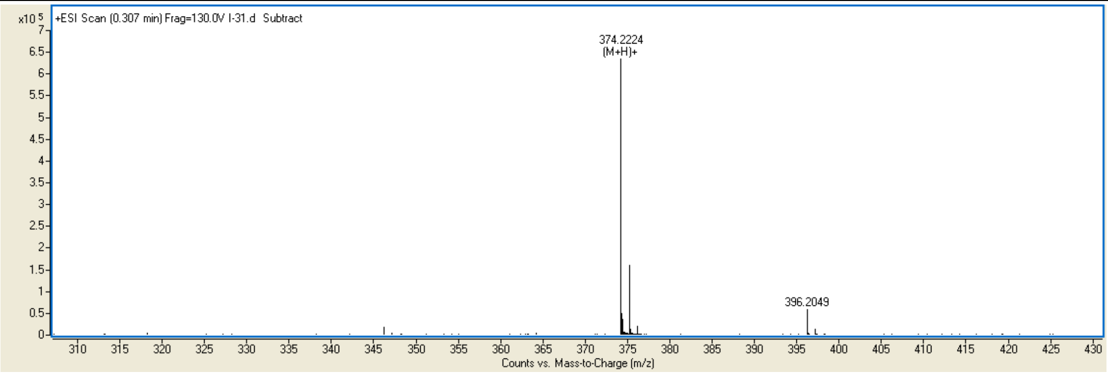

HRMS Spectrum of **6g**

Sample No.	Formula (M)	Ion Formula	Measured m/z	Calc m/z	Diff (ppm)
**6g**	$C_{21}H_{20}FN_3O$	$C_{21}H_{21}FN_3O$	350.1661	350.1663	0.57

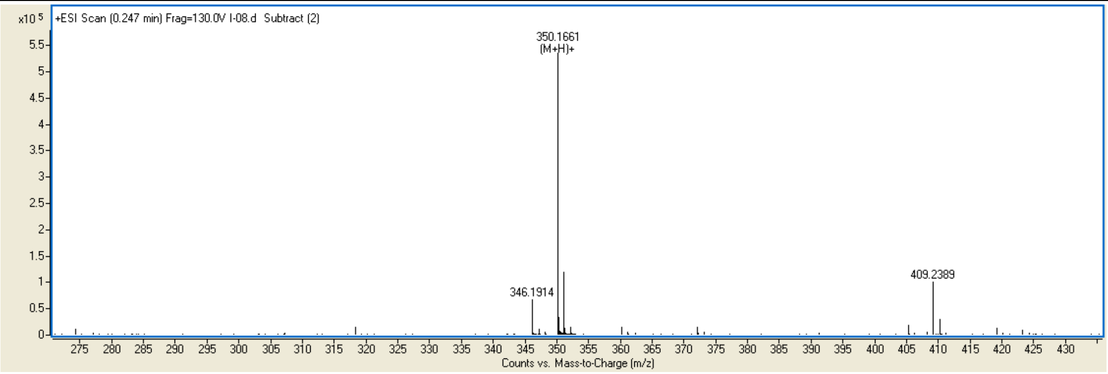

HRMS Spectrum of **6h**

Sample No.	Formula (M)	Ion Formula	Measured m/z	Calc m/z	Diff (ppm)
**6h**	$C_{21}H_{20}FN_3O$	$C_{21}H_{21}FN_3O$	350.1665	350.1663	0.57

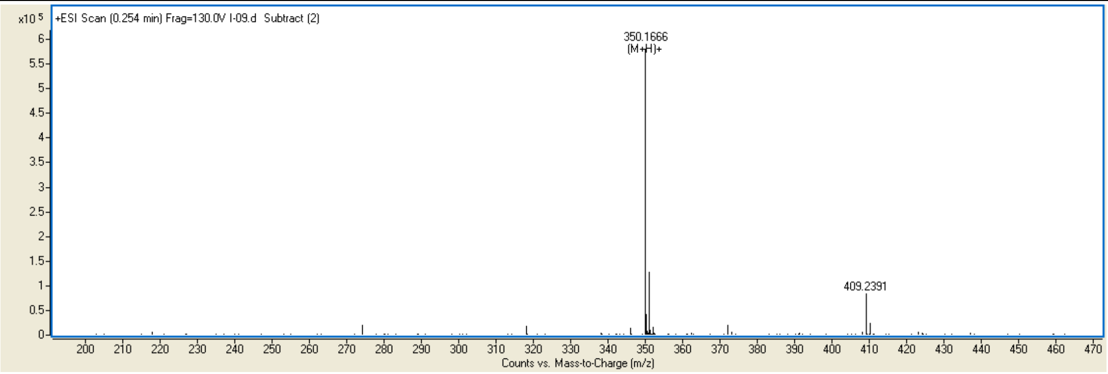

HRMS Spectrum of **6i**

Sample No.	Formula (M)	Ion Formula	Measured m/z	Calc m/z	Diff (ppm)
**6i**	$C_{21}H_{20}BrN_3O$	$C_{21}H_{21}BrN_3O$	410.0859	410.0863	0.98

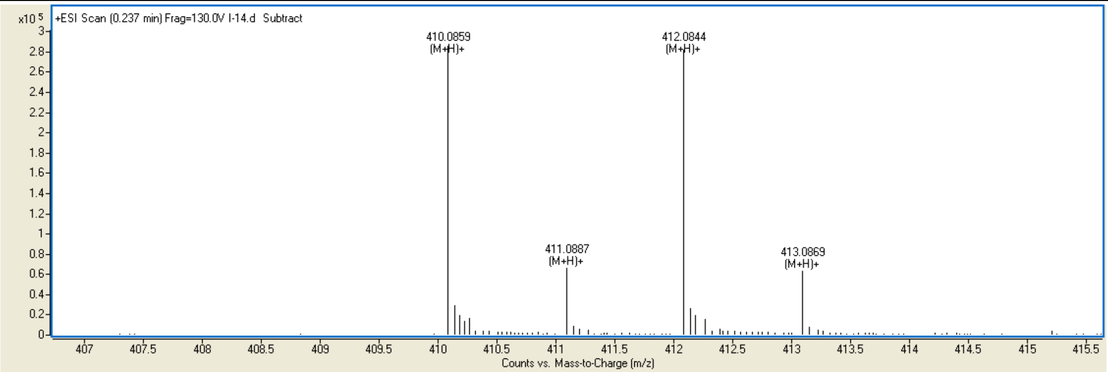

HRMS Spectrum of **6j**

Sample No.	Formula (M)	Ion Formula	Measured m/z	Calc m/z	Diff (ppm)
**6j**	$C_{21}H_{20}BrN_3O$	$C_{21}H_{21}BrN_3O$	410.0864	410.0863	0.24

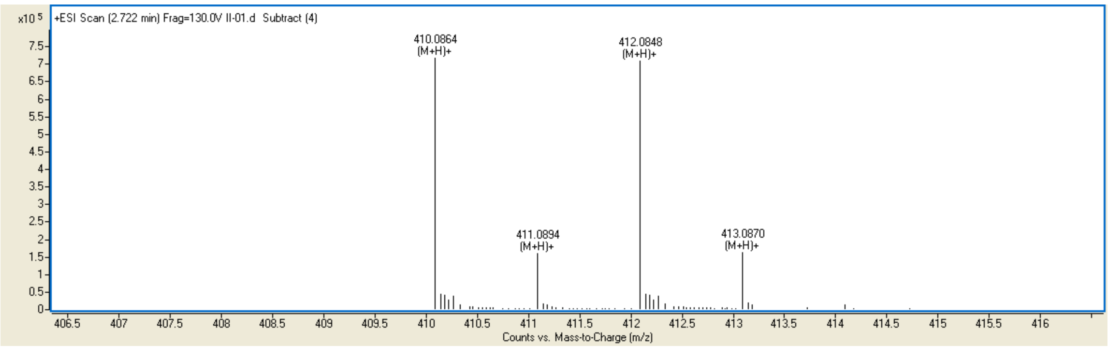

HRMS Spectrum of **6k**

Sample No.	Formula (M)	Ion Formula	Measured m/z	Calc m/z	Diff (ppm)
**6k**	$C_{22}H_{20}F_3N_3O_2$	$C_{22}H_{21}F_3N_3O_2$	416.1583	416.1580	0.72

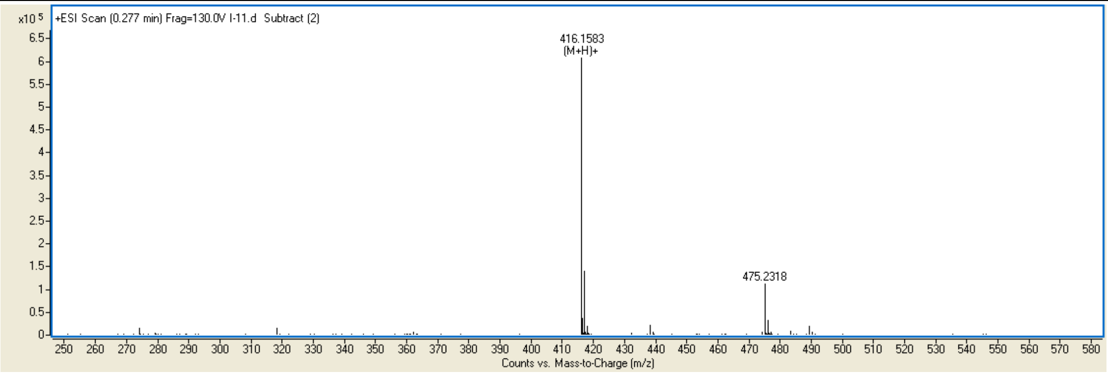

HRMS Spectrum of **6l**

Sample No.	Formula (M)	Ion Formula	Measured m/z	Calc m/z	Diff (ppm)
**6l**	$C_{22}H_{20}F_3N_3O_2$	$C_{22}H_{21}F_3N_3O_2$	416.1583	416.1580	0.72

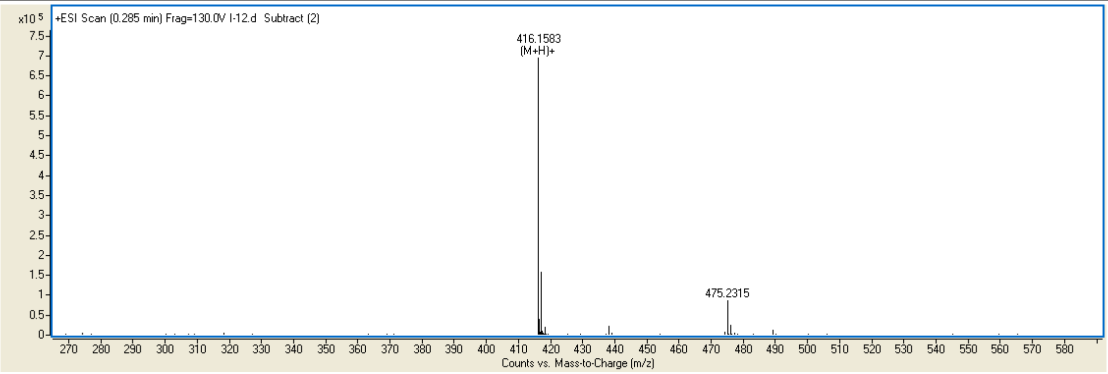

HRMS Spectrum of **6m**

Sample No.	Formula (M)	Ion Formula	Measured m/z	Calc m/z	Diff (ppm)
**6m**	$C_{22}H_{23}N_3O$	$C_{22}H_{24}N_3O$	346.1915	346.1914	0.29

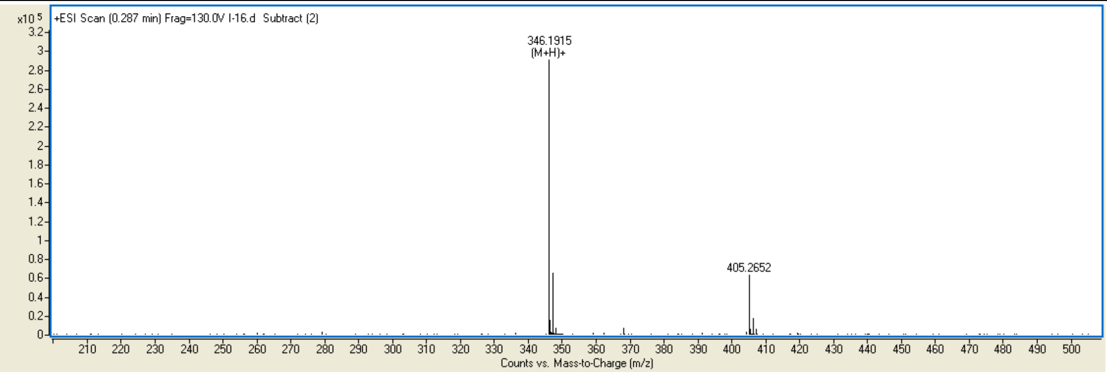

HRMS Spectrum of **6n**

Sample No.	Formula (M)	Ion Formula	Measured m/z	Calc m/z	Diff (ppm)
**6n**	$C_{22}H_{23}N_3O$	$C_{22}H_{24}N_3O$	346.1913	346.1914	0.29

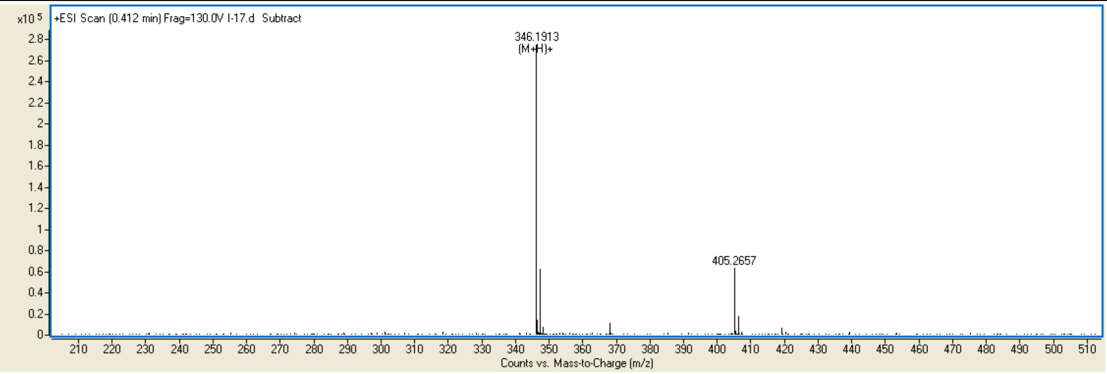

HRMS Spectrum of **6o**

Sample No.	Formula (M)	Ion Formula	Measured m/z	Calc m/z	Diff (ppm)
**6o**	$C_{23}H_{25}N_3O$	$C_{23}H_{26}N_3O$	360.2071	360.2070	0.28

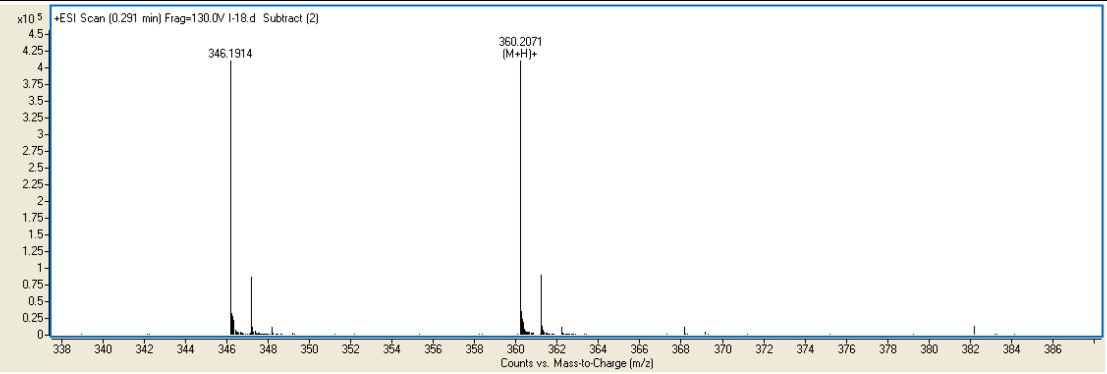

HRMS Spectrum of **6p**

Sample No.	Formula (M)	Ion Formula	Measured m/z	Calc m/z	Diff (ppm)
**6p**	$C_{22}H_{23}N_3O2$	$C_{22}H_{24}N_3O2$	362.1864	362.1863	0.28

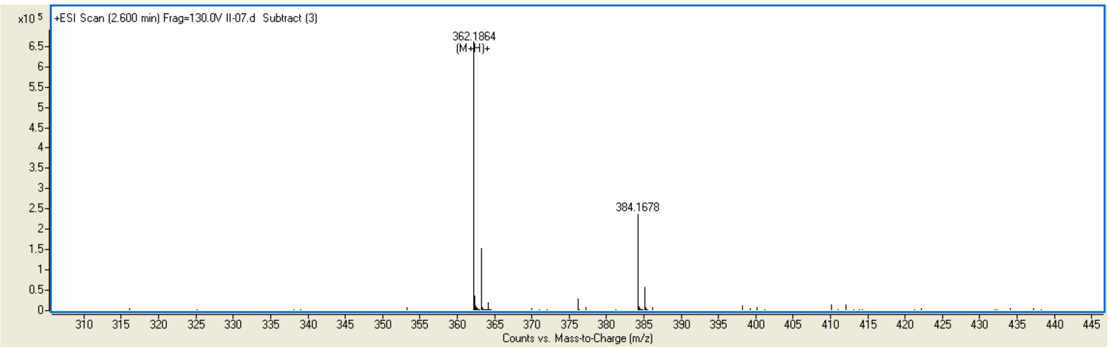

HRMS Spectrum of **6q**

Sample No.	Formula (M)	Ion Formula	Measured m/z	Calc m/z	Diff (ppm)
**6q**	$C_{25}H_{29}N_3O$	$C_{25}H_{30}N_3O$	388.2382	388.2383	0.26

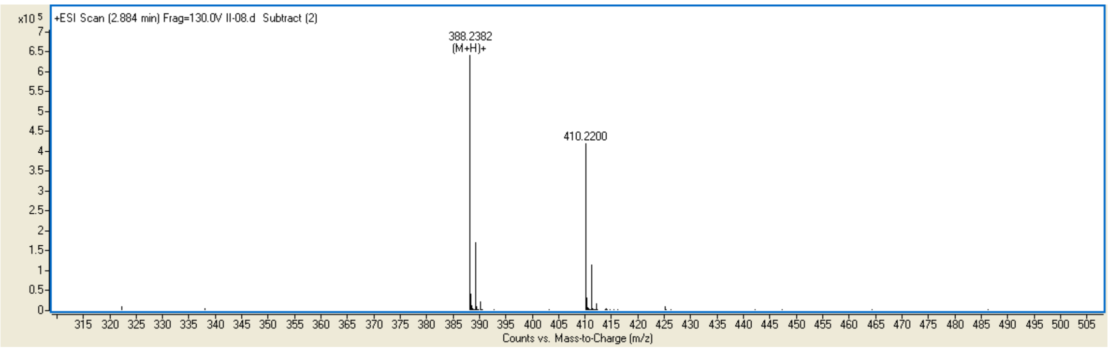

HRMS Spectrum of **6r**

Sample No.	Formula (M)	Ion Formula	Measured m/z	Calc m/z	Diff (ppm)
**6r**	$C_{22}H_{22}FN_3O$	$C_{22}H_{23}FN_3O$	364.1821	364.1820	0.27

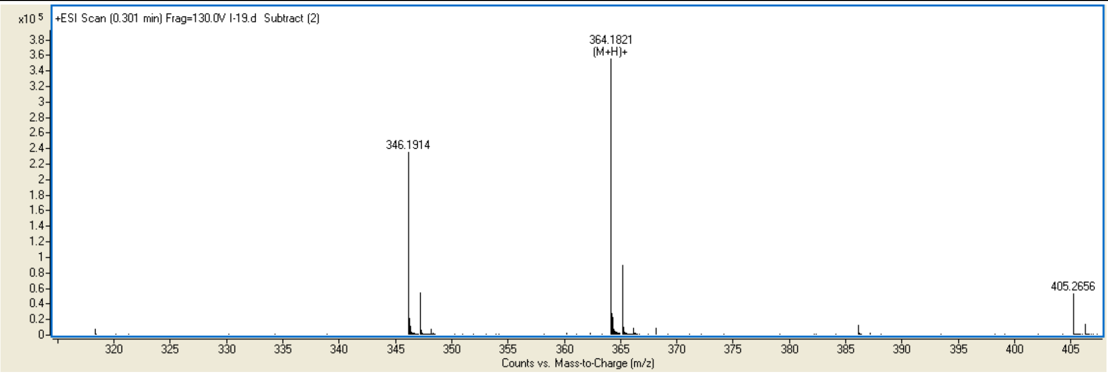

HRMS Spectrum **of 6s**

Sample No.	Formula (M)	Ion Formula	Measured m/z	Calc m/z	Diff (ppm)
**6s**	$C_{22}H_{22}FN_3O$	$C_{22}H_{23}FN_3O$	364.1821	364.1820	0.27

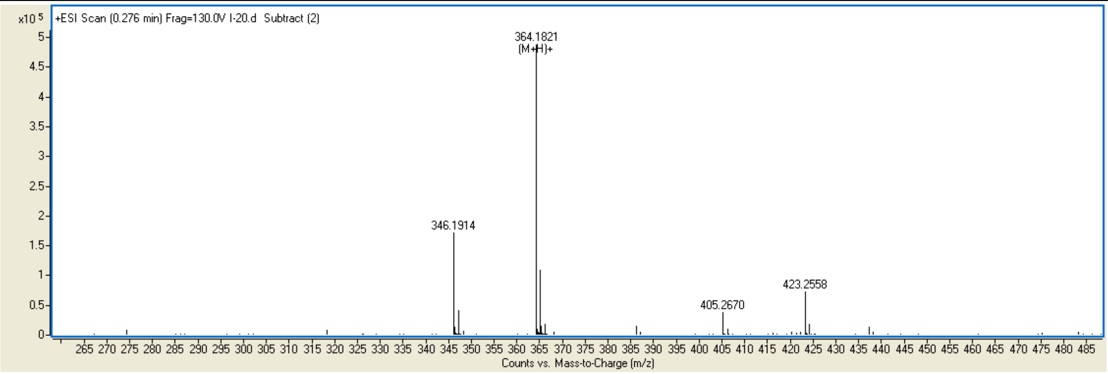

HRMS Spectrum of **6t**

Sample No.	Formula (M)	Ion Formula	Measured m/z	Calc m/z	Diff (ppm)
**6t**	$C_{22}H_{22}BrN_3O$	$C_{22}H_{23}BrN_3O$	424.1020	424.1019	0.24

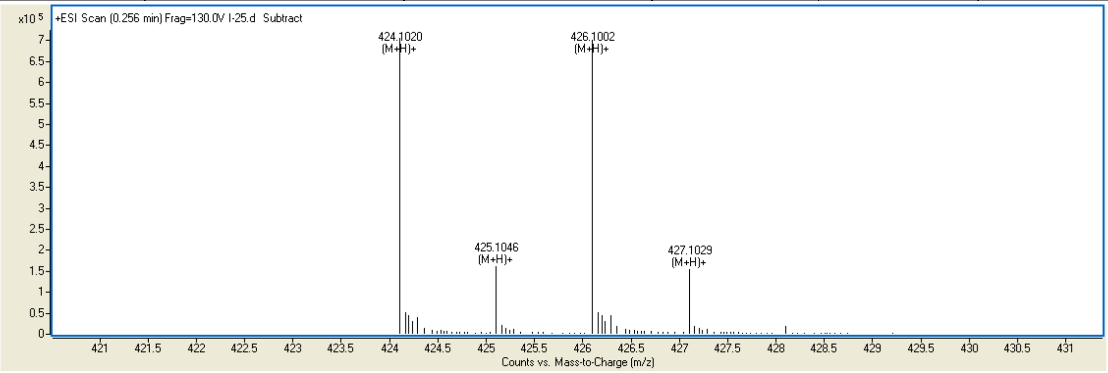

HRMS Spectrum of **6u**

Sample No.	Formula (M)	Ion Formula	Measured m/z	Calc m/z	Diff (ppm)
**6u**	$C_{22}H_{22}BrN_3O$	$C_{22}H_{23}BrN_3O$	424.1019	424.1019	0

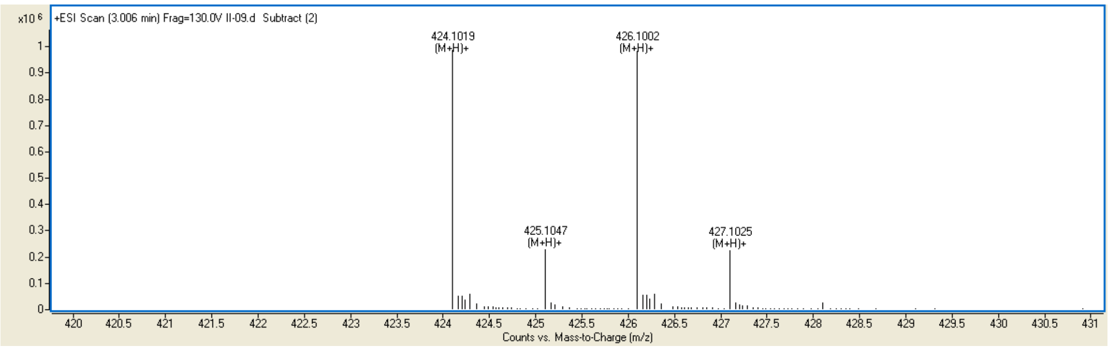

HRMS Spectrum of **6v**

Sample No.	Formula (M)	Ion Formula	Measured m/z	Calc m/z	Diff (ppm)
**6v**	$C_{23}H_{25}N_3O$	$C_{23}H_{26}N_3O$	360.2069	360.2070	0.28

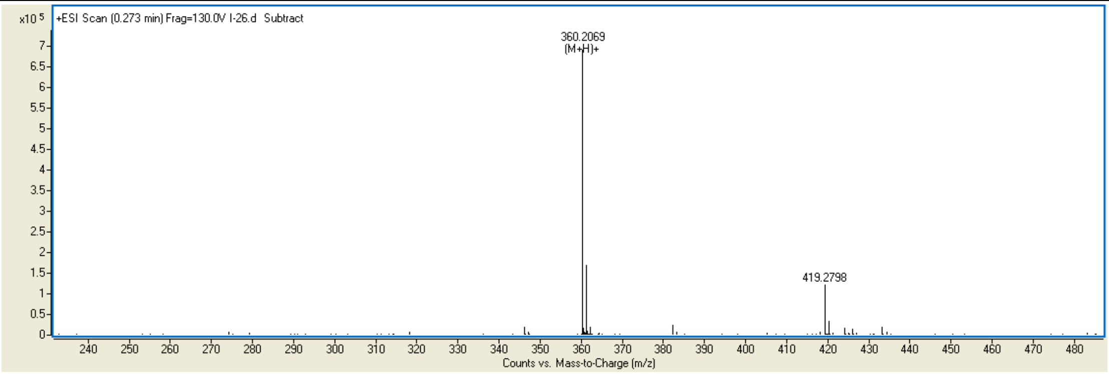

HRMS Spectrum of **6w**

Sample No.	Formula (M)	Ion Formula	Measured m/z	Calc m/z	Diff (ppm)
**6w**	$C_{24}H_{27}N_3O$	$C_{24}H_{28}N_3O$	374.2229	374.2227	0.53

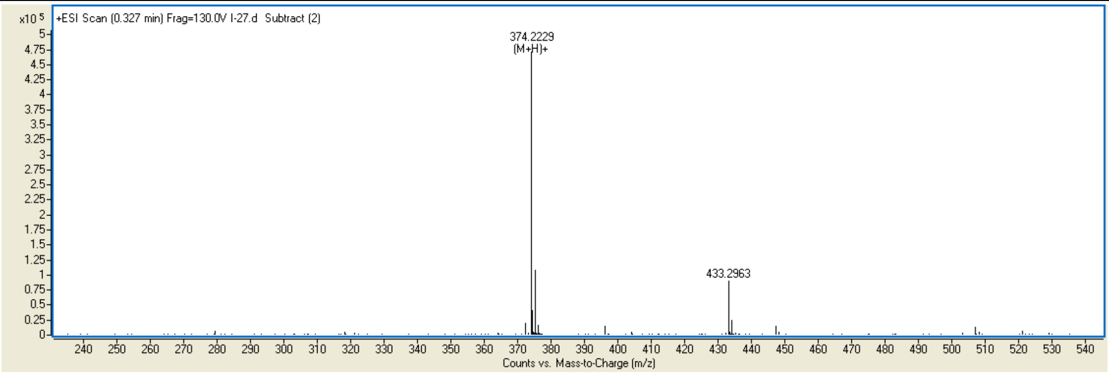

HRMS Spectrum of **6x**

Sample No.	Formula (M)	Ion Formula	Measured m/z	Calc m/z	Diff (ppm)
**6x**	$C_{23}H_{25}N_3O_2$	$C_{23}H_{25}N_3$ NaO_2	398.1840	398.1839	0.25

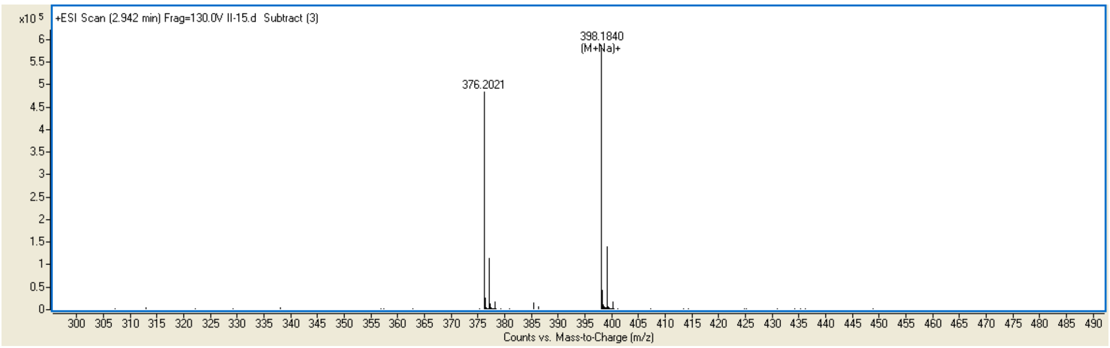

Supplement: Supplementary file 1 [file molecules-28-03741-s001.zip › molecules-2340590-supplementary.pdf]
